# Platinum(ii) complexes of mixed-valent radicals derived from cyclotricatechylene, a macrocyclic tris-dioxolene†
†Electronic supplementary information (ESI) available: Experimental procedures for the physical and computational characterisation of the compounds in this work; crystallographic figures, and tables of crystallographic parameters; spectroelectrochemical data for **[4˙]^+^** and **[5˙]^+^**; cw and pulsed EPR spectra; additional MO manifolds, orbital and spin density plots. CCDC 1037214 and 1037215. For ESI and crystallographic data in CIF or other electronic format see DOI: 10.1039/c5sc02776d


**DOI:** 10.1039/c5sc02776d

**Published:** 2015-08-20

**Authors:** Jonathan J. Loughrey, Nathan J. Patmore, Amgalanbaatar Baldansuren, Alistair J. Fielding, Eric J. L. McInnes, Michaele J. Hardie, Stephen Sproules, Malcolm A. Halcrow

**Affiliations:** a School of Chemistry , University of Leeds , Woodhouse Lane , Leeds LS2 9JT , UK . Email: m.a.halcrow@leeds.ac.uk; b Department of Chemistry , University of Sheffield , Brook Hill , Sheffield S3 7HF , UK; c School of Chemistry and Photon Science Institute , University of Manchester , Oxford Road , Manchester M13 9PL , UK; d WestCHEM , School of Chemistry , University of Glasgow , Glasgow G12 8QQ , UK . Email: stephen.sproules@glasgow.ac.uk

## Abstract

Three complexes of cyclotricatechylene (H_6_ctc), [{PtL}_3_(μ_3_-ctc)], have been synthesised: (L = 1,2-bis(diphenylphosphino)benzene {dppb}, **1**; L = 1,2-bis(diphenylphosphino)ethane {dppe}, **2**; L = 4,4′-bis(*tert*-butyl)-2,2′-bipyridyl {^*t*^Bu_2_bipy}, **3**). The complexes show three low-potential, chemically reversible voltammetric oxidations separated by *ca.* 180 mV, corresponding to stepwise oxidation of the [ctc]^6–^ catecholato rings to the semiquinonate level. The redox series **[1]^0/1+/2+/3+^** and **[3]^0/1+/2+/3+^** have been characterised by UV/vis/NIR spectroelectrochemistry. The mono- and di-cations have class II mixed valent character, with reduced radical delocalisation compared to an analogous bis-dioxolene system. The SOMO composition of **[1˙]^+^** and **[3˙]^+^** has been delineated by cw EPR, ENDOR and HYSCORE spectroscopies, with the aid of two monometallic model compounds [PtL(DBsq˙)]^+^ (DBsqH = 3,5-bis(*tert*-butyl)-1,2-benzosemiquinone; L = dppe or ^*t*^Bu_2_bipy). DF and time-dependent DF calculations confirm these interpretations, and demonstrate changes to spin-delocalisation in the ctc macrocycle as it is sequentially oxidised.

## Abstract 2

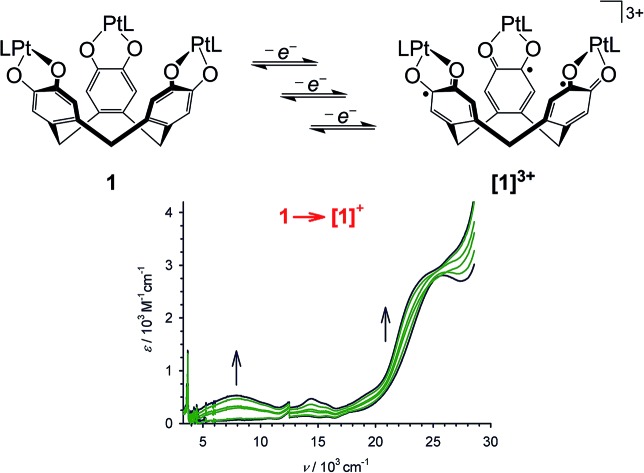
The redox series **[1]^0/1+/2+/3+^** has been characterised by UV/vis/NIR spectroelectrochemistry, cw EPR, ENDOR and HYSCORE spectroscopies and DF calculations.

## Introduction

Metal/dioxolene complexes exhibit complicated redox chemistry and spectroscopy, reflecting the accessible catecholate (‘cat’), semiquinone (‘sq’) and quinone (‘q’) oxidation states of the dioxolene group.^1^–^4^ Charge transfer between a metal ion and dioxolene is often facile,^1^ leading to intense visible/NIR absorptions that could be of use in solar energy applications.^5^ Intramolecular metal ⇌ dioxolene electron transfer can lead to valence tautomerism equilibria,^1^ which may be accompanied by a metal ion spin-state change.^6^ Alternatively, some homoleptic metal/dioxolene complexes can be obtained in mixed-valent cat/sq or sq/q ligand oxidation states, showing strong coupling and delocalisation between the ligand redox sites leading to strong NIR absorptions as before.^7^ Finally, these considerations can lead to dioxolene-related ligands and substrates acting as electron reservoirs during synthetic and biological catalysis.^8^

**Scheme 1:**
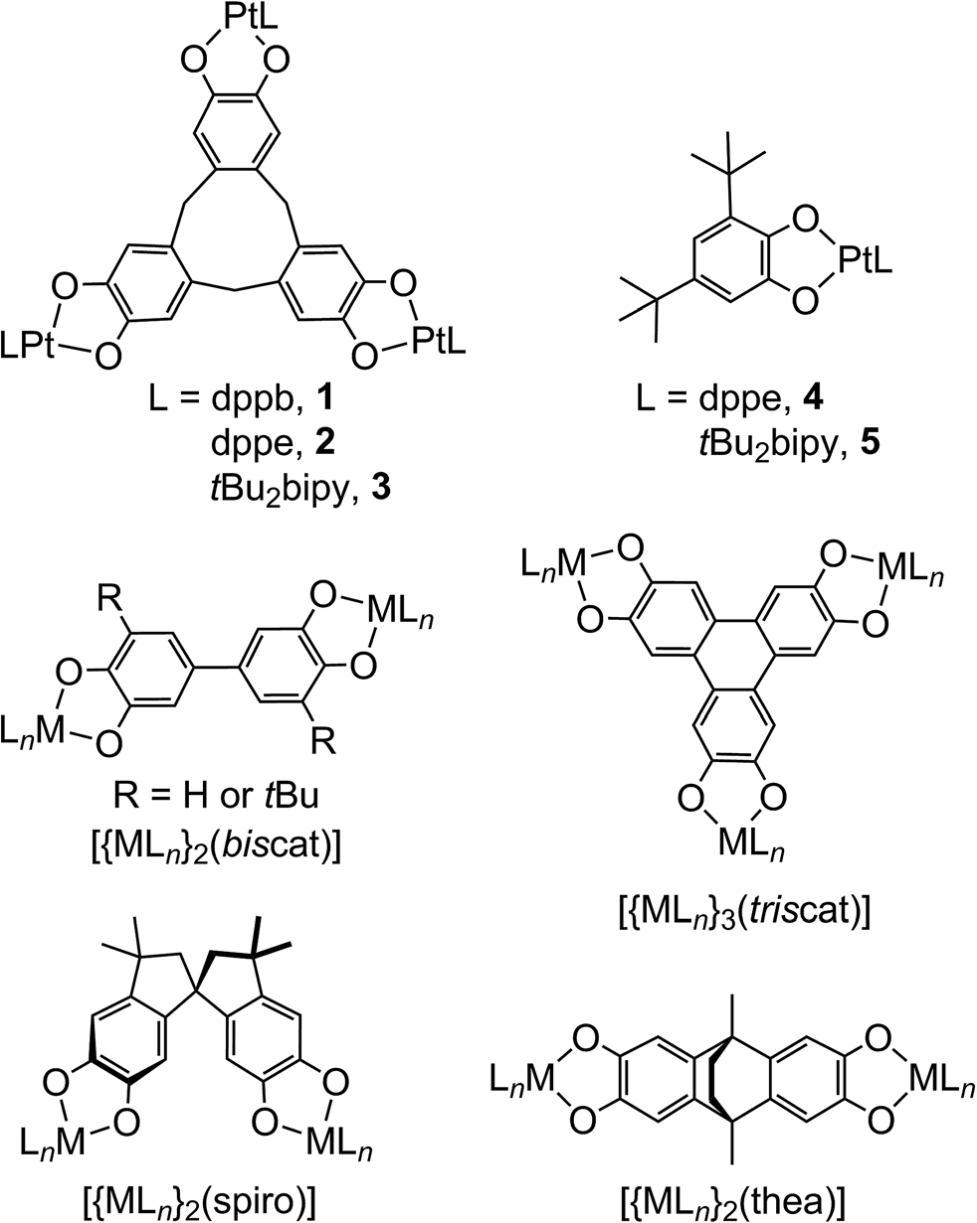
The complexes studied in this work, and other bis- and tris-dioxolene complexes referred to in the article.

Complexes of dinucleating and polynucleating dioxolenes have potential for even more complicated electronic structures and redox, involving electron transfer between multiple metal and ligand sites. Most studied are 2,5-hydroxy-1,4-benzoquinonate complexes, where two metal ions chelate to one ligand redox centre.^4^ More unusual, are complexes of ligands bearing two or more dioxolene rings separated by a spacer,^2^,^3^ which have potential for ligand-based mixed valency.^9^ Several bis- and tris-dioxolenes have been investigated in this regard, examples of which are shown in Scheme 1.^10^–^26^ However, since early interest in these compounds was focussed on the magnetic properties of molecular poly-sq radicals,^3^ only a handful of these complexed poly-dioxolenes have been spectroscopically characterised in their mixed-valent cat/sq forms.^10^–^15^,^23^ These range from being fully delocalised ([biscat˙]^3–^ 2,10–13 and [triscat˙]^5–^/[triscat˙˙]^4–^ 23) to being valence-localised, with electron hopping between the oxidised and unoxidised dioxolene rings occurring near the EPR timescale ([spiro˙]^3–^).^2^,^14^

Seventeen years ago, Bohle and Stasko communicated two compounds of type [{PtL}_3_(μ_3_-ctc)], where H_6_ctc is cyclotricatechylene (Scheme 1) and L is a diphosphine.^27^ Both compounds show three low-potential oxidations by cyclic voltammetry, indicating that the [ctc]^6–^ catecholate rings are oxidised sequentially from the cat to sq levels. No characterisation of those unusual radical species was undertaken, however. More recently cage complexes^28^ and supramolecular assemblies^29^ based on [ctc]^6–^ have been crystallographically characterised, although their redox properties also remain to be explored.

Apart from the potential of ctc complexes to act as redox-active supramolecular hosts, [ctc˙]^5–^ and [ctc˙˙]^4–^ radicals also represent a unique example of multi-center mixed valency^9^,^30^ in a cyclophane-type scaffold.^31^ Since some of us have a long-standing interest in the host:guest chemistry of receptors derived from ctc,^32^ we decided to re-investigate the redox activity of the [{PtL}_3_(μ_3_-ctc)] system. To shed further light on the trimetallic compounds, we have also quantified the electronic structures of mononuclear platinum/semiquinonate complexes by EPR and DFT methods.

## Results and discussion

Reaction of H_6_ctc with metal ion reagents is complicated by its decomposition in the presence of base, and the moisture- and air-sensitivity of its complex products. Following extensive experiments with different transition ions, Pt(ii) complexes of [ctc]^6–^ were found to be the most tractable. Thus [{PtL}_3_(μ_3_-ctc)] (L = 1,2-bis(diphenylphosphino)benzene {dppb}, **1**; L = 1,2-bis(diphenylphosphino)ethane {dppe}, **2**; L = 4,4′-bis(*tert*-butyl)-2,2′-bipyridyl {^*t*^Bu_2_bipy}, **3**) were obtained by reaction of H_6_ctc with the appropriate [PtCl_2_L] precursor in the presence of K_2_CO_3_, in a *N*,*N*-dimethylacetamide/methanol solvent mixture. Compound **1** is from Bohle and Stasko's initial study,^27^ but **2** and **3** have not been reported before. Compound **2** is less soluble than **1** in most organic solvents, which hampered its solution characterisation. The known mononuclear complexes [Pt(dppe)(DBcat)] (**4**) and [Pt(^*t*^Bu_2_bipy)(DBcat)] (**5**, Scheme 1; DBcatH_2_ = 3,5-di-*tert*-butylcatechol) were also prepared,^13^ as an aid to interpretating the spectroscopic data from **1–3**.

Electrospray mass spectra of **1–3** exhibit strong mono-, di- and tri-cationic peaks associated with intact [{PtL}_3_(μ_3_-ctc)]^*z*+^ (L = dppb, dppe or ^*t*^Bu_2_bipy; *z* = 1, 2 or 3) ions. Only moderate fragmentation through loss of L or {PtL}^2+^ fragments was observed (ESI†). The ^1^H NMR spectra of **1–3** show the anticipated distribution of peaks, with a characteristic pair of resonances from the ditopic CH_2_ groups in the [ctc]^6–^ macrocycle. The spectra are sharp when obtained under an inert atmosphere, but are broadened when run in air with reduced integrals for the [ctc]^6–^ ligand peaks. X-band EPR of the air-exposed solutions showed a weak resonance at g ≈ 2.03 at 150 K, implying the NMR peak-broadening reflects partial aerobic oxidation of the coordinated [ctc]^6–^.

**Fig. 1:**
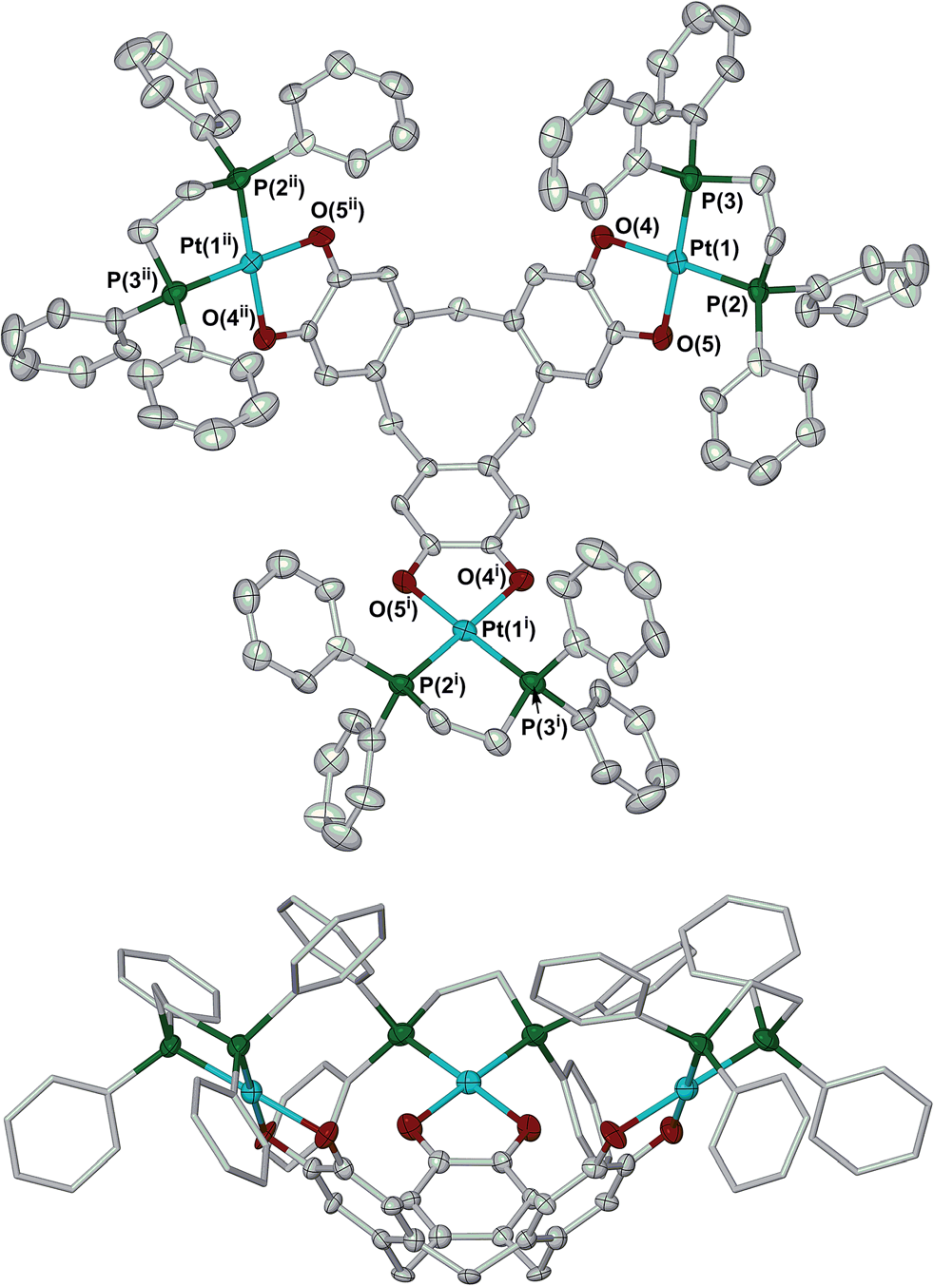
Two views of the complex molecule in **2**·H_2_O·8dma. Displacement ellipsoids are at the 50% probability level, except for the dppe C atoms in the lower view which are de-emphasised, and H atoms are omitted for clarity. Colour code: C, white; O, red; P, green; Pt, cyan. Symmetry codes: (i) 1 – *y*, 1 + *x* – *y*, *z*; (ii) –*x* + *y*, 1 – *x*, *z*.

Crystallisation of **2** from *N*,*N*-dimethylacetamide (dma)/methanol yielded a mixture of two solvates. The complex molecule in **2**·H_2_O·8dma has crystallographic *C*_3_ symmetry, and is disordered about a mirror plane. Its molecular structure resembles that of **1** (Fig. 1).^27^ The bond lengths and angles within the metal coordination sphere are typical for a platinum(ii)/catecholate/phosphine complex,^33^ while the dimensions of the unique dioxolene ring provide no evidence for oxidation of the [ctc]^6–^ ligand (ESI†).^34^ Like all ctc derivatives,^32^**2** has a bowl-shaped cavity at its centre, of approximate dimensions 6.8 Å rim diameter × 9.5 Å depth. Precedent implies this cavity should be occupied by solvent;^25^,^35^,^36^ although this was not apparent in the Fourier map, a *SQUEEZE* analysis^37^ implied the presence of two unresolved molecules of dma in this region of the lattice. The dihedral angle between the catecholate groups in the molecule is 71.2(5)°, which is again similar to the published solvate of **1** (70.6–73.2°).^27^

The complex molecule in the other solvate, **2**·2H_2_O·1.3dma·0.5MeOH, lies on a general crystallographic site. The molecular structure of **2** in this crystal is very similar to the first solvate, apart from differences in the orientations of some phenyl substituents. In particular, the dimensions of the ctc dioxolene units are again consistent with the catecholate oxidation level (ESI†).^34^ The depth of the molecular cavity in this solvate is *ca.* 9.2 Å, but its rim dimensions cannot be uniquely defined because of disorder in the dppe phenyl groups. The cavity contains a disordered dma molecule, which is incompletely resolved in the model. The intramolecular dihedral angles between neighbouring catecholate groups in this solvate span a wider range at 62.9(3)–76.2(3)°, implying some flexibility in the [ctc]^6–^ macrocycle. The average of these angles, 69.1(5)°, is similar to the value from the first crystal however.

### Electrochemistry and spectroelectrochemistry

**Scheme 2:**
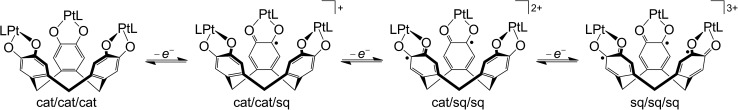
Ligand-based redox series exhibited by [{PtL}_3_(μ_3_-ctc)] (L = neutral bidentate ligand).

**Fig. 2:**
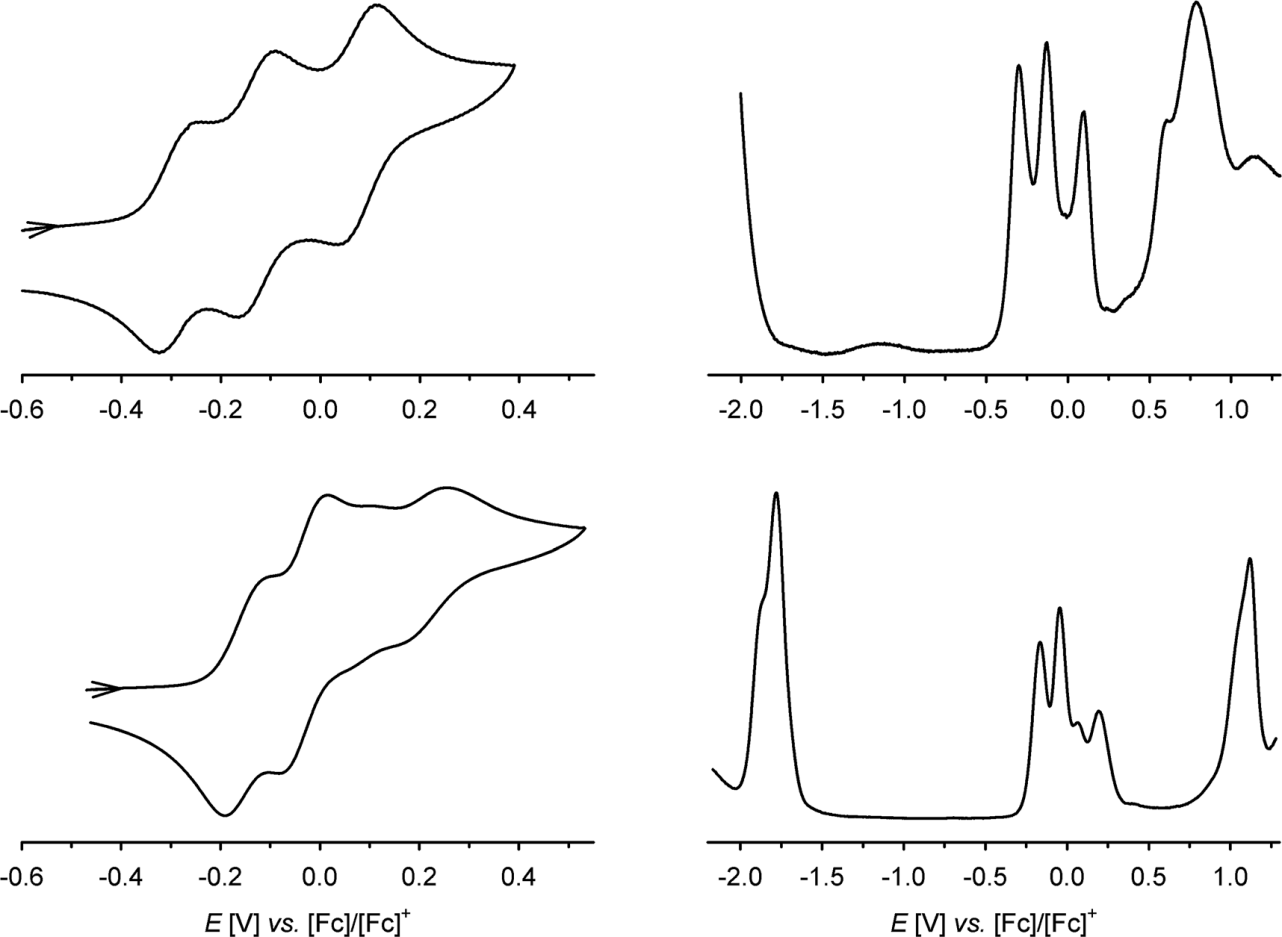
Cyclic and differential pulse voltammograms of **1** (top) and **3** (bottom) at 298 K, in CH_2_Cl_2_/0.5 M [N^*n*^Bu_4_]BF_4_ and scan rate 100 mV s^–1^. Only the cat/sq oxidation window is shown in the CVs, while the DPVs scan the full range of the solvent window.

The cyclic voltammogram (CV) and differential pulse voltammogram (DPV) of **1** in CH_2_Cl_2_/0.5 M [^*n*^Bu_4_N]BF_4_ at 298 K confirmed Bohle and Stasko's original report, in showing the [ctc]^6–/5–/4–/3–^ redox series (cat/cat/cat ⇌ cat/cat/sq ⇌ cat/sq/sq ⇌ sq/sq/sq, Scheme 2; Table 1, Fig. 2).^27^ These oxidations in **2** occur at similar potentials as in **1**, although **2** also exhibits a fourth process near +0.2 V that is not shown by **1**. The CV and DPV of **3** resemble those of **2**, albeit with slightly more positive potentials for the first two oxidations. The third and fourth oxidation processes in **2** and **3** lie at almost identical potentials and have *ca.* half the intensity of the first two processes by DPV (Fig. 2). Since its relative intensity is reduced at higher scan rates, we tentatively attribute the fourth oxidation to a daughter arising from deposition of **[2˙˙]^2+^** and **[3˙˙]^2+^** at the electrode surface. That would explain why such a peak is not observed for **1**, which is more soluble than **2** in the base electrolyte medium. The separation between the first three oxidation potentials in **1** and **2**, Δ*E*_1/2_ = 0.18–0.22 V (*K*_c_ = 1–5 × 10^3^),^30^ is larger than in **3** (0.12 V, *K*_c_ = 1 × 10^2^) and implies moderate-to-weak communication between the dioxolene rings in these complexes. In comparison, complexes of [thea]^4–^ and [spiro]^4–^, whose dioxolene rings are also linked by methylene spacers (Scheme 1), show Δ*E*_1/2_ (*K*_c_) = 0.25 V (2 × 10^4^) and *ca.* 0.15 V (3 × 10^2^) respectively.^2^,^14^,^15^

**Table 1:** Electrochemical data for the complexes^*a*^

	sq/cat (*E*_1/2_/V)			q/sq (irr^*b*^, *E*p_a_/V)	[^*t*^Bu_2_bipy]^0/–^(*E*_1/2_/V)
**1** ^*c*^	–0.30	–0.12	+0.10	+0.62	—
**2**	–0.35	–0.17	+0.04, +0.23	+0.61^*d*^, +0.79	—
**3**	–0.17	–0.05	+0.07, +0.20	+1.12	–1.78, –1.86^*d*^

^*a*^Potentials referenced to Fc^+/0^.

^*b*^irr = irreversible.

^*c*^cat/sq *E*_1/2_ = +0.04, 0.24 and 0.49 V *vs.* NHE from ref. 27.

^*d*^Shoulder.

In addition to these low-potential oxidations, **1–3** exhibit an envelope of irreversible oxidations between +0.6 and +1.1 V, attributable to the [ctc]^3–/2–/1–/0^ (sq/sq/sq ⇌ sq/sq/q ⇌ sq/q/q ⇌ q/q/q) sequence, while **3** also exhibits a chemically reversible three-electron reduction at –1.8 V (Fig. 2). This arises from reduction of the ^*t*^Bu_2_bipy co-ligands,^13^,^38^–^43^ and is split into at least two components in the DPV trace.

**Fig. 3:**
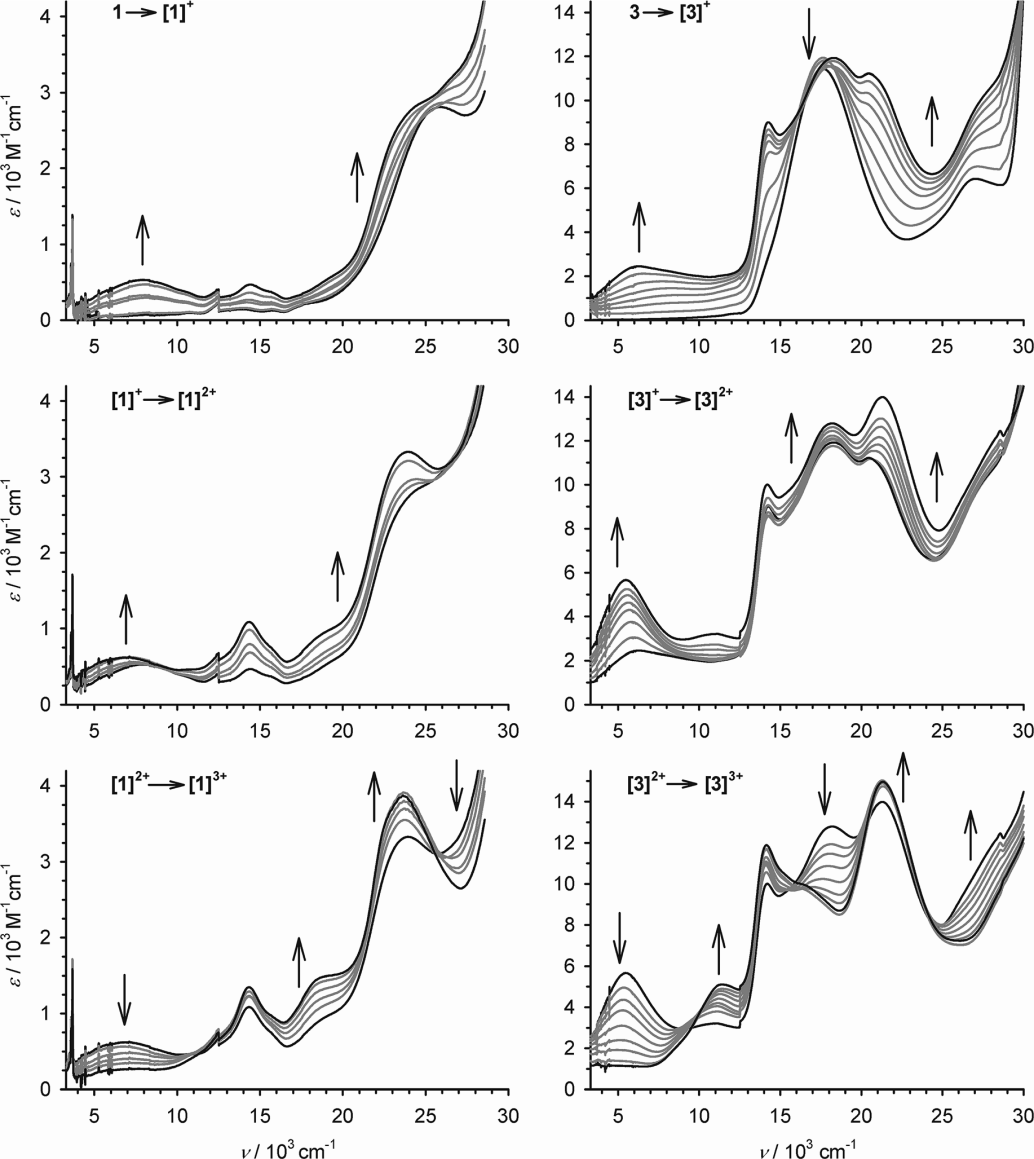
The first three oxidations of **1** (left) and **3** (right) at 253 K, in CH_2_Cl_2_/0.1 M NBu_4_BF_4_, monitored by UV/vis/NIR spectroscopy using an optically transparent electrode. The spectra of the pure starting material and product spectra are highlighted in black while the intermediate spectra are paler. Discontinuities near 12.4 and 28.7 × 10^3^ cm^–1^ are artifacts from grating changes in the spectrometer.

The oxidation processes of **1** and **3** were monitored spectro-electrochemically in CH_2_Cl_2_/0.5 M [N^*n*^Bu_4_]BF_4_ at 253 K (Fig. 3). Three of the oxidations have no potential isosbestic points, but the other oxidations exhibit minor decomposition of the compounds during the experiments. Their small potential separation (Δ*E*) also made it difficult to poise the samples cleanly at the intermediate oxidation levels. Despite these issues, however, the following trends can be noted.

Oxidation of **1** to **[1˙]^+^** leads to the appearance of a broad near-IR absorption centred at 7.9 × 10^3^ cm^–1^, assignable to an intervalence charge transfer (ICVT) band (Fig. 3). Generation of the **[1˙]^+^** → **[1˙˙]^2+^** oxidation causes the IVCT band to shift to *ν*_max_ = 7.1 × 10^3^ cm^–1^, while increasing slightly in intensity. The **3** → **[3˙]^+^** → **[3˙˙]^2+^** sequence also generates an IVCT absorption, which also red-shifts by 800 cm^–1^ during the second oxidation but is stronger and lower energy than for **[1˙]^+^**–**[1˙˙]^2+^** (Fig. 3; Table 2). Generation of the third oxidation of both compounds almost completely quenches the near-IR absorption. Assignment of the other spectral changes was aided by comparison with the mono-metallic complexes (see below). A weak absorption at 11.1 × 10^3^ cm^–1^ in **[3˙˙]^2+^** and **[3˙˙˙]^3+^** may be a Pt(dσ) → sq MLCT transition, and a strong peak at 20–24 × 10^3^ cm^–1^ in all the radical spectra is assigned to a Pt(dπ) → sq MLCT band.^44^ A transition near 18 × 10^3^ cm^–1^, which is shown by **3**–**[3˙˙]^2+^** but collapses upon the third oxidation, is a cat → bipy inter-ligand CT band,^39^ while a peak at 27.2 × 10^3^ cm^–1^ in **3** that blue-shifts upon oxidation is a Pt → bipy MLCT.^41^,^42^ More detailed assignment of these peaks is described below.

**Table 2:** Electronic absorption data for the complexes

	*ν*_max_/10^3^ cm^–1^ (*ε*_max_/10^3^ M^–1^ cm^–1^)
**1**	13.9 (0.1), 15.7 (0.1), 17.7 (sh), 26.3 (2.8)
**[1˙]^+^**	7.9 (0.5), 14.4 (0.4), 15.9 (sh), 24.7 (sh)
**[1˙˙]^2+^**	7.1 (0.7), 14.3 (1.1), 15.7 (sh), 18.6 (sh), 23.5 (3.3)
**[1˙˙˙]^3+^**	10.6 (sh), 14.3 (1.4), 18.6 (sh), 23.6 (3.9)
**2**	14.6 (0.5), 26.9 (sh)
**3**	14.1 (sh), 17.6 (11.6), 27.2 (6.4)
**[3˙]^+^**	6.2 (2.4), 14.2 (9.0), 18.1 (11.9), 20.8 (11.1), 27.2 (sh)
**[3˙˙]^2+^**	5.4 (5.7), 11.0 (3.2), 14.2 (10.0), 18.2 (12.8), 21.1 (14.0)
**[3˙˙˙]^3+^**	11.4 (5.0), 14.1 (11.7), 16.3 (sh), 21.3 (14.7)
**4**	28.2 (sh), 33.2 (6.6)
**[4˙]^+^**	12.3 (0.1), 14.3 (0.2), 16.0 (0.2), 18.7 (0.5), 24.0 (3.4)
**5**	16.8 (4.0), 26.0 (1.7)
**[5˙]^+^**	10.4 (0.1), 16.0 (sh), 17.0 (2.0), 20.5 (sh), 21.6 (5.3), 25.3 (sh), 28.5 (sh), 29.7 (sh)

Despite the non-isosbesticity of the electrochemical oxidations, re-reduction of the fully oxidised solutions at –0.4 V regenerated the neutral precursor spectra with transient appearance of the IVCT peaks described above. That implies **1** and **3** spontaneously reassemble from their regenerated constituents.

Allowing for overlapping high-energy absorptions,^45^ the width at half height (Δ*ν*_1/2_) of the IVCT bands in **[3˙]^+^** and **[3˙˙]^2+^** can be estimated at 4950 and 3550 cm^–1^, respectively. In comparison, the values predicted for a class II mixed-valent system^46^ with these IVCT energies are Δ*ν*_1/2_ ≈ 3800 cm^–1^ for **[3˙]^+^** and 3500 cm^–1^ for **[3˙˙]^2+^** (eqn (1)).^9^,^30^

1 Δ*ν*_1/2_ = (2310*E*)^1/2^

Δ*ν*_1/2_ for **[1˙]^+^** and **[1˙˙]^2+^** cannot be measured directly but is greater than 5000 cm^–1^, which is also larger than predicted by eqn (1) (4250 cm^–1^ for **[1˙]^+^** and 4050 cm^–1^ for **[1˙˙]^2+^**). Assuming they lie in the class II limit, the IVCT *ν*_max_ for the mixed-valent radicals corresponds to their inter-valence reorganisation energy (*λ*); that is, 5.4 ≤ *λ* ≤ 7.9 × 10^3^ cm^–1^ (Table 2).^30^ Those are at the low end of the typical range of *λ* values for class II organic radicals.^9^ UV/vis/NIR spectra in different solvents to define *λ* in more detail were not undertaken,^9^,^30^ because of the limited stability of the oxidised complexes. None-the-less, all these mixed-valent radicals adopt electronic structures whose dioxolene rings are partly valence-localised, although **[3˙˙]^2+^** may exhibit a reduced barrier to electron-hopping since its Δ*ν*_1/2_ value is closer to the classical class II description.^9^,^30^,^46^ That contrasts with the cat/sq radicals [{PtL}_2_(thea˙)]^+^ (L = dppb or dppe; Scheme 1) which exhibit narrower IVCT bands that are more characteristic of the class III formalism.^15^

Mononuclear **4** and **5** undergo a reversible cat → sq oxidation near –0.2 V *vs.* [Fc]^+/0^, which was monitored by chemical redox titration with [Fc]PF_6_. The changes in the UV/vis spectra of **4** and **5** upon oxidation resemble the first oxidations of the trinuclear compounds (Fig. S9;† Table 2), in depletion of cat → L LLCT absorptions (L = dppe or ^*t*^Bu_2_bipy) and the growth of new Pt(dσ) → sq and Pt(dπ) → sq MLCT peaks near 12 and 23 × 10^3^ cm^–1^, respectively.^13^,^44^ The energy difference between the Pt(dπ) → sq band in **[4˙]^+^** compared to **[5˙]^+^** (Δ*E*_MLCT_ = 2.4 × 10^3^ cm^–1^) is identical to the difference between the same transition in **[1˙]^+^** and **[3˙]^+^** (2.4 × 10^3^ cm^–1^), and **[1˙˙]^2+^** and **[3˙˙]^2+^** (2.3 × 10^3^ cm^–1^; Table 2). That implies the Pt/sq interactions in the ctc-based radicals and the mono-nuclear semiquinonate complexes are similar in character.

### EPR spectroscopy of monometallic complexes

**Fig. 4:**
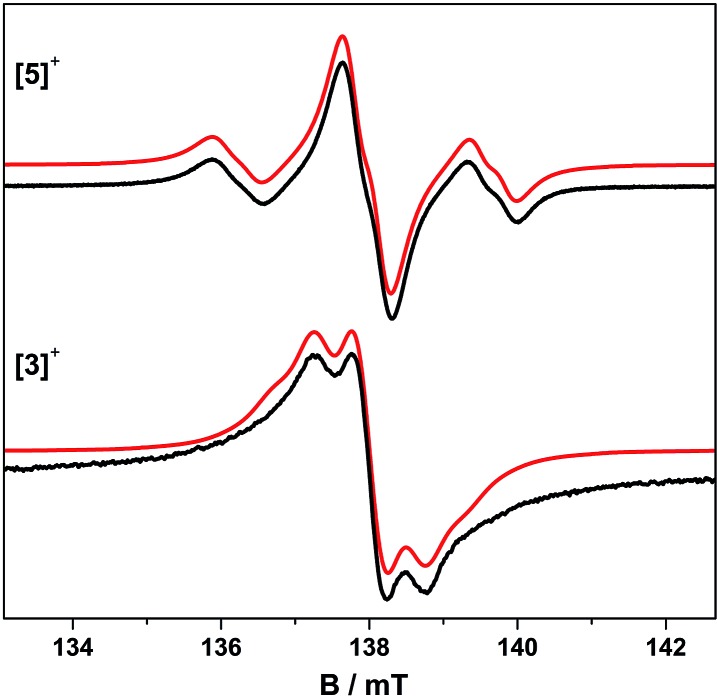
Fluid solution S-band EPR spectra of **[5˙]^+^** and **[3˙]^+^** in CH_2_Cl_2_/THF at 230 K. Simulations (red) used the parameters in Table 3.

Fluid solution S-band (3.9 GHz) and X-band (9.4 GHz) EPR spectra of **[4˙]^+^** and **[5˙]^+^** (Scheme 1) in dichloromethane/THF solution at 210–230 K exhibit one central line from naturally occurring metal isotopes with no nuclear spin, flanked by ^195^Pt (*I* = 1/2, 33.8% abundant) hyperfine satellites. The isotropic ^195^Pt coupling is much larger in **[5˙]^+^** than in **[4˙]^+^** (96.4 *vs.* 36.6 MHz, respectively). In **[4˙]^+^**, there is further hyperfine to one ^1^H and two equivalent ^31^P nuclei (*a*^H^ = 9.5, *a*^P^ = 7.8 MHz; Fig. S10,† Table 3). For **[5˙]^+^**, there is splitting to a single ^1^H nucleus, with *a*^H^ = 9.4 MHz (Fig. 4, Table 3). The *g* ≈ 2.002 value and hyperfine analysis (see below) for both radicals identify the singly occupied molecular orbital (SOMO) as largely localised in the dioxolene ligand π-system, but with some contribution from metal-based orbitals. The strongly coupled proton is assigned to *H*4 of the [DBsq˙]^–^ ligand (HDBsq = 3,5-di(*tert*-butyl)-1,2-benzosemiquinone),^47^ since the *C*4,5 positions carry much greater spin density than *C*3,6 (see below).

**Table 3:** Spin–Hamiltonian parameters (MHz) and Pt contribution to the SOMO for the radical complexes

		**[1˙]^+^**	**[3˙]^+^**	**[4˙]^+^**	**[5˙]^+^**
	*g*_iso_	2.0019	2.0017	2.0022	2.0025
	*g*_*y*_	2.0112	2.0406	2.0095	2.0403
	*g*_*x*_	2.0088	2.0032	2.0062	2.0036
	*g*_*z*_	1.9838	1.9507	1.9789	1.9509
^195^Pt	*A*_iso_	≈–12^*a*^	-31	-37	-97
	*A*_*y*_	-70	-100	-66	-111
	*A*_*x*_	-71	-144	-78	-150
	*A*_*z*_	+33^*b*^	-35^*b*^	+33^*b*^	-32^*b*^
^1^H^*c*^	*a*_iso_			9.5	9.4
	*a*_*x*_			^*d*^	15
	*a*_*y*_			^*d*^	2.6
	*a*_*z*_			^*d*^	10.6
	*a*				9.4
	*γ* ^*e*^ (deg)				60
^1^H^*f*^	*a*_*x*,*y*_	1.4	2.0	^*d*^	0.9
	*a*_*z*_	2.6	3.2		3.6
	*a*	1.8	2.4		1.8
^1^H^*g*^	*a*_*y*,*z*_			^*d*^	5
	*a*_*x*_				7
^31^P	*a*_iso_	2.7^*b*^		7.8	
	*a*_*x*,*y*_	8.5		8.2	
	*a*_*z*_	8.0		7.6	
	*a*	8.3		8.0	
^14^N	*a*_iso_		1.0		1.2
	*K*		0.36		0.38
	*a* ^2^ ^*h*^	0.001	0.033	0.009	0.028
	*b* ^2^ ^*h*^	0.071	0.044	0.069	0.055
	Total Pt	0.078	0.074	0.078	0.083
	DFT	0.043	0.050	0.040	0.074

^*a*^Value in fluid solution is *ca.* 1/3 as large as the average value in frozen solution. See text for details.

^*b*^From *A*_iso,*x*,*y*_.

^*c*^ *H*4 of 3,5-DBsq.

^*d*^Similar to those in **[5˙]^+^**.

^*e*^Euler angle about *z* relating **a**_**H**_ and **g** frames.

^*f*^ *H*6 of DBsq or *H*3,6 of ctc (modelled as axial from HYSCORE, but orientation not well defined).

^*g*^Unassigned.

^*h*^Pt 5d_*yz*_ and 6p_*z*_ admixture to SOMO.

**Fig. 5:**
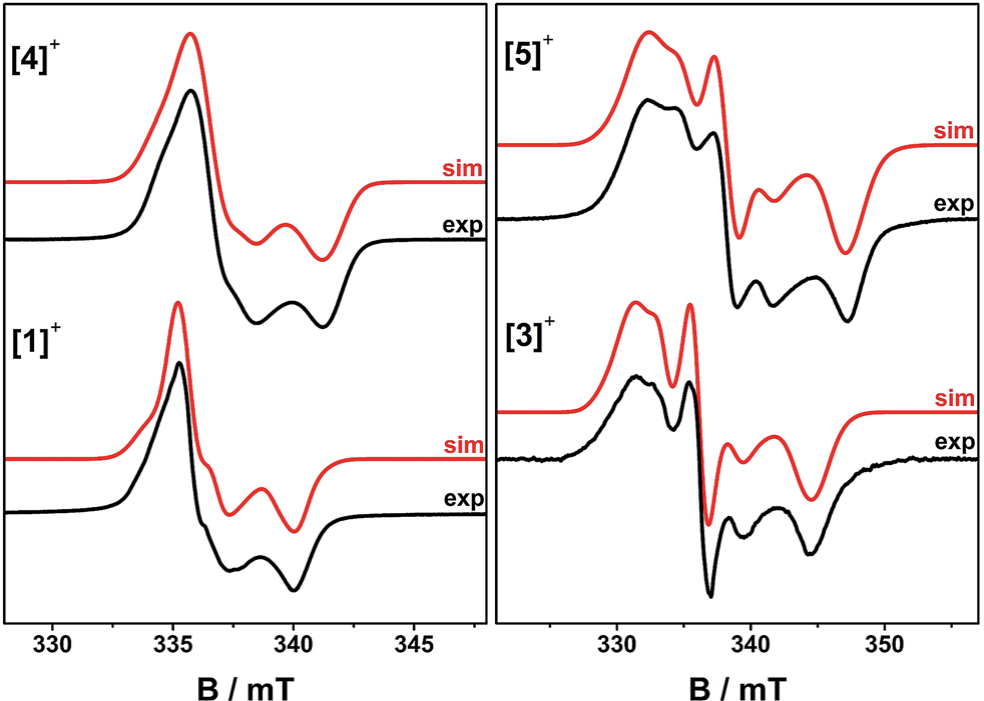
Frozen solution X-band EPR spectra of **[1˙]^+^** and **[3˙]^+^** at 150 K, and **[4˙]^+^** and **[5˙]^+^** at 30 K in CH_2_Cl_2_/THF. Simulation parameters are given in Table 3.

EPR spectra of frozen solutions were recorded at S-, X- and Q-band (3.9, 9.4 and 33.7 GHz, respectively) to determine the **g** and ^195^Pt **A** matrices. The spectra are near-axial for **[4˙]^+^** but clearly rhombic for **[5˙]^+^** (Fig. 5, S10 and S11†) and are dominated by ^195^Pt hyperfine coupling, as is common for Pt(ii) complexes of π-radical ligands.^41^,^42^,^48^–^50^ Following the literature analyses,^41^,^42^,^48^,^51^ the molecular *z*-axis is defined perpendicular to the plane of the molecule, and the *y*-axis as bisecting the dioxolene ligand bite angle (Fig. S12†) with the assignment of the principal *g*-values in Table 3. The ^195^Pt *A*_*x*_ and *A*_*y*_ components are resolved, but the high-field *A*_*z*_ component is not. This was therefore derived from *A*_*z*_ = 3*A*_iso_ – (*A*_*x*_ + *A*_*y*_). This component has an estimated uncertainty of 12 MHz, but clearly has a different sign to *A*_iso,*x*,*y*_ for **[4˙]^+^**. In contrast, all the hyperfine components in **[5˙]^+^** have the same sign.

Approximating the molecular symmetry to *C*_2v_, the SOMO has *b*_2_ symmetry (being dominated by the [DBsq˙]^–^ ligand; see below). In this case only the metal 5d_*yz*_ and 6p_*z*_ valence orbitals have the correct symmetry to admix.^41^,^42^,^48^,^50^,^52^,^53^ These contributions can be calculated from the ^195^Pt hyperfine matrix *via*eqn (2)–(4),^41^,^42^ where: *a*^2^ and *b*^2^ are the 5d_*yz*_ and 6p_*z*_ admixtures to the SOMO, respectively; *P*_d_ and *P*_p_ are the electron nuclear dipolar coupling parameters for Pt 5d and 6p electrons; and *A*_s_ is the isotropic Fermi contact term.

2 *A*_*x*_ = *A*_s_ – 4/7*P*_d_*a*^2^ – 2/5*P*_p_*b*^2^

3 *A*_*y*_ = *A*_s_ + 2/7*P*_d_*a*^2^ – 2/5*P*_p_*b*^2^

4 *A*_*z*_ = *A*_s_ + 2/7*P*_d_*a*^2^ + 4/5*P*_p_*b*^2^

Hence, *A*_*x*_ is expected to be the largest hyperfine component and *A*_*z*_ the smallest, as per the assignment in Table 3. Using *P*_d_ = +1.65 × 10^3^ and *P*_p_ = +1.21 × 10^3^ MHz,^41^,^48^ we get total Pt contribution to the SOMO (*a*^2^ + *b*^2^) of 8%, dominated by the 6p_*z*_ contribution.^41^ The larger isotropic hyperfine for **[5˙]^+^***cf.***[4˙]^+^** reflects an increase in the 5d_*yz*_ contribution from *ca.* 1 to 3%. Thus, the SOMO in both species has *ca.* 90% dioxolene character, which is consistent with DFT calculations (see below; Table 3).

**Fig. 6:**
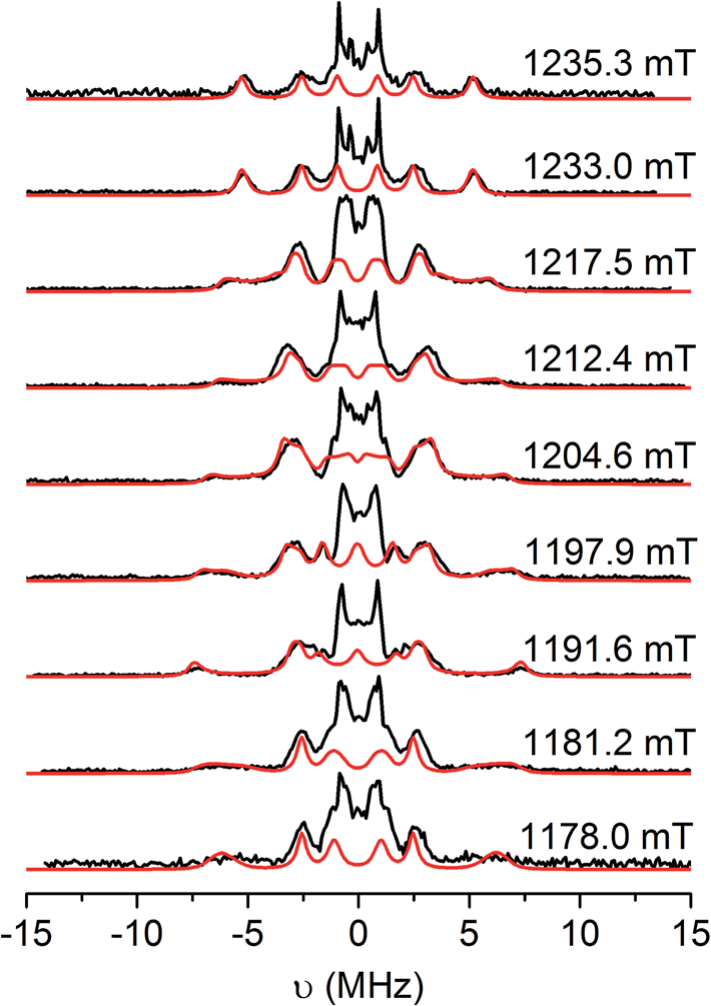
Q-band Davies ENDOR spectra of **[5˙]^+^** in CH_2_Cl_2_/THF solution at 20 K (black), and simulations (red) with the *g* and *a*^H^ parameters listed in Table 3. Static magnetic fields as shown, corresponding to the echo-detected field-swept spectrum in Fig. S14.† Experimental conditions: *ν*_mw_ = 33.710 GHz, *t*_RF_ = 16 μs, *t*_inv_ = 200 ns, *t*_π/2_ = 22 ns, *τ* = 440 ns.

Since there is no resolution of ligand hyperfine in the frozen solution spectra, orientation-selective Davies electron nuclear double resonance (ENDOR) and hyperfine sublevel correlation (HYSCORE) spectroscopies were undertaken to probe the spin distribution further. Detailed Q- and W-band ENDOR spectra have been reported for *para*-semiquinones,^54^ but we are unaware of any comparable study for the *ortho* equivalents. Q-band Davies ENDOR spectra for **[4˙]^+^** and **[5˙]^+^** and have nearly identical profiles in the ^1^H region (Fig. 6 and S15;† *ν*_*n*_(^1^H) = 51.33 MHz at 1200 mT), so we only discuss the latter in detail here.

The hyperfine couplings for the α-protons of the [DBsq˙]^–^ ligands (ring positions *H*4 and *H*6; Fig. S12†) arise from polarisation of C 2p_*z*_ spin density. The principal ^1^H hyperfine components are expected to be parallel to the C 2p_*z*_ (*a*_*z*_), along the C–H bond (*a*_*y*_), and orthogonal to these two axes (*a*_*x*_, with *a*_*x*_ > *a*_*z*_ > *a*_*y*_).^54^ However, spin density on neighbouring carbons can distort this pattern through rotation of the latter two axes about *z*. The C 2p_*z*_ orbitals are parallel to *g*_*z*_: this corresponds to the high-field extreme of the EPR spectrum. Hence, spectra at this field (1235.3 mT; Fig. 6, top) are single crystal-like: the largest splitting observed at this field is *a*_*z*_ = 10.6 MHz. This must be due to *H*4, because the *C*4,5 positions carry much greater spin density than *C*3,6.^47^ The maximum splitting observed is *ca.* 15 MHz at 1192 mT, that is, in the molecular *xy* plane. This corresponds to *a*_*x*_ of *H*4. Simulations (Fig. 5) give *a*_*x*,*y*,*z*_*H*4 = 15, 2.6, 10.6 MHz with *γ* = 60°, where *γ* is an Euler rotation angle about *z* relating the hyperfine (*a*_*xyz*_) and the molecular (*g*_*xyz*_) frames. The average *a* agrees well with the observed *A*_iso_ value from fluid solution.

A further ^1^H coupling of 5–7 MHz is observed at each orientation. This is too large to arise from the other α-proton *H*6 (very little spin density is carried by *C*6), while its near isotropic nature is also inconsistent with an α-proton. An alternative assignment might reflect hyperconjugation to the ^*t*^Bu group in the *C*5 position, oriented such that a proton folds back towards the ring π-system.^55^ These couplings are consistent with X-band HYSCORE spectra (Fig. S16†). Linear regression analysis of HYSCORE spectra gives evidence of a further, weak coupling with isotropic and anisotropic tensor components of 1.8 and 0.9 MHz, respectively, which we assign as *H*6. The ENDOR simulations are improved by making this rhombic (Table 3), but there is some uncertainty over these parameters. The isotropic *H*4 and *H*6 values of *a* = 9.4 and 1.8 MHz are a close match for those of [DBsq˙]^–^ itself in fluid solution (9.5 and 1.8 MHz).^47^ These parameters imply the spin density at *C*4,5 is *ca.* five times that at *C*3,6.

**Fig. 7:**
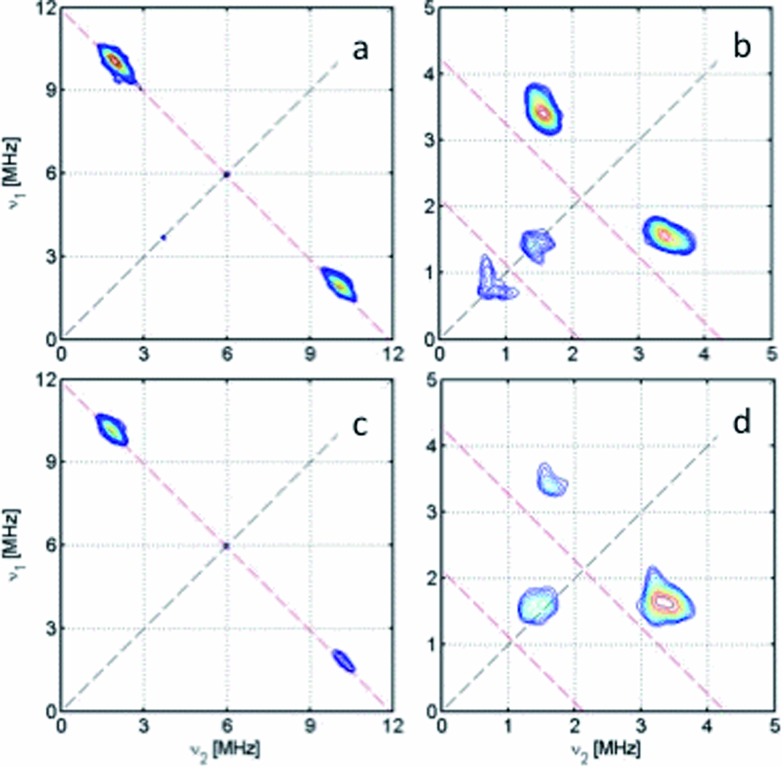
X-band HYSCORE spectra showing ^31^P coupling in (a) **[4˙]^+^** and (c) **[1˙]^+^**, and ^14^N coupling in (b) **[5˙]^+^** and (d) **[3˙]^+^**, measured at the maximum of the ESE-detected EPR spectra at 20 K. The anti-diagonals are in red, marking single and double quantum (for ^14^N) transitions frequencies.

X-band HYSCORE measurements on **[5˙]^+^** (Fig. 7b) also reveal coupling to the ^14^N atoms of the bipy co-ligand. The ^14^N coupling is near-isotropic, with analysis of the double quantum transitions giving *a*^N^ ≈ 1.0 MHz and quadrupole constant *K* = 0.36 MHz (*η* ≈ 0) (Fig. S17†). A near-isotropic ^31^P coupling is observed for **[4˙]^+^** in both HYSCORE (Fig. 7a) and Q-band ENDOR (Fig. S15†), giving *a*^P^ = 8.0 MHz, in agreement with the observed *a*_iso_. The very small anisotropic components (*ca.* 0.2 MHz) to the ^14^N and ^31^P hyperfine matrices put a limit of *ca.* 0.1% spin density at these positions.

These results were used to calibrate EPR data from the more elaborate tris-dioxolene systems derived from **1–3**.

### EPR spectroscopy of [{PtL}_3_(μ_3_-ctc˙)]^+^

The trinuclear radicals **[1˙]^+^** and **[3˙]^+^** were generated by *in situ* oxidation of **1** and **3** by 1 equiv. [Fc]PF_6_ at 220 K, inside a pre-cooled EPR tube. S-band spectra of chilled (210 K) fluid solutions of **[1˙]^+^** and **[3˙]^+^** show partial hyperfine resolution (Fig. S18 and S19†). The spectrum of **[3˙]^+^** (Fig. 4) shows a dominant hyperfine coupling to more than one ^195^Pt nucleus: simulation assuming coupling to three equivalent Pt nuclei with the appropriate statistical distribution gives *A*Ptiso of 31 MHz. This is very close to one third of the value for the related monometallic complex **[5˙]^+^** (Table 3) implying that, at this temperature, the radical is delocalised over the entire complex. The spectra of **[1˙]^+^** are consistent with this delocalisation: although the Pt satellites are not clearly resolved, simulations give an upper bound of *A*Ptiso < 15 MHz, much smaller than for the equivalent monomer **[4˙]^+^**. There is partial resolution of superhyperfine coupling with *a*_iso_ ≈ 2.7 MHz, which could not be unambiguously assigned. However, there is only one unique dioxolene α-proton environment in **[1˙]^+^** and **[3˙]^+^**, whose C-atoms (equivalent to the *C*3,6 sites in **[4˙]^+^** and **[5˙]^+^**) should carry little spin density. Hence, the superhyperfine in **[1˙]^+^** should be dominated by the six equivalent ^31^P atoms, and the observed *a*_iso_ is close to one-third the value found for **[4˙]^+^** (the spectra can be simulated with this model {Fig. S18†}; including ^195^Pt coupling in these simulations gives a best fit with *A*Ptiso = 12 MHz, but this is not well defined). In summary, fluid solution spectra of **[1˙]^+^** and **[3˙]^+^** are consistent with a delocalised radical model (Table 3).

In contrast, frozen solution X-band spectra of **[1˙]^+^** and **[3˙]^+^** at 150 K are very similar to their monometallic counterparts, with similar **g** and **A**^Pt^ matrices to **[4˙]^+^** and **[5˙]^+^**, respectively (Fig. 5 and Table 3). Hence, the radicals are localised in the frozen solutions of **[1˙]^+^** and **[3˙]^+^**, giving very similar electronic structures to the monomers **[4˙]^+^** and **[5˙]^+^**. This is confirmed by measurements of ligand hyperfines. ^1^H HYSCORE spectra for **[1˙]^+^** and **[3˙]^+^** at 20 K are similar, and show only a weak coupling with *a* ≈ 2 MHz, similar to that of the weaker coupled of the two α-protons (*H*6) in **[4˙]^+^** and **[5˙]^+^** (Fig. S20 and S21;† Table 3). Hence we assign this to the two α-protons (*H*3,6) of a single dioxolene ring of [ctc˙]^5–^. Convincingly, ^31^P and ^14^N HYSCORE give essentially identical couplings to those for **[4˙]^+^** and **[5˙]^+^** (Fig. 7). Therefore, electron hopping between the dioxolene groups of [ctc˙]^5–^ is suspended upon freezing of the matrix, localizing the electron spin on a single Pt-dioxolene fragment at these temperatures. A similar quenching of delocalisation on freezing was also reported in the dimetallic [{PtL}_2_(thea˙)]^+^ radical (Scheme 1).^15^

### Calculations

Geometry optimised structures and electronic properties of the free [DBsq˙]^–^ radical, **[1]^0/1+/2+/3+^**, **[3˙]^+^**, **[4˙]^+^** and **[5˙]^+^** were calculated by spin-unrestricted broken symmetry (BS) DFT calculations at the B3LYP-ZORA level. The ^*t*^Bu substituents on the bipy in **[3˙]^+^** and **[5˙]^+^**, and the phosphine Ph groups in **[1]^0/1+/2+/3+^** and **[4˙]^+^**, were replaced with H atoms.

The optimised structure of [DBsq˙]^–^ shows the alternating long-short aromatic C–C bond lengths expected for a semiquinone (Fig. S22†), with the average C–C distance intermediate between those typical for closed-shell catecholates and quinones.^56^,^57^ Calculated structures of the complexed ligands in **[4˙]^+^** and **[5˙]^+^** are similar (Table S4†), with slightly longer average C–O (*ca.* 1.30 Å) and shorter average C–C(ring) (1.41 Å) distances than free [DBsq˙]^–^ (1.26 and 1.43 Å, respectively).

**Fig. 8:**
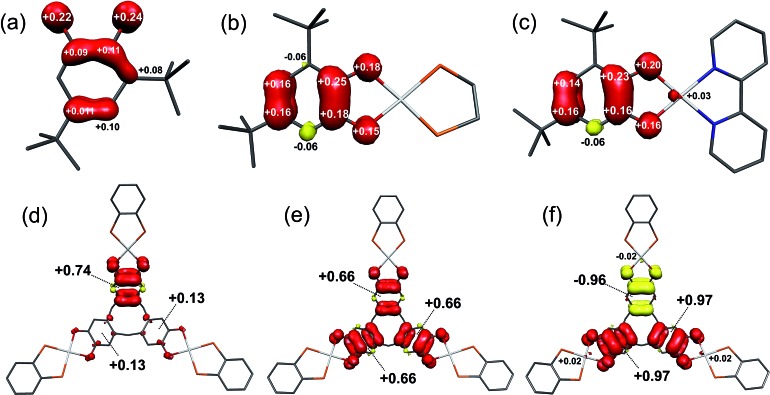
Mulliken spin populations for: (a) [DBsq˙]^–^; (b) **[4˙]^+^**; (c) **[5˙]^+^**; (d) **[1˙]^+^**; (e) **[1˙˙]^2+^**; (f) **[1˙˙˙]^3+^** (red: α-spin; yellow: β-spin).

Calculated Mulliken spin densities (Fig. 8a–c) confirm the ligand radical nature of the complexes. There is slightly greater localisation of the spin on the [DBsq˙]^–^ ring, and less on the O atoms, in the complexes than in the free radical. The total (5d_*yz*_ plus 6p_*z*_) Pt composition of the SOMO is calculated to be 7.4% in **[5˙]^+^** and 4.0% in **[4˙]^+^**. This agrees well with the 8% calculated from the EPR parameters, with the higher value for **[5˙]^+^** matching the increase in 5d_*yz*_ character from EPR (Table 3). Hence, the co-ligand has a small influence on the nature of the SOMO and we can speculate that the π-donor orbitals of the bipy in **[5˙]^+^** destabilise the 5d manifold such that they are energetically closer to the [DBsq˙]^–^ π orbitals.

The optimised structure of **1** is close to its crystallographic geometry (Table S7†).^27^ The bond lengths within the μ_3_-ctc ligand are accurately reproduced but the Pt–P and Pt–O distances are over- and under-estimated by 0.017 and 0.015 Å, respectively. The bowl structure of the cavitand is slightly more open in the computed structure as measured by the longer Pt···Pt separation (10.42 Å, *cf.* crystallographic 9.85 Å) and a larger dihedral angle between the dioxolene plane and the molecular three-fold axis. This stems from the absence of a solvent molecule trapped within the cavitand.^27^ The two highest occupied molecular orbitals (HOMOs) are the 1A_1_ and 2E combinations (*C*_3v_ molecular symmetry) of the three dioxolene π-orbitals (which are *b*_2_ in *C*_2v_ local symmetry; Fig. 10). The next filled MOs are the 1A_2_ (HOMO–2) and 1E (HOMO–3) combinations from the dioxolene π orbital with *a*_2_ symmetry. The lowest unoccupied orbitals (LUMOs) are combinations of dppb π* orbitals in groupings of 2A_1_ and 3E, and 2A_2_ and 4E. The 5E and 3A_2_ unoccupied orbitals are the lowest with large Pt character (∼34%), corresponding to combinations of the in-plane 5d_*xy*_ orbital, and are at considerably higher energy.

**Fig. 9:**
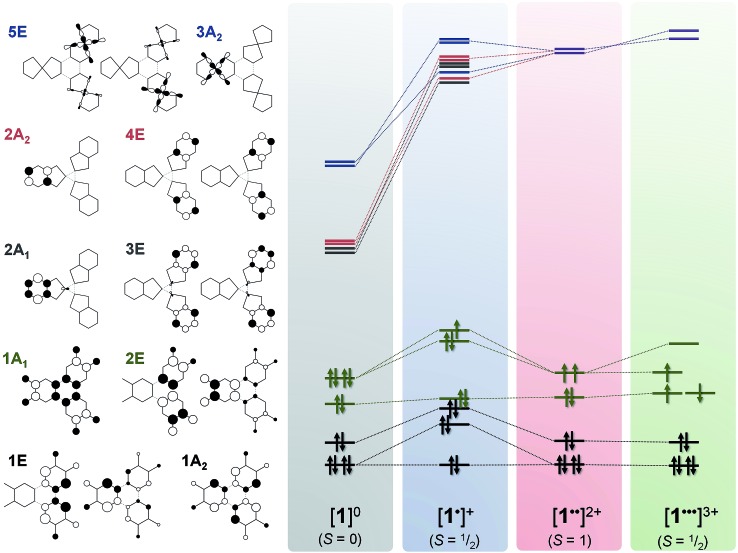
Qualitative MO scheme depicting the ordering of frontier orbitals for the **[1]^0/1+/2+/3+^** electron transfer series, derived from B3LYP-ZORA DFT calculations. The idealised orbitals shown left are derived from the calculation of **1** annotated with *C*_3v_ symmetry labels. The purple levels represent mixing of 2A_2_ and 4E (red) with 3A_2_ and 5E (blue). Detailed schemes and MO plots are in Fig. S25–S28†.

Removal of an electron from the ctc ligand to form **[1˙]^+^** does not affect the topology of the molecule, but lowers its symmetry to *C*_s_ (Table S7†). This is evident in the ctc ligand geometry, where one dioxolene unit has significantly shorter C–O bond lengths (1.337 Å) compared with the other two (1.347 Å). All degenerate E-type MOs in **1**, including the 2E HOMO, are split into A′ and A′′ components upon oxidation; this yields a 3A′ SOMO and 3A′′ HOMO–1 in **[1˙]^+^** (Fig. 9). The overall decrease in symmetry is relatively small, however, when considering the energy separation between previously degenerate pairs. The reduction in symmetry is more pronounced when the optimisation is performed in a highly polar solvent continuum. This effect is depicted in the Mulliken spin density plot for **[1˙]^+^**, where the majority of the spin (+0.74) is found on one arm of the complex (Fig. 8d). The corresponding plot for **[3˙]^+^** was derived from a gas-phase optimisation, and here the spin density is more evenly distributed across the three arms of the complex (Fig. S29†).

Interestingly, the formation of a semiquinone and the subsequent attenuation of the π-donor strength substantially stabilises the dppb π* (4A′ and 4A′′) and Pt 5d_*xy*_ (5A′′) orbitals (LUMO, LUMO+1 and LUMO+2, respectively; Fig. S26†) of one arm of **[1˙]^+^** relative to the other two, which remain near degenerate. The Pt content of the 4A′ (10.3%) and 5A′′ (40%) MOs is larger than for the corresponding orbitals in **1**. The MO manifold for **[3˙]^+^** is essentially identical, albeit with a smaller HOMO–LUMO energy gap reflecting the low-lying π* orbital of bipy (Fig. S31†).

The Mulliken spin density analysis for the *S* = 1/2 monocations of **1**, **3**, **4** and **5** can be used to validate the ^1^H hyperfine couplings measured by cw and pulsed EPR spectroscopy. The DFT-derived spin density on protons on the dioxolene unit are listed in Table 4, and show excellent agreement with the experimentally determined values according to the relationship *ρ*_H_ = *a*_iso_/1419.^58^ The accuracy of these DFT calculations was calibrated by comparison with experimental literature data from [DBsq˙]^–^.^47^

**Table 4:** Electronic absorption data for the complexes

	Proton^*a*^	*ρ*_calcd_	*ρ*_exptl_
[DBsq˙]^–^^*b*^	*H*4	–0.0060	–0.0068
	*H*6	–0.0017	–0.0013
**[4˙]^+^**	*H*4	–0.0062	–0.0067
	*H*6	–0.0010	–0.0013
**[5˙]^+^**	*H*4	–0.0054	–0.0068
	*H*6	–0.0012	–0.0013
**[1˙]^+^**	*H*3/*H*6^*c*^	–0.0008	–0.0013
**[3˙]^+^**	*H*3/*H*6^*c*^	–0.0009	–0.0017
[DBsq˙]^–^^*b*^	*H*4	–0.0060	–0.0068

^*a*^See ESI for atom numbering.

^*b*^Experimental data taken from ref. 46.

^*c*^Values averaged for these protons.

The dicationic complex, **[1˙˙]^2+^**, was investigated using a BS(1,1) method, and by spin unrestricted *S* = 0 (singlet) and *S* = 1 (triplet) calculations. The spin unrestricted singlet state is defined as removal of an electron from the SOMO of **[1˙]^+^**; the spin unrestricted triplet state (*S* = 1) is identical to the triplet state of the BS(1,1) calculation. The calculated spin triplet solution is 2 kcal mol^–1^ more stable than the BS singlet, and 11 kcal mol^–1^ more stable than the spin unrestricted singlet state. The exchange coupling constant, determined from the high-spin and BS energies together with the corresponding spin-expectation values S^2^,^59^,^60^ is calculated to be *J* = +731 cm^–1^.

The tris-dioxolene ligand in **[1˙˙]^2+^** is in its tetra-anionic form, [ctc˙˙]^4–^, involving an electron being removed from the 3A′′ MO of **[1˙]^+^** (Fig. 9). All three dioxolene units are metrically identical (Table S7†). The mean C–O (1.327 Å) and aromatic C–C (1.413 Å) distances are consistent with the average obtained for two semiquinones and one catecholate. The inter-metal separation is greater for **[1˙˙]^2+^** than **[1˙]^+^**, and stems from a subtle tilt of the PtP_2_O_2_ square plane away from the mean plane of the dioxolene unit. Examination of the frontier orbitals shows two near degenerate SOMOs whose composition resembles the corresponding MOs in **1**. The HOMO–1 is the totally symmetric 1A_1_ MO. Oxidation of the ligand has stabilised the Pt–L σ* orbitals (3A_2_ and 5E shown in blue, Fig. 9) such that they are energetically matched with the dppb π* (2A_2_ and E, in red) and mix in **[1˙˙]^2+^** (shown in purple). This dilutes their Pt content to ∼28%, down from ∼40% for **[1˙]^+^**. The subtle shift in energy of the Pt 5d orbitals leads to an increase in the metal content to 7% in HOMO–2 and HOMO–3 (1A_2_ and 1E, Fig. 9). The even distribution unpaired spins with +0.66 spins per arm of the cavitand underscores the three-fold symmetry of **[1˙˙]^2+^** (Fig. 6e).

Broken symmetry calculation of the electronic structure of **[1˙˙˙]^3+^** give the spin doublet to be only 0.4 kcal mol^–1^ more stable than the quartet: the *S* = 1/2 solution is shown in Fig. 7. The complex is again *C*_3v_ symmetric as demonstrated by the equivalent bond distances in the three {Pt(dioxolene)} units. The average C–O bond length is shortened to 1.308 Å, and the average aromatic C–C distance of 1.418 Å match those for the semiquinone ligands in the optimised structures of **[4˙]^+^** and **[5˙]^+^** (Table S4†). Thus, **[1˙˙˙]^3+^** possesses a tris-semiquinone [ctc˙˙˙]^3–^ ligand. The accumulation of unpaired spins on each of the dioxolene arms results in a subtle opening of the cavitand. The HOMO and HOMO–1 are the ctc-based 1A_2_ and 1E MOs. The 5d orbitals are further stabilised by the reduced ligand field strength of the oxidised ctc ligand where the LUMO+1 and LUMO+2 are now exclusively the Pt–L σ* possessing ∼42% metal character, and match the corresponding orbitals in **1** and **[1˙]^+^** (*vide supra*). Three SOMOs originating from the 1A_1_ and 2E MOs are generated: two α-spin (spin-up) and one β-spin (spin-down), where the latter is antiferromagnetically coupled to the corresponding α-spin SOMO of matching symmetry (Fig. 9 and S28†). The magnitude of the coupling is expressed by the overlap integral (*S*), which ranges from *S* = 0 for fully uncoupled (or perfectly orthogonal magnetic orbitals) to *S* = 1 for two electrons in a single MO. The value computed here of *S* = 0.19 is shows the antiferromagnetic coupling is relatively weak, consistent with the small exchange constant. This energy gap of 134 cm^–1^ (2*J*) represents the energy required to flip the β-spin to generate a spin quartet with three α-spin SOMOs, which would be populated at room temperature. The Mulliken spin density analysis shows one unpaired electron per dioxolene unit with 2% of the spin located on the Pt ions (Fig. 8f).

Notably, the calculated value of 2*J* = 134 cm^–1^ for **[1˙˙˙]^3+^** is comparable to measured values from other poly-dioxolene complexes at the all-sq level. These exhibit 1 ≤ |*J*| ≤ 209 cm^–1^, depending on the organic linker between the dioxolene rings and the dihedral angle between them.^14^,^20^,^21^ No measured *J* values from a mixed-valent cat/sq system analogous to **[1˙˙]^2+^** are available. However, an *ab initio* study of a different metal/organic tris-dioxolene system predicted that magnetic coupling in the mixed-valent cat/sq/sq diradical should be 10–100× stronger than the corresponding sq/sq/sq triradical.^61^ That agrees with the much larger superexchange constant calculated for **[1˙˙]^2+^** (+731 cm^–1^) than for **[1˙˙˙]^3+^**.

**Fig. 10:**
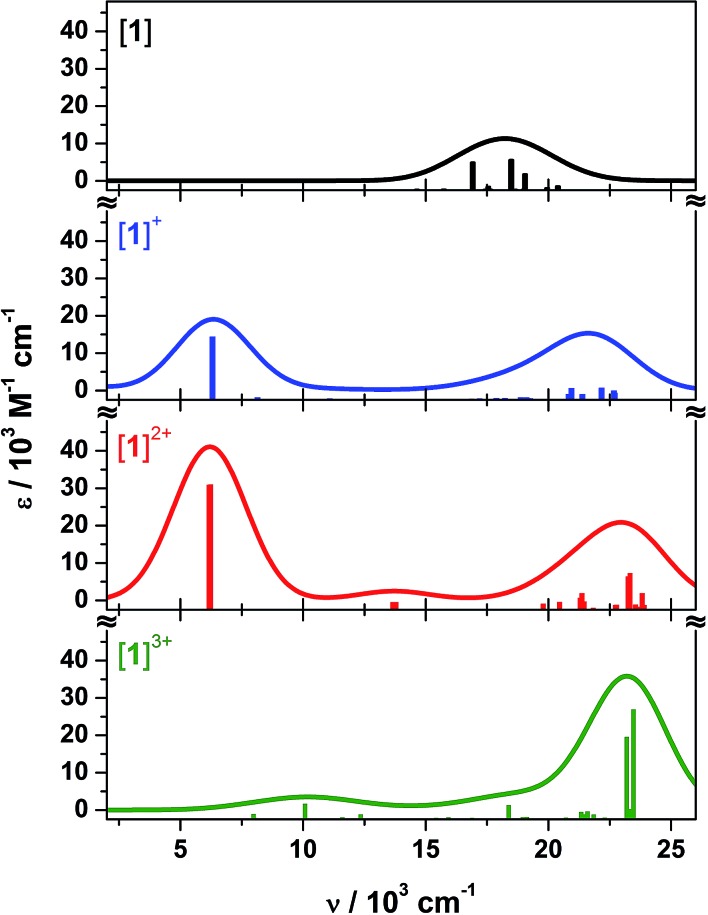
Calculated electronic absorption spectra for **1** (black), **[1˙]^+^** (blue), **[1˙˙]^2+^** (red), and **[1˙˙˙]^3+^** (green).

Time-dependent (TD) DF calculations were carried out for the four-membered series **[1]^0/1+/2+/3+^** in a dichloromethane solvent continuum. This method has reliably reproduced experimental data in analogous systems,^13^,^23^ the use of a solvent continuum being essential as corresponding gas phase calculations greatly underestimate the energy of IVCT bands.^62^ The position of the computed transitions matches the spectral profile, but their intensities are an order of magnitude larger than experiment (Fig. 10). Contributors to that discrepancy may include partial decomposition during electro-generation of the radical complexes (Fig. 3); the influence of rigidly encapsulated solvent within the molecular cavity;^27^ and the absence of vibrationally induced transition moments in the TD-DF treatment. The hallmark feature reproduced by TD-DF is the IVCT band in **[1˙]^+^** and **[1˙˙]^2+^**, whose computed energies at 6310 cm^–1^ (*cf.* 7900 cm^–1^ in **[1˙]^+^**) and 6204 cm^–1^ (*cf.* 7100 cm^–1^ in **[1˙˙]^2+^**) are well within the generally accepted error.^63^ This is defined as the 1A_1_ → 2E excitation in Fig. 9, or more explicitly the 2A′′ → 3A′′ transition for **[1˙]^+^** with its *C*_s_ molecular symmetry (Fig. S26†). The transition is computed to be twice as intense in the dication.

The weak band at 13 700 cm^–1^ in the calculated spectrum of **[1˙˙]^2+^** most likely corresponds to the 14 300 cm^–1^ peak observed experimentally (Fig. 3). It is defined as the 1E → 2E transition, where the low intensity stems from the poor overlap between these combinations of dioxolene π orbital (Fig. 9). The analogous transition in **[1˙]^+^** is not evident in the calculation, despite its very weak appearance in the experimental spectrum (Fig. 3). The broad transition envelope centered at ∼22 000 cm^–1^ in the mixed-valent species comprises excitations from the HOMO–8 and HOMO–10, and HOMO–4 and HOMO–5 (Fig. S32 and S33†). The former are a combination of d_*yz*_ (∼20%), dppb π and some tris-dioxolene character of the same phase as the singly occupied acceptor orbitals – 3A′′ in **[1˙]^+^** and 2E in **[1˙˙]^2+^** – while the latter are excitations to the SOMOs from MOs with *ca.* 57% d_*z*^2^_ character. These transitions dominate the electronic spectrum of **[1˙˙˙]^3+^** but in this case, the donor orbitals are have ∼45% mixed *d*_*yz*_ and d_*z*^2^_ character (Fig. S34†) reflecting the weaker field strength of the tris-semiquinone ligand. The spectrum of **[1˙˙˙]^3+^** lacks the intense IVCT band shown by **[1˙]^+^** and **[1˙˙]^2+^**, which is the hallmark for three dioxolene groups at the same oxidation level in the [Cr(dioxolene)_3_]^*z*^ (*z* = 3+, 2+, 1+, 0, 1–, 2–, 3–) series.^7^,^64^ A weak band at 10 080 cm^–1^ is assigned as the SOMO → LUMO excitation whose intensity is proportional to the poor overlap between this collection of near orthogonal orbitals (Fig. S28†).

## Conclusions

The [ctc]^6–/5–/4–/3–^ redox series, which was first noted seventeen years ago in a series of platinum(ii) complexes (Scheme 1),^27^ has been characterised by spectroscopic (UV/vis/NIR, cw and pulsed EPR) and computational methods. The ctc radical complexes are poorly stable above 270 K, which has thus far precluded their isolation as pure compounds. Their instability may stem from the poor steric protection afforded by the exposed CH_2_ groups in the ctc macrocycle.^65^ None-the-less, with the help of data from monometallic model compounds, the mixed valent character of coordinated [ctc˙]^5–^ radicals has been determined in detail.

The ligand radicals **[1˙]^+^** and **[3˙]^+^** have class II character, with more localised SOMOs than radicals derived from fully conjugated triscat,^23^ or the comparable bis-dioxolene thea (Scheme 1).^15^ In the latter case, this will reflect differing degrees of through-space overlap between neighbouring dioxolene π-systems, which are linked by one methylene bridge in ctc and two methylene bridges in thea. Electron hopping around the [ctc˙]^5–^ macrocycle is rapid in fluid solution, but slows below the EPR timescale when the solution is frozen.^15^ While the metal/ligand character of the SOMO in **[1˙]^+^** and **[3˙]^+^** is essentially the same (Table 3), they exhibit differing temperature dependence of unpaired spin delocalisation. That might explain the different trends in the oxidation potentials and IVCT linewidths of **[1]^*n*+^** and **[3]^*n*+^** (*n* = 1–3), as their dioxolene rings are sequentially oxidised.

Intramolecular mixed-valency between three or more redox centers is well-known,^9^ with examples based on redox sites that are well-separated (by a 1,3,5-triphenylene scaffold, for example); linked by fully conjugated spacers (like triscat (Scheme 1) or hexaazatriphenylene);^9^,^23^,^30^ or, bound by a centrally coordinated metal ion.^7^,^43^,^66^ The mixed-valent macrocycle [ctc˙]^5–^ is distinct from these scenarios, in having three dioxolene redox sites that interaction *via* through-space π–π overlap.^31^ As such, it is the first mixed-valent radical derived from a tri-cyclophane-type precursor to be spectroscopically characterised.^31^ Our current work aims to incorporate additional steric protection onto the ctc framework,^67^ to produce mixed-valent radicals that are stable enough for use in frameworks and supramolecular architectures.^24^,^32^

## Experimental

Cyclotricatechylene (H_6_ctc),^35^ [PtCl_2_(L)] (L = dppb, dppe and ^*t*^Bu_2_bipy),^68^ [{Pt(dppb)}_3_(μ_3_-ctc)] (**1**),^27^ [Pt(dppe)(DBcat)] (**4**) and [Pt(^*t*^Bu_2_bipy)(DBcat)] (**5**)^13^ were all prepared by the literature procedures. Other reagents were used as supplied. Experimental data and procedures for the crystal structure determinations, electrochemical and spectroelectrochemical measurements, cw and pulsed EPR experiments and the computational study are given in the ESI.†

### Synthesis of [{Pt(dppe)}_3_(μ_3_-ctc)] (**2**)

A solution of H_6_ctc (0.037 g, 0.1 mmol) and [PtCl_2_(dppe)] (0.24 g, 0.37 mmol) in deoxygenated dimethylacetamide (25 cm^3^) was stirred for 30 min at room temperature. A suspension of K_2_CO_3_ (0.102 g, 0.73 mmol) in dry, deoxygenated methanol (10 cm^3^) was then added, and the mixture was stirred at 70 °C for 18 h resulting in the formation of a deep yellow colouration. After cooling, excess dry, deoxygenated diethyl ether was added to precipitate the crude yellow product which was isolated under a nitrogen atmosphere. Recrystallisation from dry, deoxygenated dichloromethane/acetone yielded the product as a deep yellow powder. Yield: 0.035 g (16%). Found C, 53.2; H, 4.00%. Calcd for C_99_H_92_O_10_P_6_Pt_3_ C, 53.7; H, 4.19%. ES-MS *m*/*z* 629.1 (53, [Pt(dppe)(OH_2_)_2_]^+^), 689.1 (23, [Pt(dppe)(OH_2_)(dmso)]^+^), 706.1 (27, [NaPt(dppe)(O_2_CH)_2_]^+^), 1093.1 (16, [Na_2_Pt_3_(dppe)_3_(ctc)]^2+^), 1101.1 (26, [NaKPt_3_(dppe)_3_(ctc)]^2+^), 1109.1 (38, [K_2_Pt_3_(dppe)_3_(ctc)]^2+^), 1131.7 (23, [Na_2_Pt_3_(dppe)_3_(ctc)(dmso)]^2+^), 1140.2 (33, [NaKPt_3_(dppe)_3_(ctc)(dmso)]^2+^), 1146.2 (27, [K_2_Pt_3_(dppe)_3_(ctc)(dmso)]^2+^), 2163.3 (15, [NaPt_3_(dppe)_3_(ctc)]^+^), 2179.4 (100, [KPt_3_(dppe)_3_(ctc)]^+^). ^1^H NMR ({CD_3_}_2_SO) *δ* 3.60 (m, 12H, dppe C_2_*H*_4_), 4.54 (br, 3H, ctc C*H*_2_), 6.53 (s, 6H, ctc C*H*), 7.51 and 8.06 (both m, 60H, dppe C_6_*H*_5_) – an additional diastereotopic ctc C*H*_2_ resonance expected near 3.3 ppm was obscured under the dmso water peak. ^31^P NMR ({CD_3_}_2_SO) *δ* 30.8 (*J*_P–Pt_ 3350 Hz).

### Synthesis of [{Pt(^*t*^Bu_2_bipy)}_3_(μ_3_-ctc)] (**3**)

Method as for **2**, using [PtCl_2_(^*t*^Bu_2_bipy)] (0.20 g, 0.37 mmol). After addition of K_2_CO_3_ the mixture was heated to 40 °C for 18 h. After cooling, the blue solution was diluted with dry, deoxygenated 1 : 1 diethyl ether/pentane and the blue precipitate collected. The product could be purified by either recrystallisation from dichloromethane/acetone at –20 °C or silica column chromatography (98.5 : 1.5 CH_2_Cl_2_/MeOH eluent) to yield the product as a blue powder. Yield: 0.049 g (28%). Found C, 50.1; H, 5.15; N, 4.3%. Calcd for C_75_H_90_N_6_O_9_Pt_3_ C, 49.9; H, 5.03; N, 4.7%. ES-MS (MeCN) *m*/*z* 269.2 (48, [H(^*t*^Bu_2_bipy)]^+^), 522.1 (26, [Pt(^*t*^Bu_2_bipy)(NCCH_3_)(OH_2_)]^+^), 549.1 (26, [Pt(^*t*^Bu_2_bipy)(NCCH_3_)(O_2_CH)]^+^), 572.2 (39, [NaPt(^*t*^Bu_2_bipy)(NCCH_3_)(O_2_CH)]^+^), 584.2 (100, [H_3_Pt_3_-(^*t*^Bu_2_bipy)_3_(ctc)]^3+^), 597.2 (20, [H_2_KPt_3_(^*t*^Bu_2_bipy)_3_(ctc)]^3+^), 609.7 (26, [HK_2_Pt_3_(^*t*^Bu_2_bipy)_3_(ctc)]^3+^), 617.3 (19, [NaK_2_Pt_3_(^*t*^Bu_2_bipy)_3_(ctc)]^3+^), 645.4 (24, [H_2_Pt_2_(^*t*^Bu_2_bipy)_2_(ctcH_2_)]^2+^), 663.4 (23, [HKPt_2_(^*t*^Bu_2_bipy)_2_(ctcH_2_)]^2+^), 875.8 (30, [H_2_Pt_3_(^*t*^Bu_2_bipy)_3_(ctc)]^2+^), 887.3 (23, [HNaPt_3_(^*t*^Bu_2_bipy)_3_-(ctc)]^2+^), 895.3 (23, [HKPt_3_(^*t*^Bu_2_bipy)_3_(ctc)]^2+^), 1167.4 (27, [NaPt_2_(^*t*^Bu_2_bipy)(NCMe)_2_(ctcH)(O_2_CH)]^+^), 1288.4 (9, [Pt_2_(^*t*^Bu_2_bipy)_2_(ctcH_2_)]^+^), 1751.5 (49, [HPt_3_(^*t*^Bu_2_bipy)_3_(ctc)]^+^), 1772.5 (28, [NaPt_3_(^*t*^Bu_2_bipy)_3_(ctc)]^+^), 1789.5 (28, [KPt_3_(^*t*^Bu_2_bipy)_3_(ctc)]^+^). ^1^H NMR (CDCl_3_) *δ* 1.28 (s, 54H, C{C*H*_3_}_3_), 3.30 and 4.51 (both d, 14.0 Hz, 3H, ctc C*H*_2_), 6.67 (s, 6H, ctc C*H*), 7.35 (dd, 1.9 and 6.4 Hz, 6H, Py *H*^5^), 7.84 (d, 1.9 Hz, 6H, Py *H*^3^), 8.77 (d, 6.4 Hz, 6H, Py *H*^6^).

## Supplementary Material

Supplementary information

Crystal structure data
