# Supplementary material for: Platinum(ii) complexes of mixed-valent radicals derived from cyclotricatechylene, a macrocyclic tris-dioxolene
Source: Chem Sci. 2015 Aug 20;6(12):6935–48. doi: 10.1039/c5sc02776d (PMC5951140; doi:10.1039/c5sc02776d)
Supplement: Supplementary file 1 [file SC-006-C5SC02776D-s001.pdf]

Electronic Supplementary Material (ESI) for Chemical Science.
This journal is © The Royal Society of Chemistry 2015

Platinum(II) Complexes of Mixed-Valent Radicals Derived from Cyclotricatechylene, a Macrocyclic Tris-Dioxolene

Jonathan J. Loughrey,[a] Nathan J. Patmore,[b,c] Amgalanbaatar Baldansuren,[d] Alistair J. Fielding,[d] Eric J. L. McInnes,[d] Michaele J. Hardie,[a] Stephen Sproules,*[e] and Malcolm A. Halcrow*[a]

[a]*School of Chemistry, University of Leeds, Woodhouse Lane, Leeds LS2 9JT, United Kingdom*

[b]*Department of Chemistry, University of Sheffield, Brook Hill, Sheffield S3 7HF, United Kingdom*

[c]*Current address: Department of Chemical and Biological Sciences, University of Huddersfield, Huddersfield HD1 3DH, United Kingdom*

[d]*School of Chemistry and Photon Science Institute, University of Manchester, Oxford Road, Manchester M13 9PL, United Kingdom*

[e]*WestCHEM, School of Chemistry, University of Glasgow, Glasgow G12 8QQ, United Kingdom*

E-mail: stephen.sproules@glasgow.ac.uk
m.a.halcrow@leeds.ac.uk

Supporting Information

Experimental details for the crystal structure determinations

Table S1. Experimental data for the crystal structure determinations in this work.

Experimental details for the electrochemical, spectroscopic and computational studies

Figure S1 Electrospray mass spectra of **2** and **3**.

Figure S2 ^{1}H NMR spectra of **2** in $\{CD_3\}_2SO$ at 298 K, under an N_2 atmosphere and in air.

Figure S3 ^{1}H NMR spectrum of **3** in $CDCl_3$ at 298 K.

Figure S4 *X*-band EPR spectrum of freshly prepared **3** in aerobic $CHCl_3$ solution at 150 K.

Figure S5 View of the $[\{Pt(dppe)\}_3(ctc)]$ half-molecule in **2**·H_2O·8dma.

Figure S6 View of the $[\{Pt(dppe)\}_3(ctc)]$ molecule in **2**·$2H_2O$·1.3dma·0.5MeOH.

Figure S7 View of the disorder of the complex in **2**·H_2O·8dma across a crystallographic mirror plane.

Figure S8 Alternative view of **2**·$2H_2O$·1.3dma·0.5MeOH, showing inclusion of dma into the cavity of the complex.

Table S2 Selected bond distances and angles for the two solvates of **2**.

Figure S9 Titration of the cat→sq oxidation of **5** and **6** with $[Fc]PF_6$, monitored by UV/vis/NIR spectroscopy.

Figure S10 Experimental and simulated EPR spectra of **[4•]**$^+$ in CH_2Cl_2/THF solution

Figure S11 Experimental and simulated EPR spectra of **[5•]**$^+$ in CH_2Cl_2/THF solution

Figure S12 Axis and numbering systems for **[4•]**$^+$ and **[5•]**$^+$

Figure S13 Q-band ESE-detected EPR spectrum of **[4•]**$^+$

Figure S14 Q-band ESE-detected EPR spectrum of **[5•]**$^+$

Figure S15 Orientation selected Davies ENDOR spectra of **[4•]**$^+$

Figure S16 Experimental and simulated X-band ^{1}H HYSCORE spectrum of **[5•]**$^+$

Figure S17 Experimental and simulated X-band ^{14}N HYSCORE spectra of **[5•]**$^+$

Figure S18 Experimental and simulated EPR spectra of **[1•]**$^+$ in CH_2Cl_2/THF solution

Figure S19 Experimental and simulated EPR spectra of **[3•]**$^+$ in CH_2Cl_2/THF solution

Figure S20 Experimental and simulated X-band ^{1}H HYSCORE spectrum of **[1•]**$^+$

Figure S21 Experimental and simulated X-band ^{1}H HYSCORE spectrum of **[3•]**$^+$

Table S3 Geometry Optimised Coordinates for [DBsq•]$^-$.

Figure S22 Geometry optimised structure and isosurface plot of the ground state molecular orbital for [DBsq•]$^-$

Table S4 Comparison of experimental metrics in **4** and **5** with calculated metrics for the corresponding S = ½ species **[4•]**$^+$ and **[5•]**$^+$.

Table S5 Geometry Optimised Coordinates for **[4•]**$^+$.

Table S6 Geometry Optimised Coordinates for **[5•]**$^+$.

Figure S23 Isosurface plot of the SOMO in **[4•]**$^+$.

Figure S24 Isosurface plot of the SOMO in **[5•]**$^+$.

Table S7 Comparison of the experimental and calculated mean bond distances and angles in the different oxidation levels of **[1]**^{z}.

Table S8 Geometry optimised coordinates for **1**.

Figure S25 MO energy level scheme of frontier Kohn-Sham orbitals for **1**.

Table S9 Geometry optimised coordinates for **[1•]**$^+$.

Figure S26 MO energy level scheme of frontier Kohn-Sham orbitals for **[1•]**$^+$.

Table S10 Geometry optimised coordinates for **[1••]**$^{2+}$.

Figure S27 MO energy level scheme of frontier Kohn-Sham orbitals for **[1$^{••}$]$^{2+}$**.

Table S11 Geometry optimised coordinates for **[1$^{•••}$]$^{3+}$**.

Figure S28 MO energy level scheme of frontier Kohn-Sham orbitals for **[1$^{•••}$]$^{3+}$**.

Table S12 Averaged bond distances and angles in geometry optimised **[3$^{•}$]$^{+}$**

Table S13 Geometry optimised coordinates for **[3$^{•}$]$^{+}$**.

Figure S29 Mulliken spin density map for **[3]$^{+}$**.

Figure S30 Isosurface plot of the ground state molecular orbital of **[3$^{•}$]$^{+}$**.

Figure S31 MO energy level scheme of frontier Kohn-Sham orbitals for **[3$^{•}$]$^{+}$**.

Figure S32 Orbitals involved in electronic transitions on the β manifold of **[1$^{•}$]$^{+}$**.

Figure S33 Orbitals involved in electronic transitions on the β manifold of **[1$^{••}$]$^{2+}$**.

Figure S34 Orbitals involved in electronic transitions on the β manifold of **[1$^{•••}$]$^{3+}$**.

Single crystal *X*-ray structure determinations

Slow diffusion of methanol into a solution of **2** in dma under an N_2 atmosphere yielded a mixture of two different solvate crystals. Both these were analysed, using a Rigaku Saturn CCD diffractometer in station I19 at the UK Diamond Light Source synchrotron (λ = 0.6889 Å). Experimental details of the structure determinations in this study are given in Table S1. All the structures were solved by direct methods (*SHELXS97*[1]), and developed by full least-squares refinement on F^2 (*SHELXL97*[1]). Crystallographic figures were prepared using *XSEED*.[2]

Table S1. Experimental data for the crystal structure determinations in this work.

	**2**·H_2O·8dma	**2**·2H_2O·1.3dma·0.5MeOH
empirical formula	$C_{131}H_{158}N_8$-$O_{15}P_6Pt_3$	$C_{104.70}H_{101.78}N_{1.30}O_{9.84}P_6Pt_3$
Mr / g mol^{-1}	2855.74	2306.78
crystal class	hexagonal	monoclinic
space group	$P6_3/m$	$P2_1$
a / Å	23.8013(6)	14.860(3)
b / Å	–	23.898(5)
c / Å	13.6022(5)	15.103(3)
β / °	–	98.87(3)
V / Å^3	6673.3(3)	5299.1(18)
Z	2	2
ρ_{calc} / g cm^{-1}	1.421	1.446
μ / mm^{-1}	3.270	4.094
T / K	173(2)	173(2)
θ range / °	1.98–27.49	1.61–27.70
measured reflections	66070	51623
independent reflections	5308	20525
R_{int}	0.083	0.039
R_1, $I > 2\sigma(I)$	0.080	0.047
wR_2, all data	0.190	0.137
goodness of fit, F^2	1.203	1.041
Flack parameter	–	0.009(7)
$\Delta\rho_{min/max}$ / eÅ^{-3}	–1.30/1.44	–1.56/1.74
CCDC	1037214	1037215

The entire contents of the asymmetric unit of **2**·H_2O·8dma are disordered, across a crystallographic mirror plane. Metal ion Pt(1) lies on this mirror plane, but every other atom in the complex and the resolved solvent residues is half-occupied and on a general crystallographic site. The asymmetric unit contains ⅓ of the complex half-molecule, spanning a C_3 axis, and two dma half-molecules which lie near the same mirror plane as the complex. The four half-occupied phenyl groups in the asymmetric unit were refined as rigid hexagons, but no other restraints were applied to the model. There is no resolved organic solvent in the cavity of the complex. However, a Fourier peak lying on the same C_3 axis as the complex molecule, [⅓, ⅔, z], refined reasonably as a half-molecule of water. The contents of the cavity of the complex were further defined by a *SQUEEZE* analysis of the final model.[35] This identified only small cavities totalling 490 Å^3 per unit cell, which is 7% of the cell volume V, containing 192 electrons. That is equivalent to a void volume of 245 Å^3 and 96 electrons per molecule, which corresponds perfectly to two molecules of dma (48 electrons each). Therefore, the cavities of the complex are assumed to contain two additional, unresolved dma molecules, and this formula was used for the density and F(000) calculations. All non-H atoms in the model were refined anisotropically, while H atoms were placed in calculated positions and refined using a riding model.

The asymmetric unit of **2**·2H_2O·1.3dma·0.5MeOH contains one molecule of the complex, two disordered solvent sites and some additional Fourier peaks that were also modelled as partial solvent environments. Six phenyl groups in the complex were obviously disordered, and refined over two sites as rigid hexagons. Two other phenyl groups in the model also deviate significantly from planarity which indicates disorder, but attempts to resolve that were unsuccessful in those cases. The dma molecule in the cavity of the complex was refined over two sites, with occupancies 0.5 and 0.3. Another solvent site was modelled as a mixture of dma and water, each with half-occupancy. A methanol molecule was also clearly resolved in the model, which was refined as half-occupied. Lastly, three other Fourier peaks that were not bonded to any other atom were included as partial water sites. Fixed interatomic distance restraints were applied to all the partial dma molecules. All wholly occupied non-H atoms were refined anisotropically, while C-bound H atoms were placed in calculated positions and refined using a riding model.

EPR Spectroscopy

Multifrequency EPR measurements were carried out at the EPSRC National UK EPR Facility and Service in the Photon Science Institute at The University of Manchester. Samples were prepared by oxidation of the neutral complex by ferrocenium hexafluorophosphate in CH_2Cl_2 solution at –78 °C with a concentration ranging 3-5 mM. A small amount of THF was added to the reaction mixture before samples were loaded into the appropriately sised quartz tubes. S- and X-band fluid solution continuous wave (cw) spectra were collected using a Bruker EMX Micro spectrometer. Frozen solution X- and Q-band cw spectra were measured using a Bruker EMX spectrometer. Simulations of cw data were performed using Bruker's *Xsophe* software package.[3]

Pulsed X- and Q-band EPR measurements were performed on a Bruker *ELEXSYS* E580 spectrometer at 20 K. The standard dielectric ring Bruker EPR cavities (ER 4118S-MS5), (ER4118X-MD5) and (EN4118X-MD4) were used, which were equipped with an Oxford CF 935 helium flow cryostat. X-band ESE-detected EPR spectra were measured with a 16 – 200 – 32 ns Hahn echo pulse sequence; Q-band utilised a 22 – 400 – 44 ns sequence. Davies ENDOR were performed using the pulse sequence $\pi_{\text{inv}} -$ RF $- \pi/2 - \tau - \pi - \tau -$ echo.[4] RF pulses of 16 μs were generated by the Bruker "DICE" system and amplified by a 60 dB gain ENI A-500 RF amplifier. The three-pulse ESEEM (stimulated echo) experiments were performed using the pulse sequence $\pi/2 - \tau - \pi/2 - T - \pi/2 - \tau -$ echo as a function of T at different, fixed time τ.[5] The 2D *ESEEM* spectra, so-called hyperfine sublevel correlation (*HYSCOR*E) spectra,[6] were recorded employing the sequence $\pi/2 - \tau - \pi/2 - t_1 - \pi - t_2 - \pi/2 - \tau -$ echo with mw pulses of length $t_{\pi/2}$ = 16 ns and t_π = 32 ns, starting times $t_{1,2}$ = 500/300 ns, and time increments $\Delta t_{1,2}$ = 20 ns. The intensity of the inverted echo following the fourth pulse is measured with t_2 and t_1 varied and constant τ (of 300 ns). Unwanted features from the experimental electron spin echo envelopes were removed by using a four-step phase cycle.[7,8] In both dimensions 256 data points were collected. The relaxation decay was subtracted using baseline corrections (fitting by polynomials of 3-6 degree) in both time domains, subsequently applying apodisation (Hamming window) and zero-filling to 1024 data points in both dimensions. After 2D fast Fourier transformation absolute-value spectra were obtained. Initial analysis of the cross-ridges in (n_1^2) versus (n_2^2) allows in many cases for simultaneous determination of the isotropic and anisotropic components of the hyperfine matrix.[9-11] Simulations of EPR, ENDOR and HYSCORE data were performed using *Easyspin*,[12,13] with the following spin-Hamiltonian (Eq. 1):

$$\hat{H} = \beta\vec{B}\cdot\mathbf{g}\cdot\hat{S} + \sum_{i=1}^{n}\left(-\beta_n g_n^i\vec{B}\cdot\hat{I}_i + \hat{S}\cdot\mathbf{A}\cdot\hat{I}_i + \hat{I}_i\cdot\mathbf{P}\cdot\hat{I}_i\right) \quad (1)$$

where β is the Bohr magneton, β_n is the nuclear magneton, g^i_n is the g-factor of the ith nucleus, and g represents the electronic **g**-matrix. The first and second terms in the expression correspond to the electron and nuclear Zeeman interactions with the external magnetic field; the third term describes the hyperfine interaction defined by matrix **A**. The last term describes the quadrupole coupling defined by tensor **P**, which is traceless. The representation in its principal axes system is defined by Eq. 2.

$$\left[P_x, P_y, P_z\right] = \frac{e^2qQ}{4I(2I-1)h}\left[-(1-\eta), -(1+\eta), 2\right] \quad (2)$$

Here, we will use the two usual parameters to characterise the ^{14}N quadrupole coupling constants: $K = e^2qQ/4h$ and $\eta = (P_x - P_y)/P_z$. In all calculations, the electron Zeeman interaction was assumed to be the dominant term. All other interactions were treated as a perturbation. The orientation of the **A**-matrix and **P**-tensor was defined with respect to the principal axes of the electronic **g**-matrix.

Calculations

Geometry optimisations of all complexes were performed at the BP86 level using *Gaussian 09*[14] for energy calculations and the *DL-FIND* algorithm[15] implemented within the *ChemShell* package[16,17] for optimisation. The geometries of all complexes were fully optimised by spin-unrestricted DFT method with dichloromethane as solvent. The all-electron basis sets were those reported by the Ahlrichs group.[18,19] Triple-ξ-quality basis sets with one set of polarisation functions (def2-TZVP) were used for all atoms. The stability of all solutions was checked by performing frequency calculations: No negative frequencies were observed. Electronic properties were calculated on the optimised coordinates at the B3LYP[20,21] level of theory using *ORCA*.[22] The conductor like screening model (*COSMO*) was used for all calculations (except **[3•]**$^+$),[23] and *RIJCOSX* approximation[24] combined with appropriate Ahlrichs auxiliary basis sets were routinely employed to speed up the calculations.[25,26] Relativistic effects were accounted for using the zero order regular approximation (*ZORA*),[27] and enhanced integration accuracy was used for platinum (*SPECIALGRIDINTACC 12*). The exchange coupling constants *J* were calculated on broken-symmetry[28-32] geometries using Eq. 3,[33,34] and assuming the spin-Hamiltonian Eq. 4 is valid.

$$J = \frac{E_{HS} - E_{BS}}{\langle \hat{S}^2 \rangle_{HS} - \langle \hat{S}^2 \rangle_{BS}} \qquad (3)$$

$$\hat{H} = -2J\hat{S}_A \cdot \hat{S}_B \qquad (4)$$

TD-DFT calculations were performed using the *B3LYP* functional with dichloromethane as a solvent. The first 40 states were calculated, whereas the maximum dimension of the expansion space in the Davidson procedure (MAXDIM) was set to 400. The full width at half maximum (FWHM) was set to 3500 cm^{-1}. Molecular orbitals and spin density maps were visualised via the program *Moleke*l.[35]

Other measurements

Elemental microanalyses were performed by the University of Leeds School of Chemistry microanalytical service. Electrospray mass spectra (MS) were obtained on a Bruker MicroTOF spectrometer, from MeCN feed solutions. All mass peaks have the correct isotopic distributions for the proposed assignments. Alkali metal cations and formate anions in the molecular ion assignments originate from calibrants in the spectrometer feed solutions. NMR spectra were run using a Bruker Avance 500 spectrometer operating at 500.1 MHz (^{1}H) or 125.6 MHz (^{31}P). UV/vis/NIR spectra for redox titrations were run on a Perkin Elmer Lambda900 spectrophotometer using 1 cm quartz cells.

Electrochemical measurements were carried out using an Autolab PGSTAT20 voltammetric analyser, under an argon atmosphere, in predried CH_2Cl_2 containing 0.5 M [^{n}Bu$_4$N]BF$_4$ as supporting electrolyte. Voltammetry experiments used a Pt disk working electrode, a Pt rod counter electrode and an Ag/AgCl reference electrode. All potentials quoted are referenced to internal ferrocene and were obtained at a scan rate (v) of 100 mV s^{-1}. The Fc$^{+/0}$ couple under these conditions was observed at $+0.42 \leq E_{1/2} \leq 0.48$ V vs. Ag/AgCl. UV/vis/NIR spectroelectrochemistry experiments were conducted using a Cary 5000 spectrophotometer, fitted with an optically transparent thin layer electrode (OTTLE) cell. The working electrode was platinum gauze in the path of the beam, the counter electrode was platinum wire, and silver wire was used as the reference electrode. The electrolysis was controlled by an EG&G 273A potentiostat/galvanostat.

References

1 G. M. Sheldrick, *Acta Cryst., Sect. A*, 2008, **64**, 112.
2 L. J. Barbour, *J. Supramol. Chem.*, 2001, **1**, 189.
3 G. R. Hanson, K. E. Gates, C. J. Noble, M. Griffin, A. Mitchell and S. Benson, *J. Inorg. Biochem.*, 2004, **98**, 903.
4 E. R. Davies, *Phys. Lett. A*, 1974, **47**, 1.
5 S. A. Dikanov and Y. D. Tsevtkov, *Electron Spin Echo Envelope Modulation (ESEEM) Spectroscopy*, CRC Press, Boca Raton, USA, 1992.
6 P. Höfer, A. Grupp, M. Nebenführ and M. Mehring, *Chem. Phys. Lett.*, 1986, **132**, 279.
7 C. Gemperle, G. Aebli, A. Schweiger and R. R. Ernst, *J. Magn. Reson.*, 1990, **88**, 241.

8 A. Schweiger and G. Jeschke, *Principles of Pulse Electron Paramagnetic Resonance*, Oxford University Press, New York, USA, 2001.
9 S. A. Dikanov and M. K. Bowman, *J. Magn. Reson., Ser. A*, 1995, **116**, 125.
10 S. A. Dikanov, A. M. Tyryshkin and M. K. Bowman, *J. Magn. Reson.*, 2000, **144**, 228.
11 M. T. Lin, A. Baldansuren, R. Hart, R. I. Samoilova, K. V. Narasimhulu, L. L. Yap, S. K. Choi, P. J. O'Malley, R. B. Gennis and S. A. Dikanov, *Biochemistry*, 2012, **51**, 3827.
12 S. Stoll and A. Schweiger, *J. Magn. Reson.*, 2006, **178**, 42.
13 S. Stoll and R. D. Britt, *Phys. Chem. Chem. Phys.*, 2009, **11**, 6614.
14 M. J. Frisch, G. W. Trucks, H. B. Schlegel, G. E. Scuseria, M. A. Robb, J. R. Cheeseman, G. Scalmani, V. Barone, B. Mennucci, G. A. Petersson, H. Nakatsuji, M. Caricato, X. Li, H. P. Hratchian, A. F. Izmaylov, J. Bloino, G. Zheng, J. L. Sonnenberg, M. Hada, M. Ehara, K. Toyota, R. Fukuda, J. Hasegawa, M. Ishida, T. Nakajima, Y. Honda, O. Kitao, H. Nakai, T. Vreven, J. A. Montgomery jr., J. E. Peralta, F. Ogliaro, M. Bearpark, J. J. Heyd, E. Brothers, K. N. Kudin, V. N. Staroverov, R. Kobayashi, J. Normand, K. Raghavachari, A. Rendell, J. C. Burant, S. S. Iyengar, J. Tomasi, M. Cossi, N. Rega, N. J. Millam, M. Klene, J. E. Knox, J. B. Cross, V. Bakken, C. Adamo, J. Jaramillo, R. Gomperts, R. E. Stratmann, O. Yazyev, A. J. Austin, R. Cammi, C. Pomelli, J. W. Ochterski, R. L. Martin, K. Morokuma, V. G. Zakrzewski, G. A. Voth, P. Salvador, J. J. Dannenberg, S. Dapprich, A. D. Daniels, Ö. Farkas, J. B. Foresman, J. V. Ortiz, J. Cioslowski and D. J. Fox, *Gaussian 09*, Gaussian Inc., Wallingford CT, USA, 2009.
15 J. Kästner, J. M. Carr, T. W. Keal, W. Thiel, A. Wander and P. Sherwood, *J. Phys. Chem. A*, 2009, **113**, 11856.
16 *ChemShell*, a Computational Chemistry Shell. www.chemshell.org
17 P. Sherwood, A. H. de Vries, M. F. Guest, G. Schreckenbach, C. R. A. Catlow, S. A. French, A. A. Sokol, S. T. Bromely, W. Thiel, A. J. Turner, S. Billeter, F. Terstegen, S. Thiel, J. Kendrick, S. C. Rogers, J. Casci, M. Watson, F. King, E. Karlsen, M. Sjøvoll, A. Fahmi, A. Schäfer and C. Lennartz, *J. Mol. Struct. (THEOCHEM)*, 2003, **632**, 1.
18 R. Ahlrichs and K. May, *Phys. Chem. Chem. Phys.*, 2000, **2**, 943.
19 F. Weigend and R. Ahlrichs, *Phys. Chem. Chem. Phys.*, 2005, **7**, 3297.
20 A. D. Becke, *J. Chem. Phys.*, 1993, **98**, 5648.
21 C. T. Lee, W. T. Yang and R. G. Parr, *Phys. Rev. B*, 1988, **37**, 785.
22 F. Neese, *Orca*, an Ab Initio, Density Functional and Semiempirical Electronic Structure Program Package, version 2.8; Universität Bonn: Bonn, Germany, 2010.
23 A. Klamt and G. Schuuramann, *J. Chem. Soc., Perkin Trans.*, 1993, **2**, 793.
24 F. Neese, F. Wennmohs, A. Hansen and U. Becker, *Chem. Phys.*, 2009, **356**, 98.
25 K. Eichkorn, O. Treutler, H. Ohm, M. Haser and R. Ahlrichs, *Chem. Phys. Lett.*, 1995, **242**, 652.
26 K. Eichkorn, F. Weigend, O. Treutler and R. Ahlrichs, *Theor. Chem. Acc.*, 1997, **97**, 119.
27 C. J. van Wüllen, *J. Chem. Phys.*, 1998, **109**, 392.
28 L. Noodleman, *J. Chem. Phys.*, 1981, **74**, 5737.
29 L. Noodleman, D. A. Case and A. Aizman, *J. Am. Chem. Soc.*, 1988, **110**, 1001.
30 L. Noodleman and E. R. Davidson, *Chem. Phys.*, 1986, **109**, 131.
31 L. Noodleman, J. G. Norman, J. H. Osborne, A. Aizman and D. A. Case, *J. Am. Chem. Soc.*, 1985, **107**, 3418.
32 L. Noodleman, C. Y. Peng, D. A. Case and J. M. Monesca, *Coord. Chem. Rev.*, 1995, **144**, 199.
33 T. Soda, Y. Kitagawa, T. Onishi, Y. Takano, Y. Shigetu, H. Nagao, Y. Yoshioka and K. Yamaguchi, *Chem. Phys. Lett.*, 2000, **319**, 223.
34 K. Yamaguchi, Y. Takahara and T. Fueno in *Applied Quantum Chemistry*, ed. V. H. Smith, Reidel, Dordrecht, The Netherlands, 1986, pp. 155.
35 *Molekel*, Advanced Interactive 3D-Graphics for Molecular Sciences, Swiss National Supercomputing Center. http://www.cscs.ch/molekel.

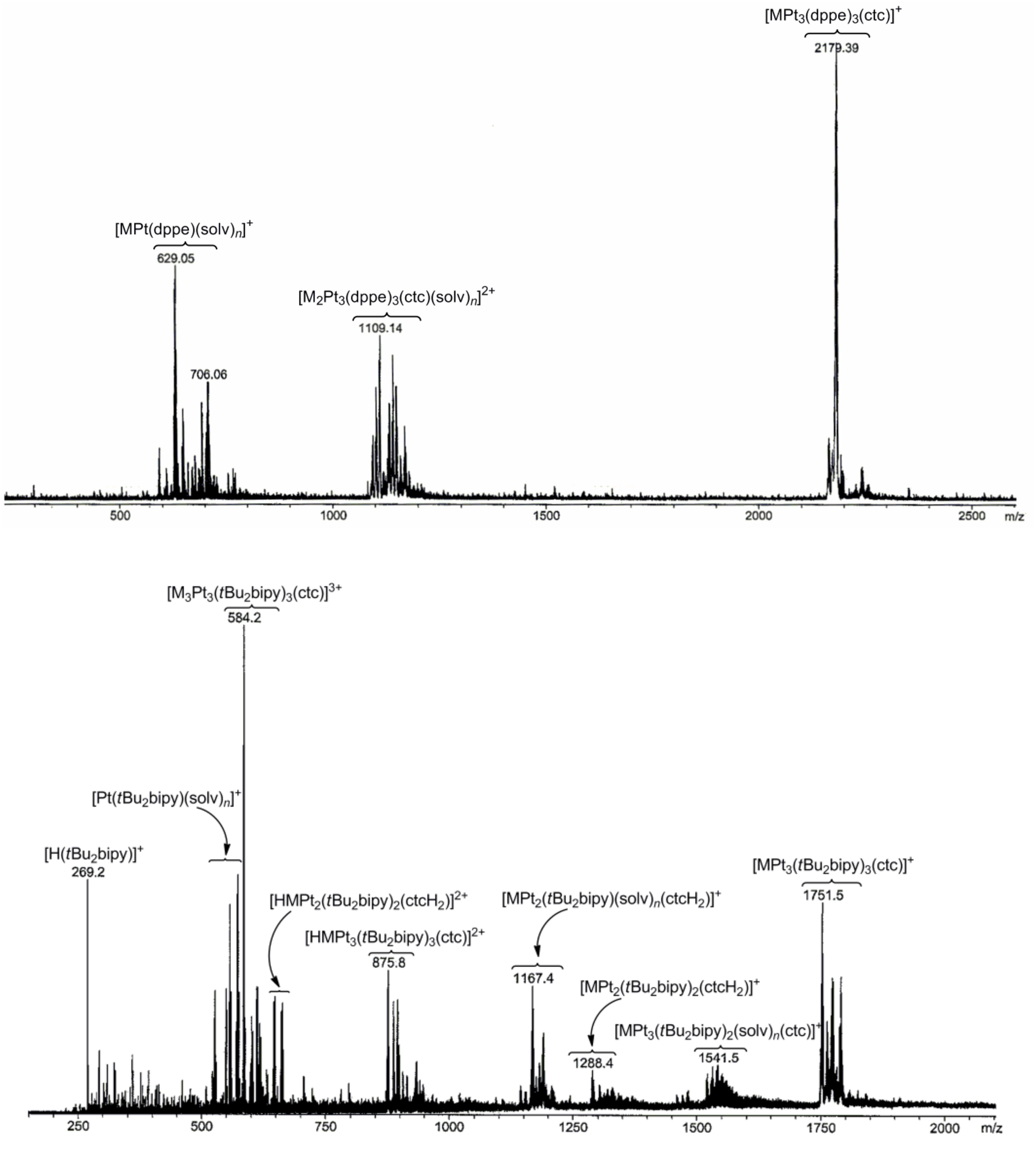

Figure S1. Electrospray mass spectra of the new complexes **2** (top, from dmso solution) and **3** (bottom, from MeCN solution), showing the assignments of groups of peaks (M^+ = H^+, Na^+ and/or K^+; solv = MeCN, dmso, H_2O and/or HCO_2^-).

The molecular ions of the intact compounds have m/z = 2140.8 ([**2**]$^+$) and 1750.8 ([**3**]$^+$). Individual peak assignments are listed in the Experimental Section.

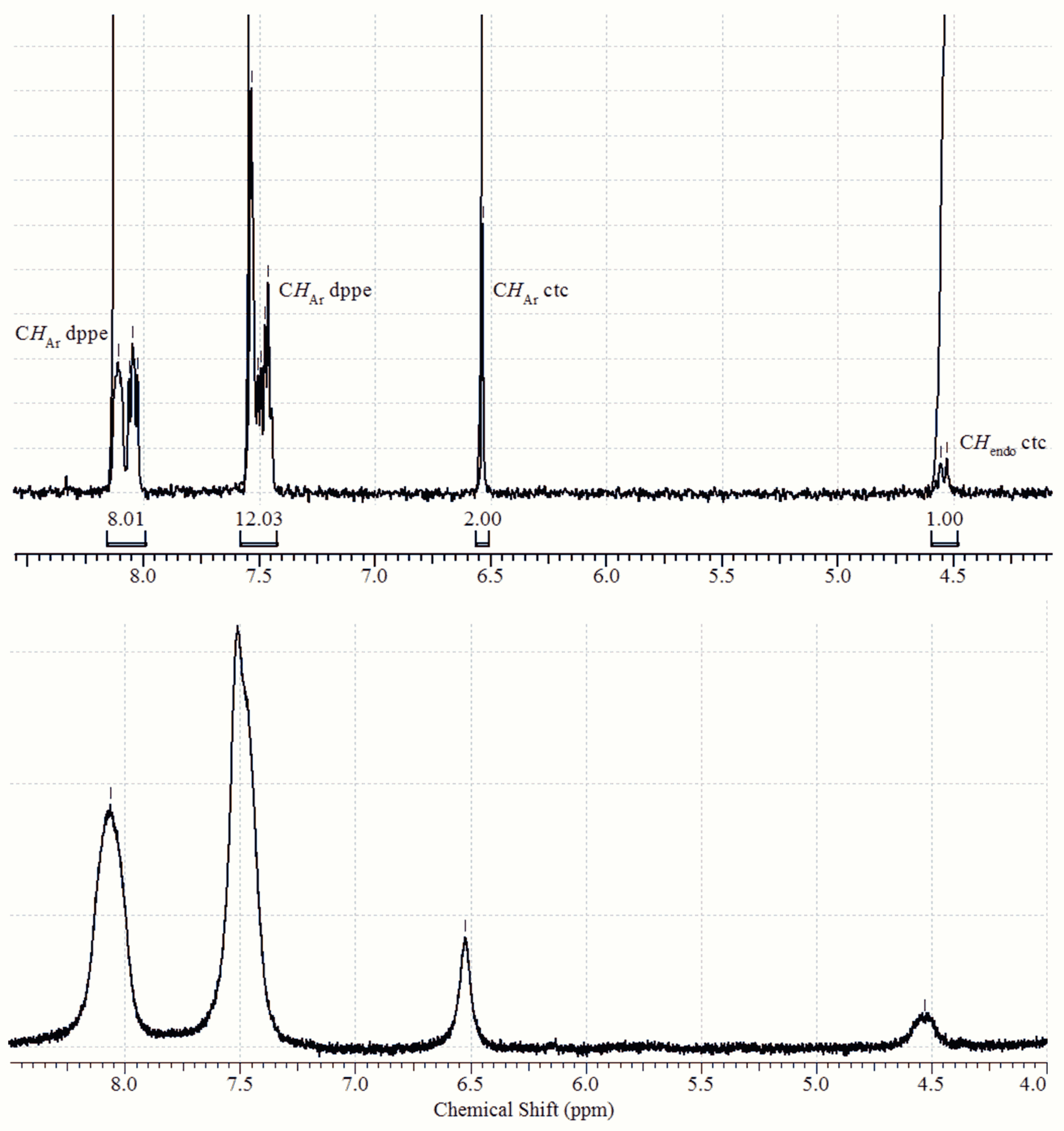

Figure S2. Partial ^{1}H NMR spectra of **2** in $\{CD_3\}_2SO$ at 298 K, under an N_2 atmosphere (top) and in air (bottom). The CH_{exo} ctc peak (Fig. S3) is obscured under the water resonance (not shown).

The peak broadening of the aerobic sample reflects partial air oxidation of the $[ctc]^{6-}$ ligand (Fig. S4).

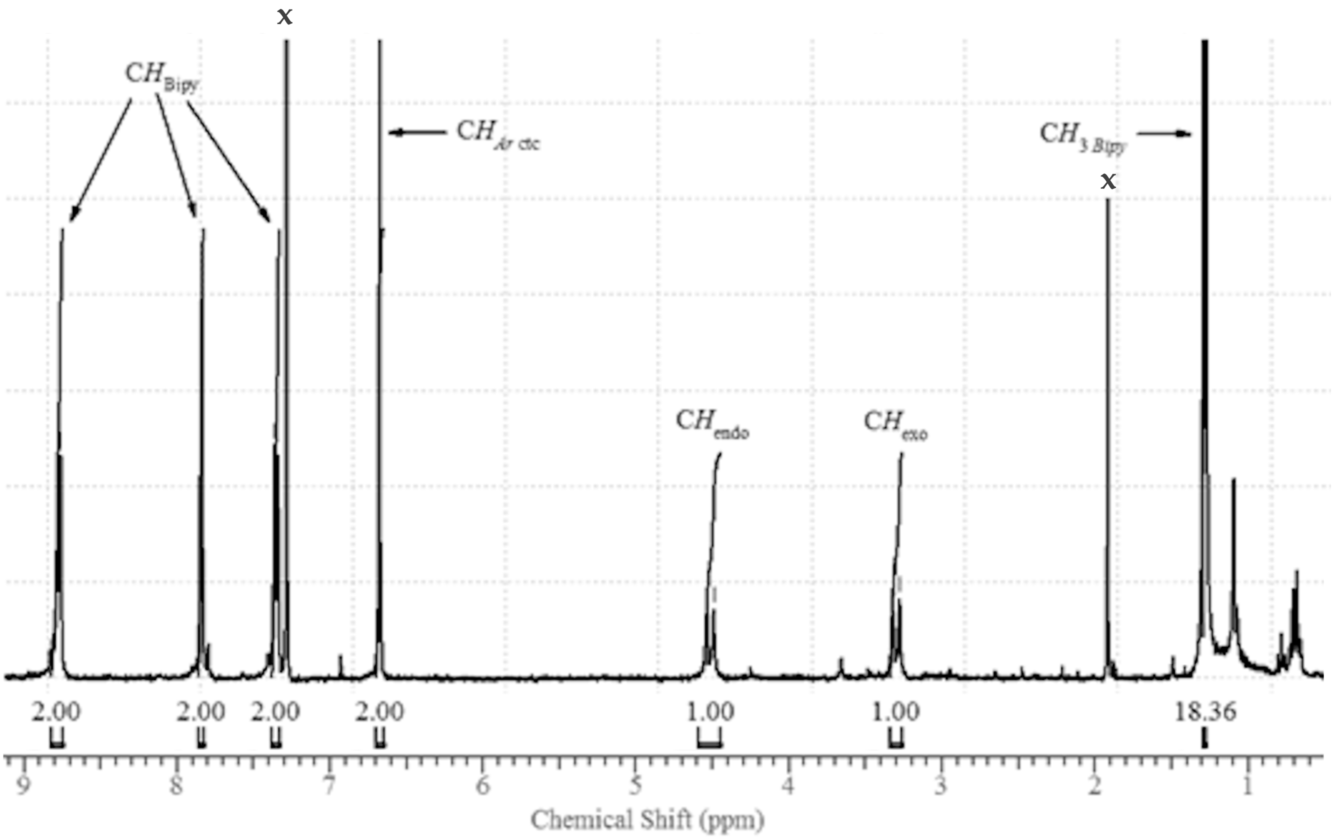

Figure S3. ^{1}H NMR spectrum of **3** in $CDCl_3$ at 298 K, under an N_2 atmosphere. The splitting of the $[ctc]^{6-}$ CH_2 groups into *endo* and *exo* environments reflects its rigid bowl-shaped conformation.

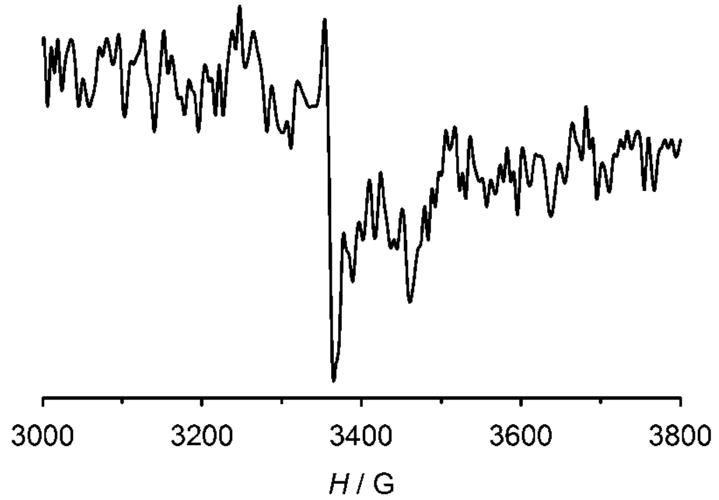

Figure S4. *X*-band EPR spectrum of freshly prepared **3** in aerobic $CHCl_3$ solution at 150 K.

The weak signal at g = 2.03 indicates that the NMR line-broadening observed in air-exposed solutions of the compound (Fig. S4) reflects partial oxidation of the $[ctc]^{6-}$ ligand.

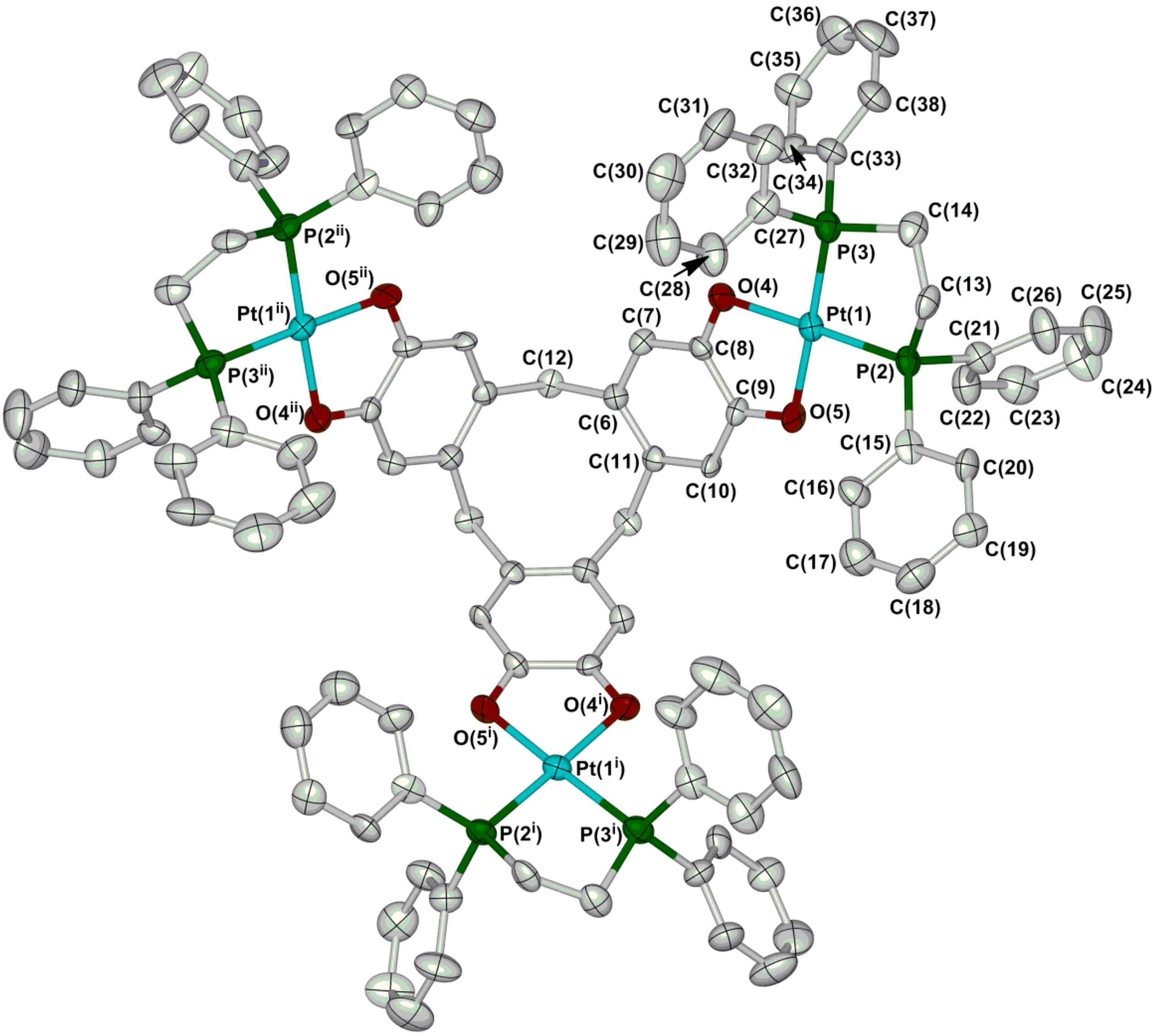

Figure S5. View of the [{Pt(dppe)}$_3$(ctc)] half-molecule in **2**·H_2O·8dma, showing the full atom numbering scheme employed. Displacement ellipsoids are at the 50 % probability level, and all H atoms have been omitted for clarity. Symmetry codes: (i) 1–*y*, 1+*x*–*y*, z; (ii) –*x*+*y*, 1–*x*, z.

This figure is identical to Fig. 1 in the main paper, but with all non-H atoms labelled. Colour code: C, white; O, red; P, green; Pt, cyan.

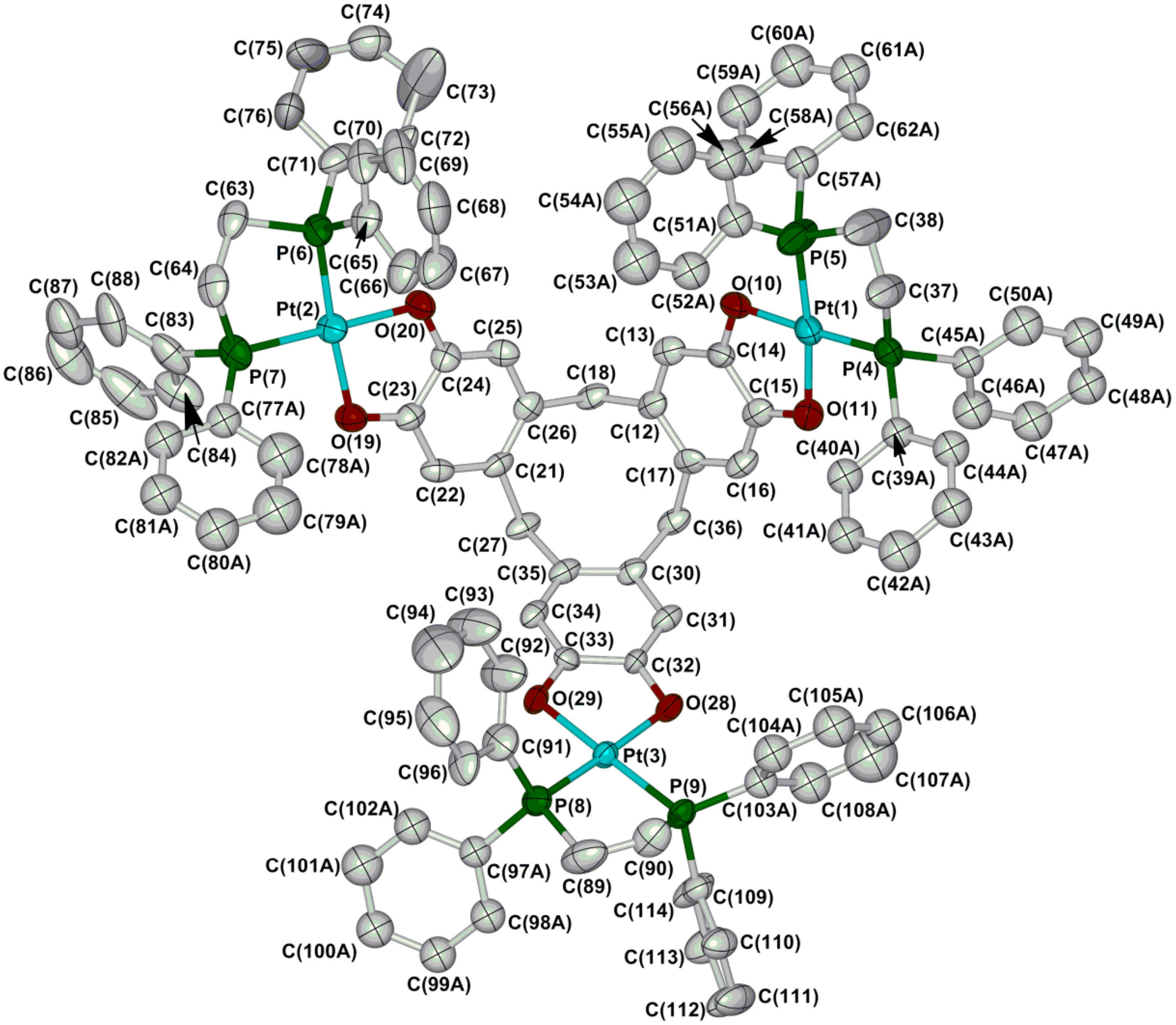

Figure S6. View of the [{Pt(dppe)}$_3$(ctc)] molecule in **2**·H_2O·$2H_2O$·1.3dma·0.5MeOH, showing the atom numbering scheme employed. Only one orientation of the disordered phenyl groups has been included. Displacement ellipsoids are at the 50 % probability level, and H atoms have been omitted for clarity.

Colour code: C, white; O, red; P, green; Pt, cyan.

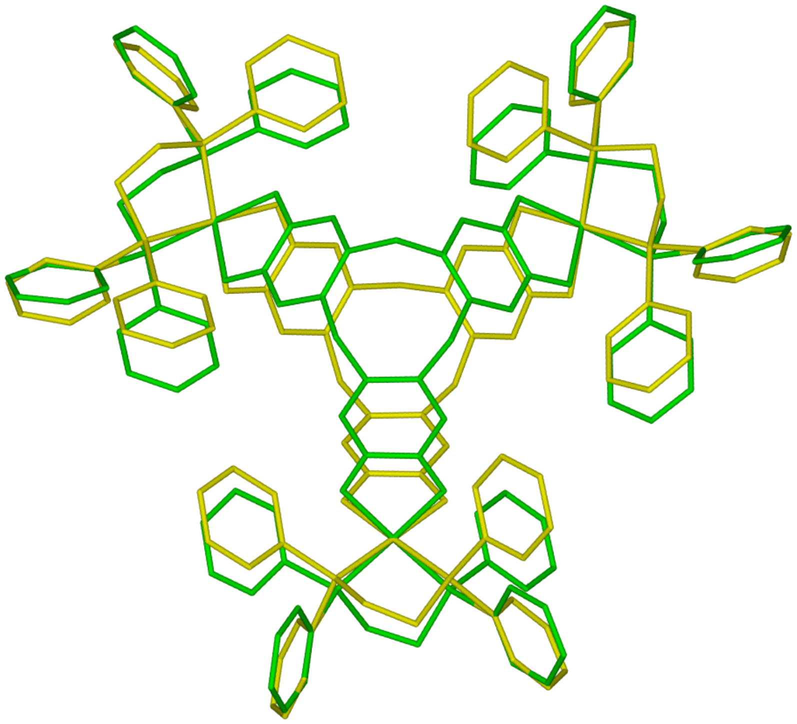

Figure S7. View of the disorder of the complex in **2**·H_2O·8dma across a crystallographic mirror plane. The two disorder orientations are coloured green and yellow.

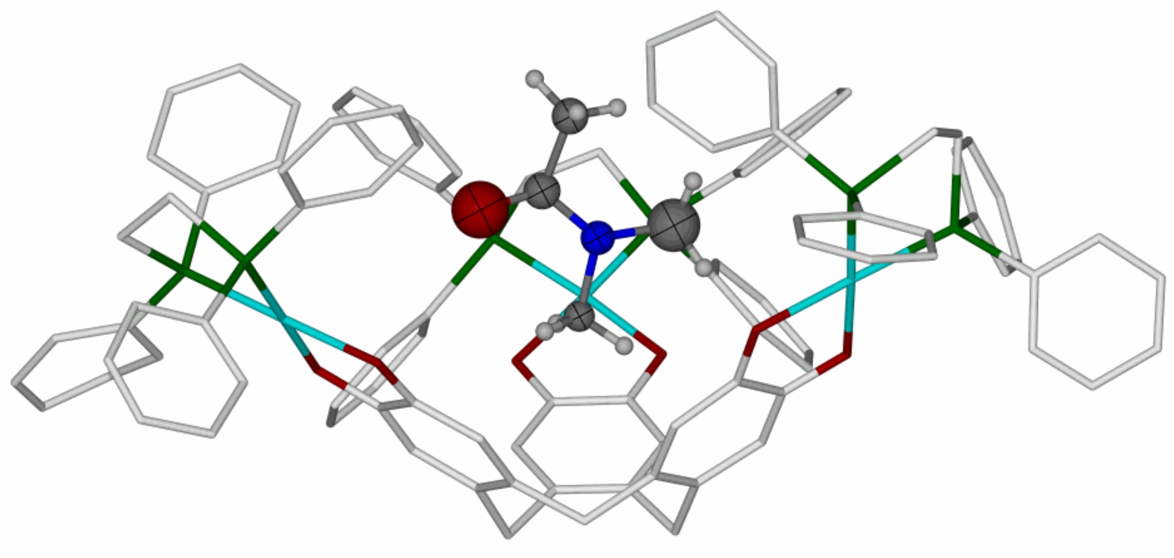

Figure S8. Alternative view of **2**·2H_2O·1.3dma·0.5MeOH, showing inclusion of dma into the cavity of the complex. Only one disorder orientation is given of the included dma molecule, and of the disordered phenyl groups in the complex. H atoms in the complex have also been omitted for clarity. Displacement ellipsoids in the dma molecule are at the 50 % probability level, while all other atoms have arbitrary radii.

Colour code: C{complex}, white; C{dma}, dark grey; H, pale grey; N, blue; O, red; P, green; Pt, cyan.

This orientation of the dma molecule, and the other disorder orientation (not shown), both have a methyl group pointing into the hydrophobic cavity of the complex. That closely resembles that seen for other solvents included into the cavity of H_6ctc and its complexes, including **1**.[1,2]

Table S2. Selected bond distances and angles for the two solvates of [{Pt(dppe)}$_3$(ctc)] (**2**) (Å, º). See Figs. S5 and S6 for the atom numbering scheme employed.

**2**·H_2O·8MeC(O)NMe_2		**2**·2H_2O·1.3MeC(O)NMe_2·0.5MeOH	
Pt(1)−P(2)	2.218(5)	Pt(1)−P(4)	2.223(3)
Pt(1)−P(3)	2.234(5)	Pt(1)−P(5)	2.208(3)
Pt(1)−O(4)	2.043(13)	Pt(1)−O(10)	2.025(7)
Pt(1)−O(5)	2.050(13)	Pt(1)−O(11)	2.060(7)
		Pt(2)−P(6)	2.215(3)
		Pt(2)−P(7)	2.220(3)
		Pt(2)−O(19)	2.044(6)
		Pt(2)−O(20)	2.025(7)
		Pt(3)−P(8)	2.218(3)
		Pt(3)−P(9)	2.208(2)
		Pt(3)−O(28)	2.039(6)
		Pt(3)−O(29)	2.052(5)
P(2)−Pt(1)−P(3)	86.08(19)	P(4)−Pt(1)−P(3)	85.61(11)
P(2)−Pt(1)−O(4)	178.7(4)	P(4)−Pt(1)−O(10)	177.02(18)
P(2)−Pt(1)−O(5)	95.6(4)	P(4)−Pt(1)−O(11)	100.7(2)
P(3)−Pt(1)−O(4)	95.2(4)	P(5)−Pt(1)−O(10)	91.44(19)
P(3)−Pt(1)−O(5)	178.3(4)	P(5)−Pt(1)−O(11)	173.3(2)
O(4)−Pt(1)−O(5)	83.1(5)	O(10)−Pt(1)−O(11)	82.3(3)
		P(6)−Pt(2)−P(7)	85.53(10)
		P(6)−Pt(2)−O(19)	176.4(2)
		P(6)−Pt(2)−O(20)	93.70(19)
		P(7)−Pt(2)−O(19)	97.7(2)
		P(7)−Pt(2)−O(20)	179.1(2)
		O(19)−Pt(2)−O(20)	83.1(3)
		P(8)−Pt(3)−P(9)	85.41(10)
		P(8)−Pt(3)−O(28)	176.43(19)
		P(8)−Pt(3)−O(28)	95.98(19)
		P(9)−Pt(3)−O(29)	94.59(17)
		P(9)−Pt(3)−O(29)	178.56(19)
		O(29)−Pt(3)−O(29)	84.1(2)
Δ{Pt(1)}	–1.7(3)	*Δ*{Pt(1)}	–2.1(2)
		Δ{Pt(2)}	–2.5(4)
		Δ{Pt(3)}	–2.0(3)

Δ is a bond-valence sum parameter giving the oxidation state of dioxolene groups, which takes the values of 0, –1 and –2 for the q, sq and cat levels respectively.[36] Although there is some scatter, all the values in the Table are consistent with a platinum(II)/catecholate centre, as expected.

36 O. Carugo, C. B. Castellani, K. Djinović and M. Rizzi, *J. Chem. Soc., Dalton Trans.*, 1992, 837; S. Brown, *Inorg. Chem.*, 2012, **51**, 1251.

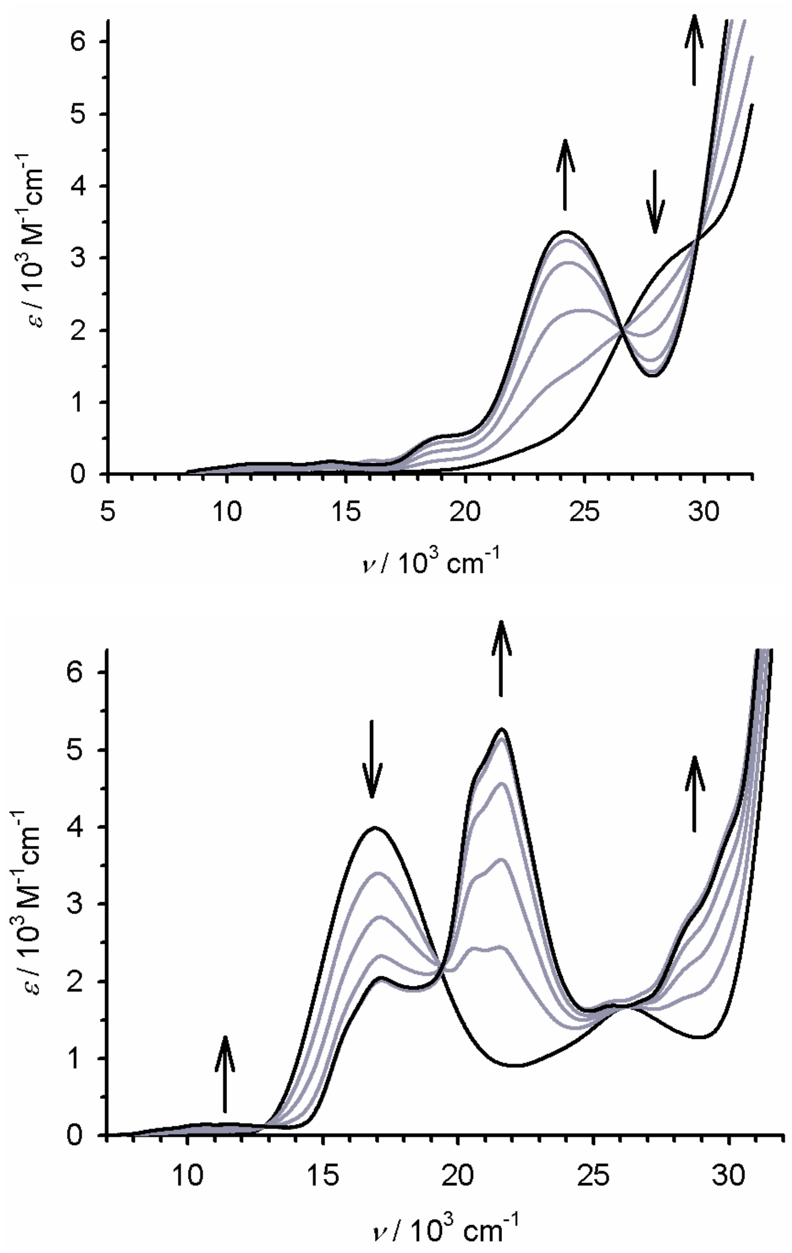

Figure S9. Titration of the cat→sq oxidation of mononuclear [Pt(dppe)(DBCat)] (**4**, top) and [Pt(*t*Bu_2bipy)(DBCat)] (**5**, bottom) with [Fc]PF_6 in CH_2Cl_2 at 298 K, monitored by UV/vis/NIR spectroscopy. Data from these graphs are listed in Table 2 in the main article.

The spectra for the $\mathbf{5}/[\mathbf{5}^{\bullet}]^{+}$ oxidation resemble those previously reported for this compound, which were measured under the same conditions.[37] Data for the $\mathbf{4}/[\mathbf{4}^{\bullet}]^{+}$ process have not been reported before.

37 J. Best, I. V. Sazanovich, H. Adams, R. D. Bennett, E. S. Davies, A. J. H. M. Meijer, M. Towrie, S. A. Tikhomirov, O. V. Bouganov, M. D. Ward and J. A. Weinstein, *Inorg. Chem.*, 2010, **49**, 10041.

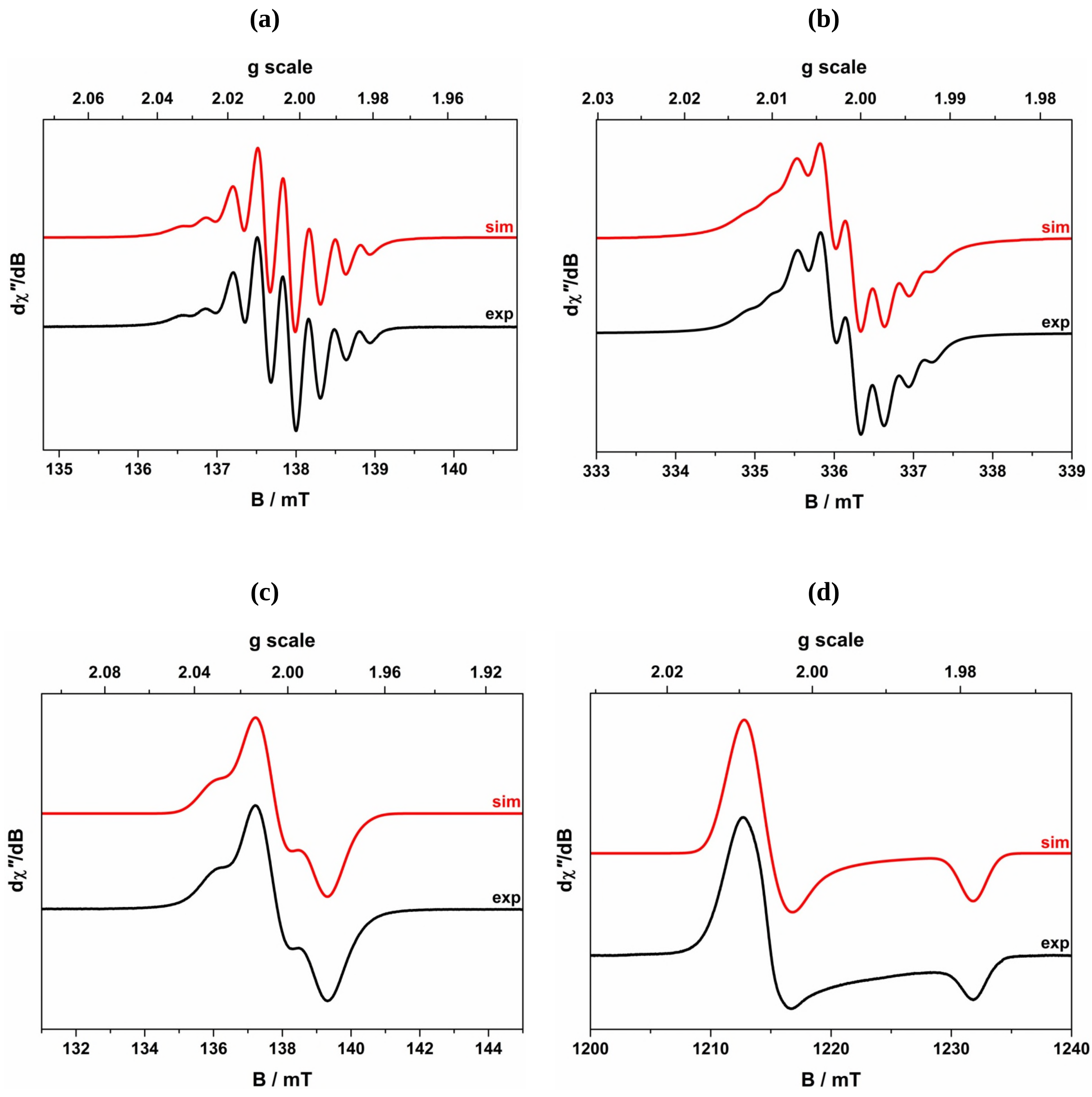

Figure S10. Experimental and simulated EPR spectra of $[\mathbf{4}^{\bullet}]^{+}$ in CH_2Cl_2/THF solution:
(a) S-band spectrum (ν = 3.8599 GHz; power, 3.0 mW; modulation, 0.05 mT) at 210 K;
(b) X-band spectrum (ν = 9.4179 GHz; power, 2.0 mW; modulation, 0.02 mT) at 230 K;
(c) S-band spectrum (ν = 3.8671 GHz; power, 4.8 mW; modulation, 0.01 mT) at 140 K;
(d) Q-band spectrum (ν = 33.985 GHz; power, 0.013 mW; modulation, 0.2 mT) at 80 K.

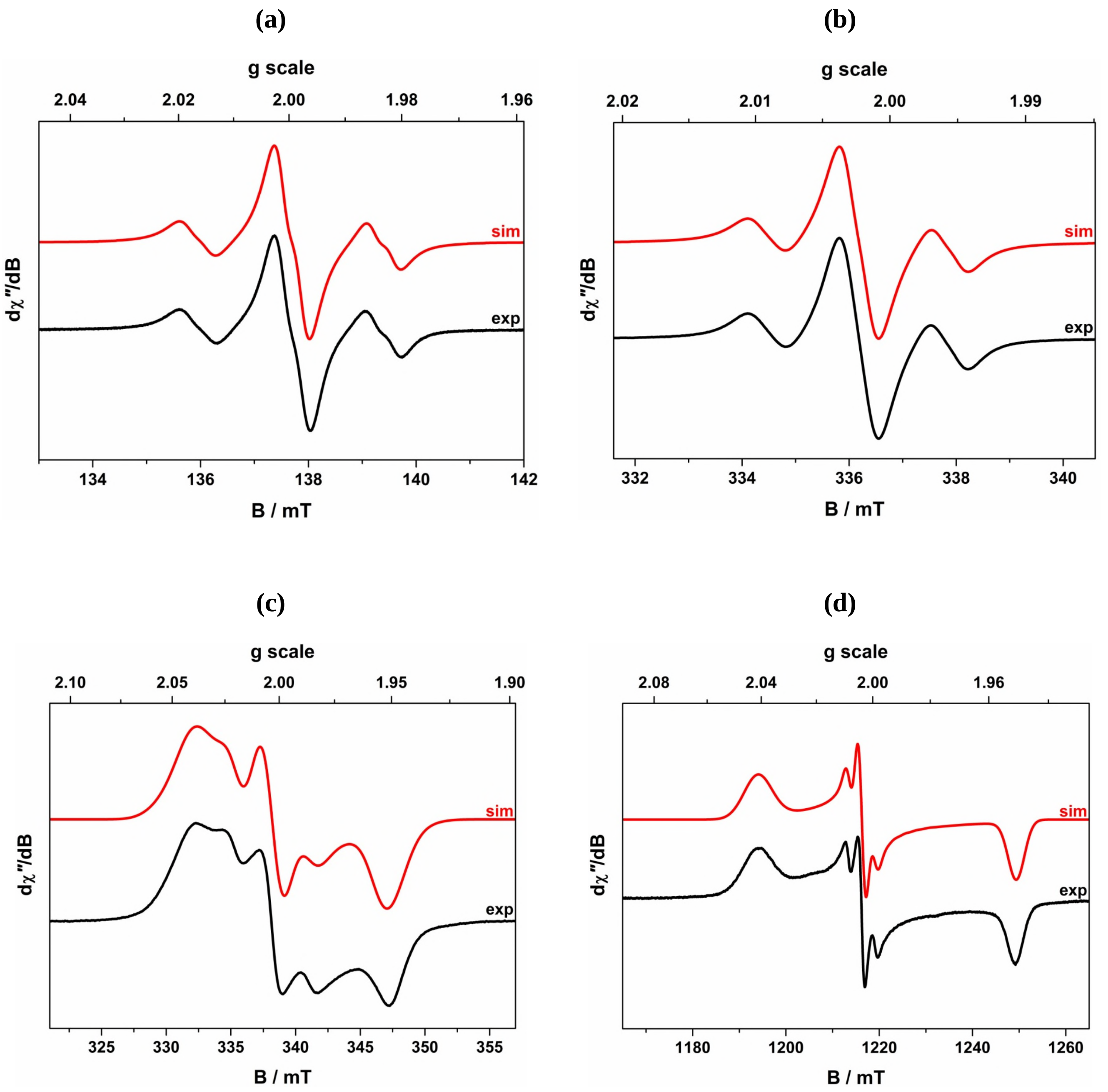

Figure S11. Experimental and simulated EPR spectra of $[\mathbf{5}^{\bullet}]^{+}$ in CH_2Cl_2/THF solution:

(a) S-band spectrum (ν = 3.8593 GHz; power, 3.0 mW; modulation, 0.06 mT) at 230 K;
(b) X-band spectrum (ν = 9.4223 GHz; power, 2.0 mW; modulation, 0.01 mT) at 230 K;
(c) X-band spectrum (ν = 9.4559 GHz; power, 0.63 mW; modulation, 0.3 mT) at 30 K;
(d) Q-band spectrum (ν = 33.990 GHz; power, 0.13 mW; modulation, 0.3 mT) at 80 K.

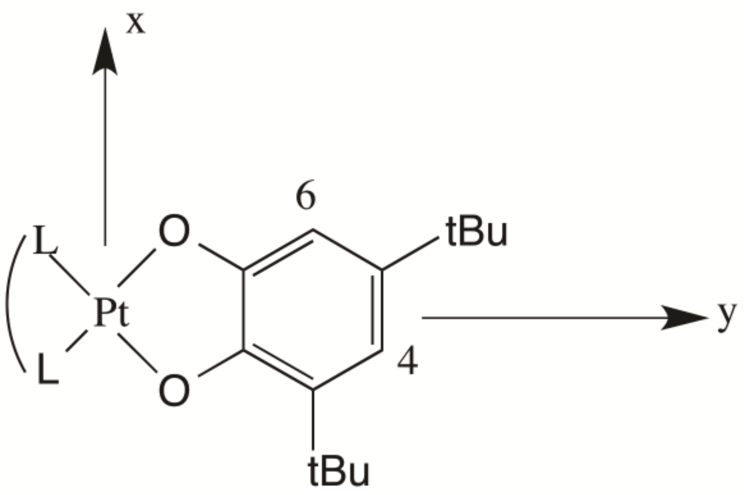

Figure S12. Axis and numbering systems for **[4$^{\bullet}$]$^{+}$** and **[5$^{\bullet}$]$^{+}$**.

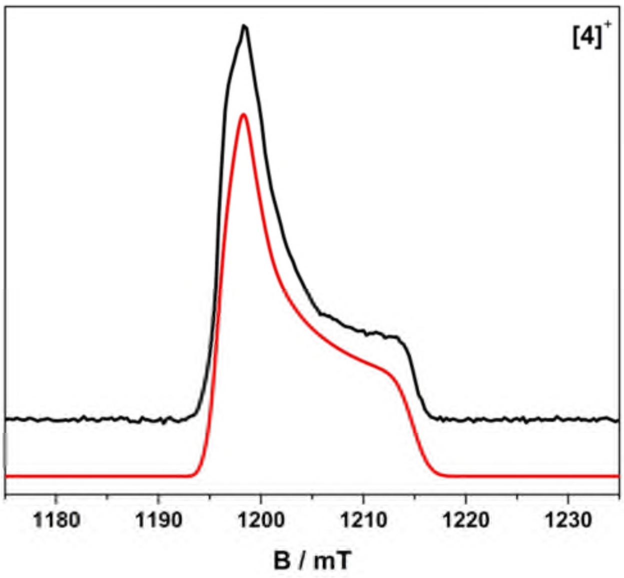

Figure S13. Q-band ESE-detected EPR spectrum of **[4$^{\bullet}$]$^{+}$** recorded in CH_2Cl_2/THF solution at 20 K (ν = 33.638 GHz; $t_{\pi/2}$ = 22 ns; τ = 400 ns). Experimental data are shown by the black line and simulation as the red trace.

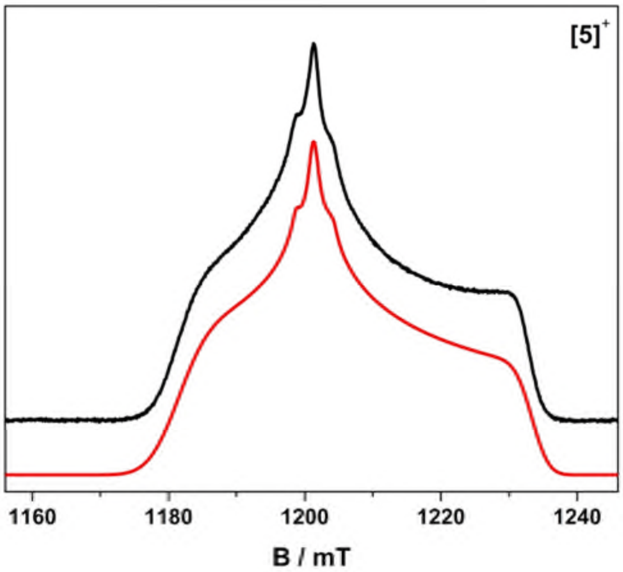

Figure S14. Q-band ESE-detected EPR spectrum of **[5$^{\bullet}$]$^{+}$** recorded in CH_2Cl_2/THF solution at 20 K (ν = 33.710 GHz; $t_{\pi/2}$ = 22 ns; τ = 400 ns). Experimental data are shown by the black line and simulation as the red trace.

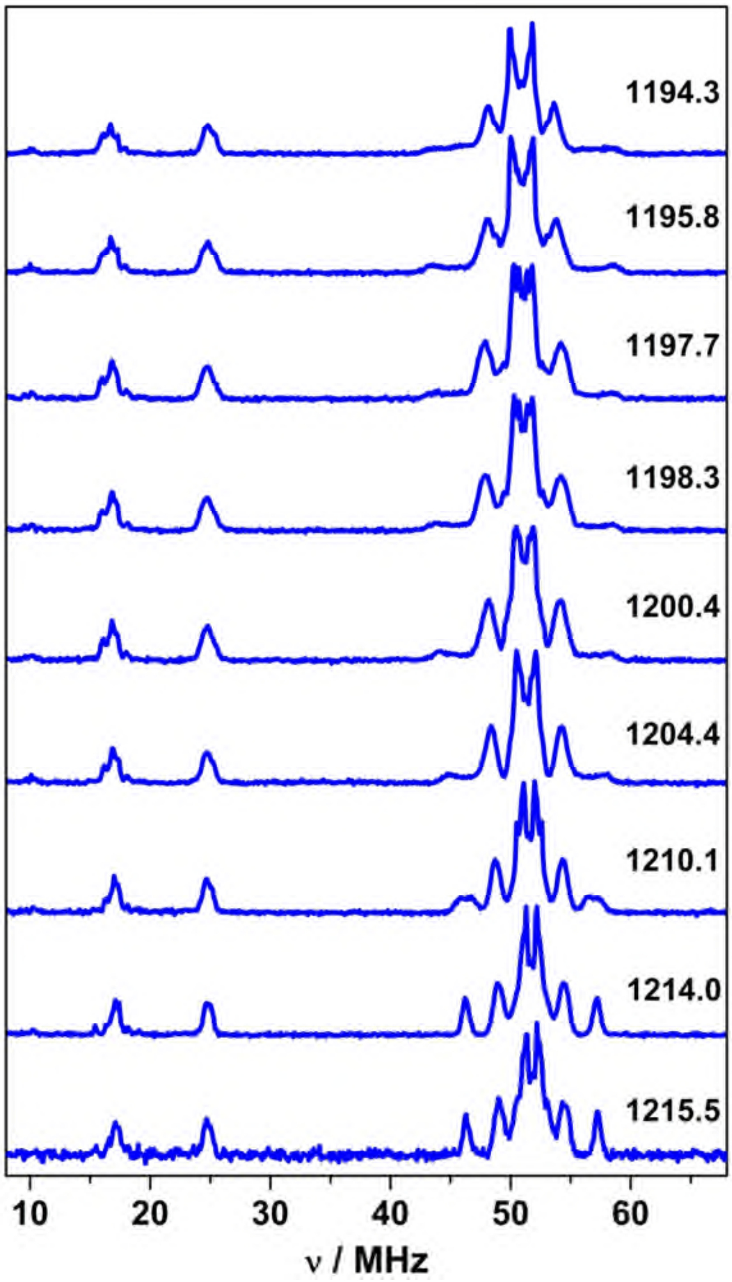

Figure S15. Orientation selected Davies ENDOR spectra of **[4•]**$^+$ recorded in CH_2Cl_2/THF solution at 20 K. Static magnetic fields as shown, corresponding to the echo-detected field-swept spectrum in Figure S15. Experimental conditions: ν_{mw} = 33.638 GHz, t_{RF} = 16 μs, t_{inv} = 200 ns, $t_{\pi/2}$ = 22 ns, τ = 550 ns.

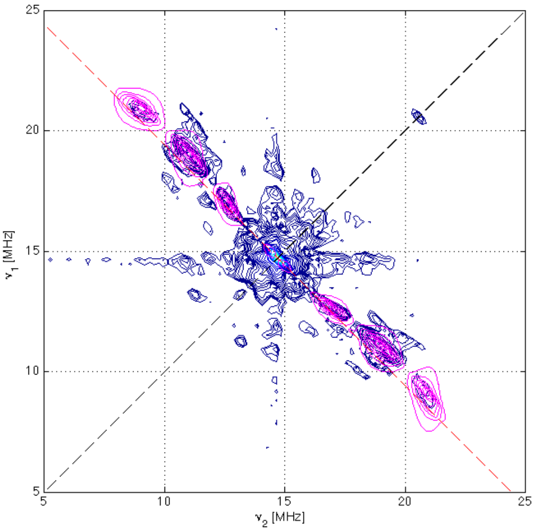

Figure S16. X-band ^{1}H HYSCORE spectrum of **[5$^{\bullet}$]$^+$** with corresponding numerical simulation (pink) using the parameters in Table 3. Experimental conditions: T = 20 K, ν_{mw} = 9.682 GHz, $\nu_n(^1H)$ = 15.09 MHz, $t_{\pi/2}$ = 16 ns, τ = 200 ns, B_0 = 345.3 mT (near g_x, i.e. the maximum in the ESE spectrum).

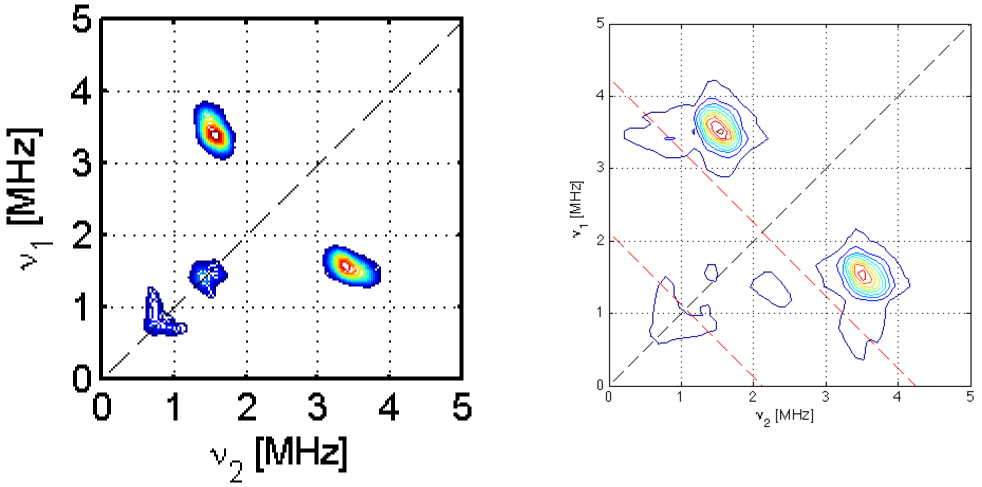

Figure S17. Left: X-band ^{14}N HYSCORE spectra of **[5$^{\bullet}$]$^+$** measured at 345.3 mT (near g_x, i.e. at maximum of ESE spectrum). Experimental conditions: T = 20 K, ν_{mw} = 9.682 GHz, $\nu_n(^{14}N)$ = 1.07 MHz, $t_{\pi/2}$ = 16 ns, τ = 136 ns. Right: simulation with parameters in Table 3. The principal axis of the quadrupole tensor (modelled as axial; η = 0) is in the xy plane, and their is a small (ca. 0.2 MHz) anisotropic component to the ^{14}N hyperfine tensor.

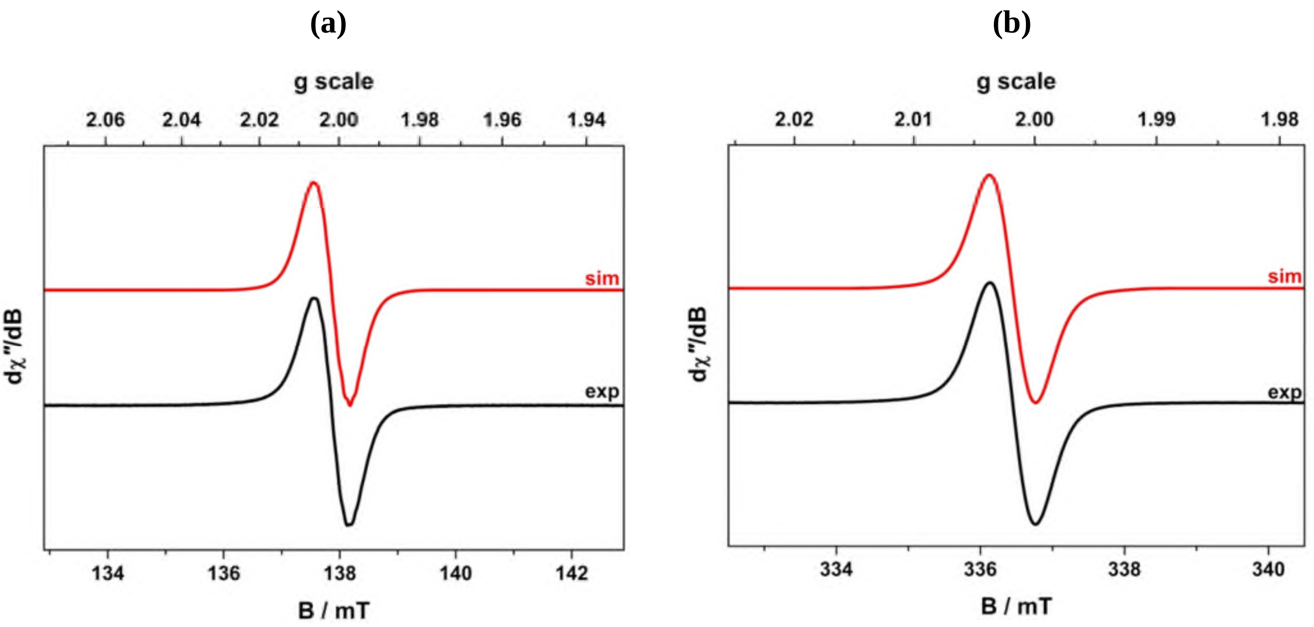

Figure S18. Experimental and simulated EPR spectra of **[1•]**$^{+}$ in CH_2Cl_2/THF solution:
(a) S-band spectrum (ν = 3.8624 GHz; power, 3.0 mW; modulation, 0.07 mT) at 210 K;
(b) X-band spectrum (ν = 9.4265 GHz; power, 2.0 mW; modulation, 0.05 mT) at 230 K.

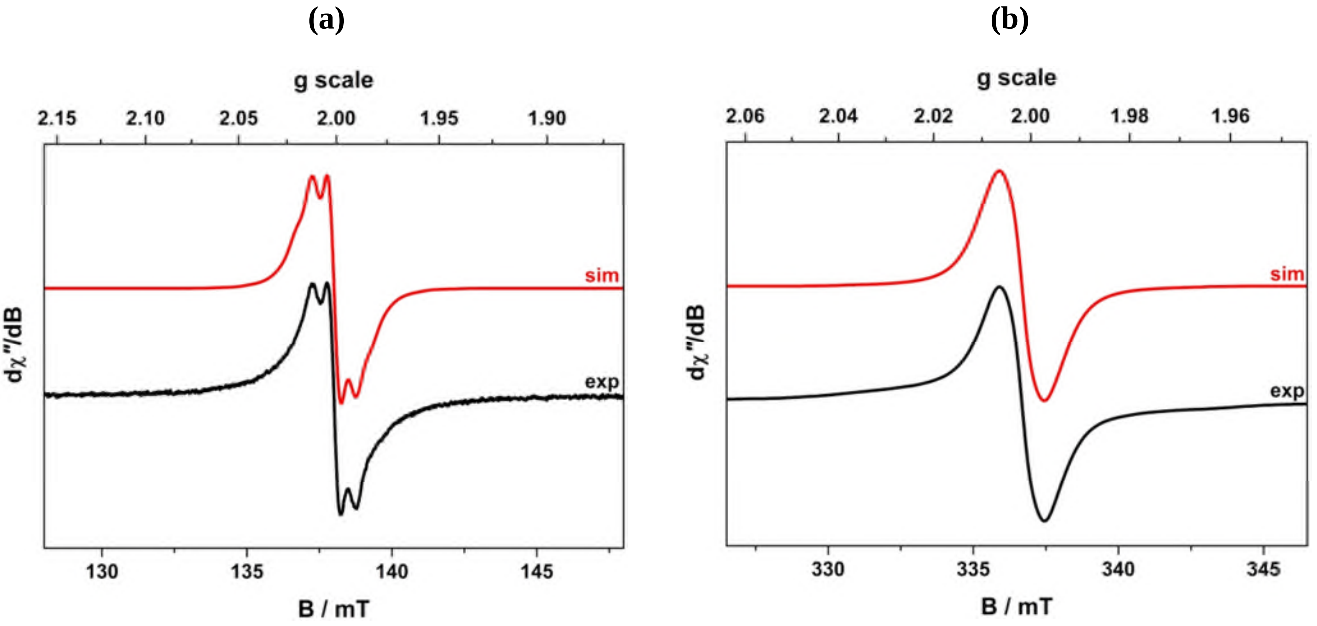

Figure S19. Experimental and simulated EPR spectra of **[3•]**$^{+}$ in CH_2Cl_2/THF solution:
(a) S-band spectrum (ν = 3.86524 GHz; power, 94.9 mW; modulation, 0.1 mT) at 210 K;
(b) X-band spectrum (ν = 9.4324 GHz; power, 20.0 mW; modulation, 0.2 mT) at 230 K.

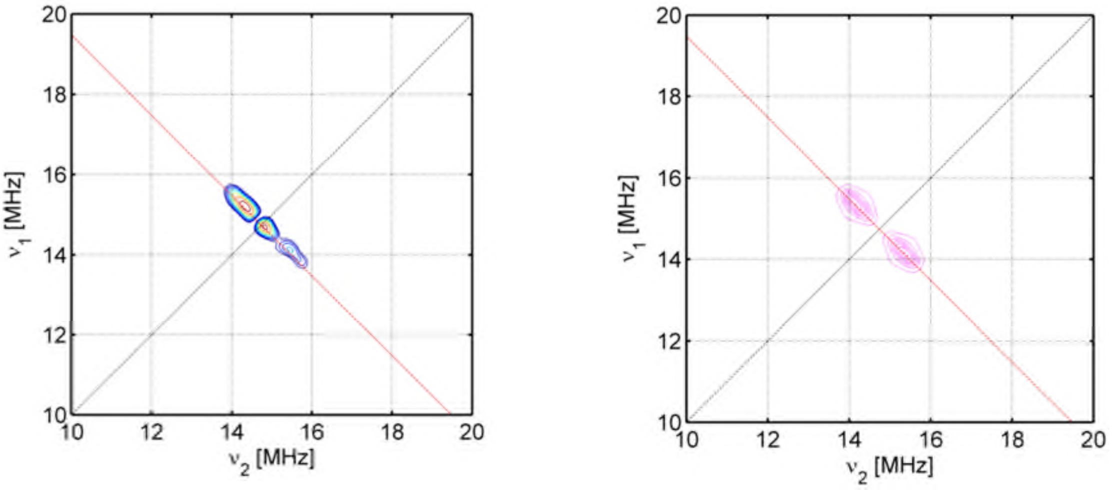

Figure S20. X-band ^{1}H HYSCORE spectrum of $[\mathbf{1}^{\bullet}]^+$ with corresponding numerical simulation (pink). Experimental conditions: T = 20 K, ν_{mw} = 9.683 GHz, $\nu_n(^1\text{H})$ = 14.74 MHz, $t_{\pi/2}$ = 16 ns, τ = 200 ns, B_0 = 346.2 mT.

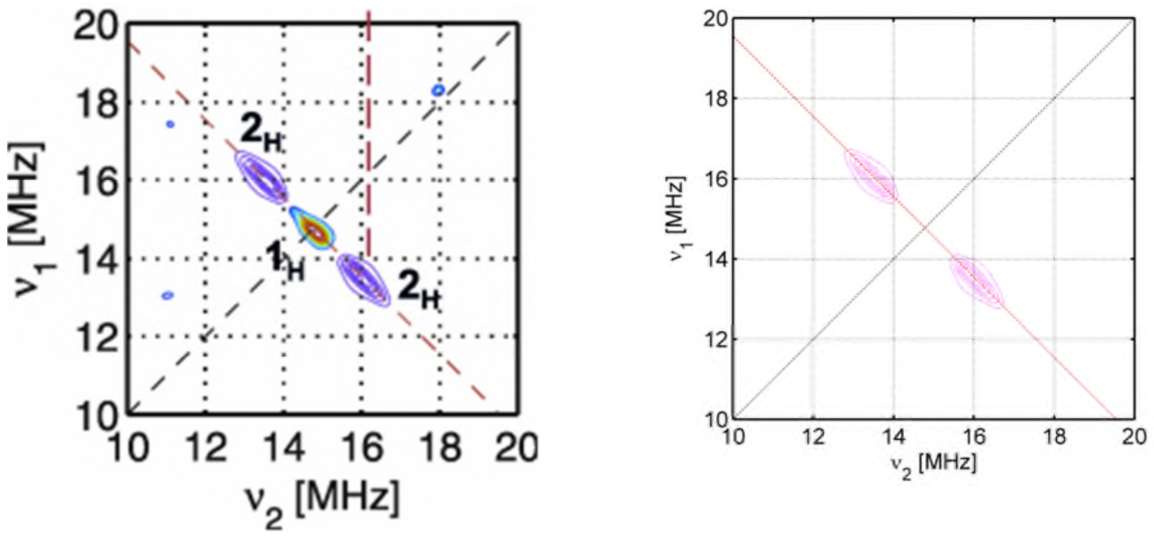

Figure S21. X-band ^{1}H HYSCORE spectrum of $[\mathbf{3}^{\bullet}]^+$ with corresponding numerical simulation (pink). Experimental conditions: T = 20 K, ν_{mw} = 9.702 GHz, $\nu_n(^1\text{H})$ = 14.82 MHz, $t_{\pi/2}$ = 16 ns, τ = 154 ns, B_0 = 348.2 mT.

Table S3. Geometry Optimised Coordinates for $[DBsq^{\bullet}]^{-}$

O	-0.415553	1.180771	0.005773
O	-0.413905	-1.558933	-0.009993
C	-1.517981	0.567932	0.000741
C	-1.512904	-0.930581	-0.004928
C	-2.767929	-1.607988	-0.003811
C	-3.971658	-0.941292	-0.001366
C	-3.954476	0.483606	-0.000403
C	-2.803465	1.243532	0.000289
C	-2.862141	2.781415	0.000551
C	-2.175668	3.339844	1.268628
C	-2.170973	3.339624	-1.265091
C	-4.304281	3.312250	-0.002216
C	-5.289462	-1.735095	0.000293
C	-6.540809	-0.846093	0.000165
C	-5.349367	-2.616367	1.264519
C	-5.351301	-2.619359	-1.261696
H	-2.724819	-2.692392	-0.005625
H	-4.904347	0.991979	-0.000061
H	-1.133124	3.030976	1.319303
H	-2.685585	2.981123	2.166711
H	-2.222455	4.432868	1.272102
H	-2.217613	4.432650	-1.268945
H	-2.677564	2.980746	-2.165004
H	-1.128252	3.030704	-1.311885
H	-4.283548	4.405046	-0.002299
H	-4.859739	2.991399	0.881800
H	-4.856349	2.991055	-0.888231
H	-6.591165	-0.209227	-0.885955
H	-6.589949	-0.207094	0.884805
H	-7.428843	-1.483110	0.001581
H	-6.284618	-3.182801	1.291373
H	-5.302517	-1.994944	2.162846
H	-4.523763	-3.328346	1.300812
H	-4.525827	-3.331475	-1.297628
H	-5.305902	-2.000058	-2.161554
H	-6.286594	-3.185832	-1.285722

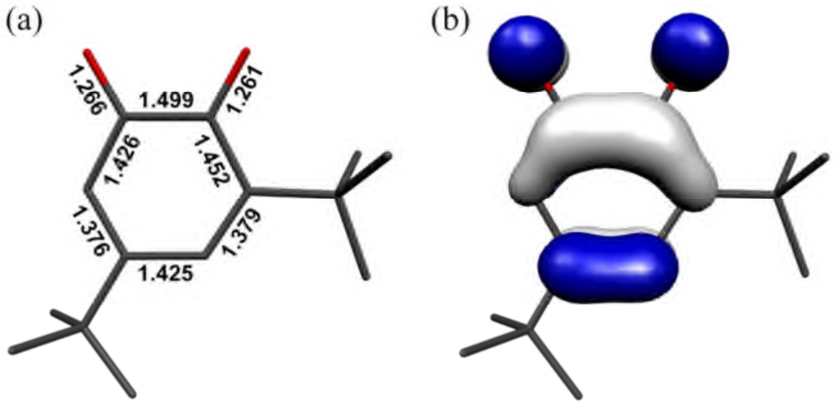

Figure S22. (a) Geometry optimised structure with key bond distances, and (b) isosurface plot of the ground state molecular orbital for $[DBsq^{\bullet}]^{-}$.

Table S4. Comparison of experimental metrics in [Pt(L)(DBcat)] (L = dppe, bipy) with calculated metrics for the corresponding $S = {}^{1}/_{2}$ species $[Pt(L)(DBsq^{\bullet})]^{+}$ (Å, °)

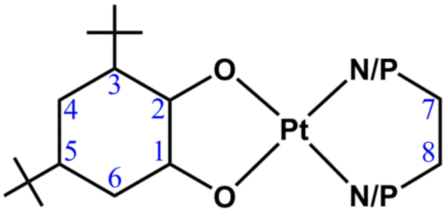

	[Pt(dppe)(DBcat)][a] exptl	$[Pt(dppe)(DBsq^{\bullet})]^{+}$ calcd	[Pt(bipy)(DBcat)][a] exptl	$[Pt(bipy)(DBsq^{\bullet})]^{+}$ calcd
Pt–N/P(1)	2.222(1)	2.245	1.978(1)	2.012
Pt–N/P(2)	2.206(1)	2.242	1.992(1)	2.007
Pt–O(1)	2.038(3)	2.047	1.984(1)	2.008
Pt–O(2)	2.040(3)	2.057	1.982(1)	2.017
O(1)–C(1)	1.368(5)	1.306	1.360(2)	1.309
O(2)–C(2)	1.369(5)	1.303	1.358(2)	1.305
C(1)–C(2)	1.409(6)	1.452	1.414(2)	1.450
C(1)–C(6)	1.395(6)	1.402	1.387(2)	1.396
C(2)–C(3)	1.422(6)	1.431	1.403(2)	1.428
C(3)–C(4)	1.402(6)	1.373	1.409(2)	1.377
C(4)–C(5)	1.388(6)	1.439	1.401(2)	1.433
C(5)–C(6)	1.403(6)	1.375	1.403(2)	1.380
C_{arom}–C_{arom}[b]	1.403(6)	1.412	1.403(2)	1.411
N/P(1)–C(7)	1.823(4)	1.847	1.362(2)	1.359
N/P(2)–C(8)	1.849(4)	1.847	1.364(2)	1.358
C(7)–C(8)	1.555(6)	1.535	1.468(2)	1.467
τ[c]	6.1	0.5	5.5	0.0

[a] Data are taken from ref 36. [b] Average C–C distance in the $[DBsq^{\bullet}]^{-}$ ligand. [c] Dihedral angle between the $\{PtO_2\}$ and $\{Pt(N/P)_2\}$ mean planes.

Table S5. Geometry Optimised Coordinates for **[4•]**$^{+}$

Pt	8.847128	9.017855	23.415120
P	10.120621	7.551590	22.294215
P	10.496338	9.007839	24.938293
O	7.610662	10.363814	24.359681
O	7.297607	9.071141	22.077825
C	6.508234	10.591704	23.703008
C	5.492919	11.488283	24.163566
C	4.397915	11.614034	23.344690
C	4.211944	10.924191	22.095464
C	5.193085	10.062453	21.665441
C	6.341876	9.880854	22.447834
C	11.513867	6.975171	23.360396
C	11.973706	8.125607	24.266718
C	2.930994	11.180714	21.303204
C	1.712776	10.782191	22.167184
C	2.844161	12.683813	20.953380
C	2.883382	10.375985	19.997551
C	5.637021	12.249466	25.488797
C	4.419487	13.144524	25.766442
C	5.758979	11.246211	26.657087
C	6.886093	13.156833	25.438092
H	3.608645	12.280561	23.653337
H	5.121475	9.510672	20.740749
H	11.128663	6.143844	23.954855
H	12.333294	6.591835	22.752266
H	12.545709	8.861889	23.697884
H	12.611414	7.768188	25.075246
H	1.744288	9.722376	22.426063
H	0.793413	10.966021	21.608673
H	1.654834	11.355448	23.092800
H	2.822180	13.316312	21.841167
H	1.928902	12.875291	20.390740
H	3.690171	12.993553	20.337305
H	3.706821	10.634586	19.329377
H	1.954280	10.593818	19.469960
H	2.910606	9.300270	20.180524
H	4.284763	13.907785	24.997635
H	4.567644	13.661650	26.714967
H	3.495570	12.569112	25.850587
H	4.873764	10.609965	26.717940
H	5.843701	11.789615	27.600128
H	6.634156	10.607538	26.554819
H	7.799362	12.583434	25.291710
H	6.977644	13.707796	26.376109
H	6.804577	13.886548	24.630036
H	10.193283	8.364175	26.151594
H	10.969811	10.246171	25.401150
H	9.498989	6.389646	21.809932
H	10.719386	8.050574	21.123739

Table S6. Geometry Optimised Coordinates for **[5˙]**$^{+}$

Pt	1.118700	-0.181200	0.000010
O	-0.416550	1.126200	0.000010
O	-0.406130	-1.487480	0.000070
N	2.667620	1.102770	-0.000010
N	2.647480	-1.481010	0.000000
C	2.539450	2.436100	-0.000040
H	1.526870	2.813590	-0.000020
C	3.643370	3.271020	-0.000080
H	3.500690	4.342180	-0.000090
C	4.912710	2.708450	-0.000100
H	5.793470	3.336220	-0.000130
C	5.041350	1.327220	-0.000080
H	6.020760	0.872080	-0.000100
C	3.901590	0.534310	-0.000040
C	3.890100	-0.932970	-0.000010
C	5.015530	-1.745920	0.000010
H	6.003040	-1.308680	0.000010
C	4.861580	-3.124710	0.000040
H	5.731070	-3.768040	0.000060
C	3.582640	-3.665240	0.000040
H	3.421650	-4.733780	0.000070
C	2.493020	-2.811600	0.000030
H	1.472390	-3.168130	0.000030
C	-1.586030	0.547920	0.000030
C	-1.576280	-0.901810	0.000040
C	-2.770680	-1.624430	0.000010
H	-2.711830	-2.703700	0.000000
C	-3.974610	-0.950530	-0.000010
C	-3.958830	0.482460	0.000010
H	-4.911530	0.983480	0.000000
C	-2.823090	1.260710	0.000030
C	-2.866050	2.794810	0.000040
C	-2.166900	3.337030	1.266460
H	-1.124190	3.030090	1.320500
H	-2.671860	2.986940	2.168860
H	-2.202270	4.428310	1.267470
C	-2.166940	3.337050	-1.266390
H	-2.202260	4.428330	-1.267360
H	-2.671960	2.987000	-2.168780
H	-1.124250	3.030060	-1.320480
C	-4.307560	3.326600	0.000070
H	-4.284400	4.416950	0.000070
H	-4.862510	3.012600	0.886050
H	-4.862560	3.012590	-0.885880
C	-5.290670	-1.737910	-0.000060
C	-6.531380	-0.831910	-0.000260
H	-6.576310	-0.196040	-0.886420
H	-6.576530	-0.195950	0.885830
H	-7.428460	-1.451800	-0.000330
C	-5.343530	-2.630030	1.260290
H	-6.275010	-3.198760	1.271310
H	-5.304190	-2.029000	2.170600
H	-4.518670	-3.342170	1.290030
C	-5.343320	-2.630270	-1.260240
H	-4.518470	-3.342420	-1.289730
H	-5.303840	-2.029400	-2.170650
H	-6.274800	-3.198990	-1.271310

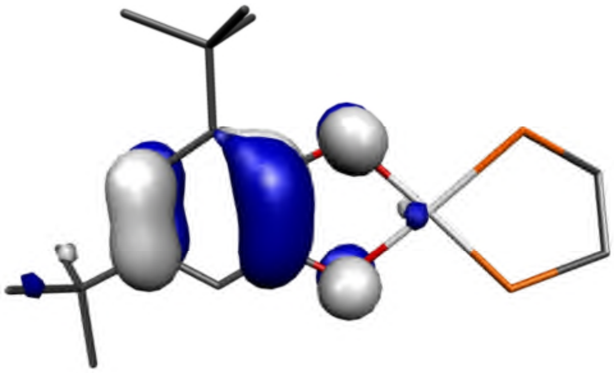

Figure S23. Isosurface plot of the SOMO in **[4$^{\bullet}$]$^{+}$.**

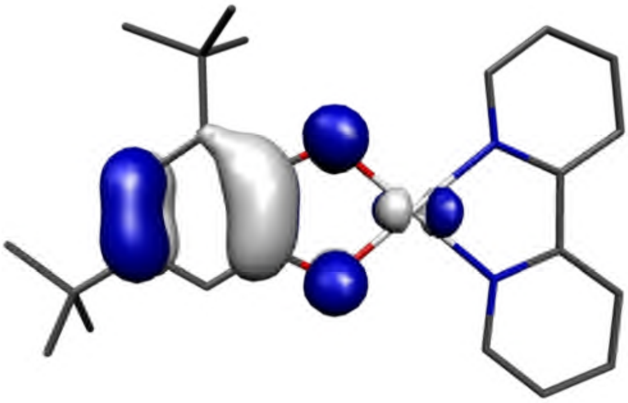

Figure S24. Isosurface plot of the SOMO in **[5$^{\bullet}$]**$^{+}$.

Table S7. Experimental and Calculated Mean Bond Distances (Å) and Angles (°) for **[1]**$^{0/1+/2+/3+}$

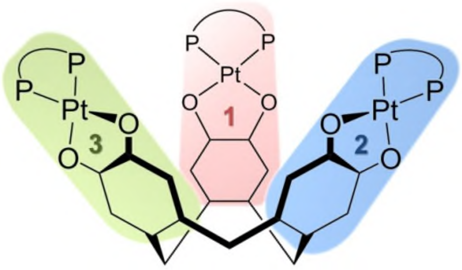

[{Pt(dppb)}$_3$(μ_3-ctc)]^z		z = 0^a exptl	z = 0 M_S = 1	z = 1+ M_S = 2	z = 2+ BS(1,1) M_S = 3	z = 3+ BS(2,1) M_S = 2
	Pt–P	2.204	2.221	2.232	2.228	2.234
	Pt–O	2.030	2.015	2.054	2.055	2.081
	C–O	1.354	1.363	1.337	1.327	1.308
**1**	C–C$_{arom}$	1.399	1.405	1.412	1.413	1.418
	τ^b	2.2	0.4	1.3	2.2	0.4
	α^c	54.0	56.5	55.9	58.9	60.8
	θ^d	0.4	0.4	2.8	4.9	4.9
	Pt–P	2.207	2.221	2.232	2.228	2.234
	Pt–O	2.038	2.017	2.047	2.056	2.080
	C–O	1.362	1.364	1.347	1.326	1.308
**2**	C–C	1.390	1.405	1.410	1.413	1.418
	τ^b	4.8	0.5	0.4	2.5	0.3
	α^c	53.2	56.5	55.9	58.9	60.8
	θ^d	6.2	0.7	1.4	4.3	1.8
	Pt–P	2.206	2.221	2.232	2.229	2.234
	Pt–O	2.032	2.017	2.047	2.056	2.081
**3**	C–O	1.351	1.364	1.346	1.326	1.308
	C–C	1.394	1.405	1.410	1.413	1.418
	τ^b	2.5	0.5	0.4	2.2	0.3
	α^c	52.7	56.5	55.9	58.9	60.8
	θ^d	3.0	0.7	1.4	5.5	2.2
	Pt···Pt	9.925	10.37	10.33	10.62	10.62
		9.880	10.37	10.33	10.69	10.83
		9.756	10.52	10.58	10.69	10.88

[a] Data are taken from ref 37. [b] Dihedral angle between the {PtO_2} and {PtP_2} mean planes. [c] Angle between the {PtO_2C_6} plane and the molecular C_3 axis. [d] Dihedral angle between the {PtO_2} and {O_2C_6} mean planes.

(37) Bohle, D. S.; Stasko, D. *Chem. Commun.* **1998**, 567.

Table S8. Geometry Optimised Coordinates for **1**

Pt	5.231565	-3.027219	0.011249
Pt	0.050749	5.950069	0.045412
Pt	-5.283168	-2.940961	0.011618
P	7.136834	-2.353165	-0.907747
P	5.632970	-4.974008	-0.979647
P	-1.448188	7.274359	-0.919225
P	1.573097	7.247263	-0.919124
P	-5.718305	-4.881740	-0.976664
P	-7.174924	-2.234255	-0.910700
O	4.854674	-1.274328	0.932682
O	3.499312	-3.598035	0.871345
O	-1.304091	4.757717	0.942496
O	1.384494	4.736278	0.945122
O	-3.561332	-3.541339	0.872714
O	-4.877112	-1.194507	0.932426
C	1.279563	-1.621992	3.078961
C	1.998808	-0.402501	3.116698
C	3.199847	-0.291895	2.394519
C	3.687828	-1.355317	1.633327
C	2.976319	-2.569657	1.598624
C	1.787742	-2.692658	2.321656
C	-0.016362	-1.852418	3.850609
C	-1.534669	0.838084	3.917968
C	1.546972	0.812645	3.919600
C	0.722640	1.832725	3.141494
C	-0.692828	1.844278	3.140512
C	-1.369970	2.830645	2.401831
C	-0.672123	3.785708	1.660052
C	0.735700	3.774285	1.661155
C	1.416832	2.807841	2.403726
C	-2.006565	-0.369482	3.115281
C	-1.308382	-1.601145	3.078823
C	-1.834814	-2.663856	2.322701
C	-3.021112	-2.521360	1.599395
C	-3.711668	-1.294965	1.632933
C	-3.205553	-0.239225	2.393035
C	7.917383	-3.704616	-1.867065
C	7.219827	-4.926268	-1.897093
C	7.748255	-6.015629	-2.604786
C	8.966386	-5.882382	-3.278705
C	9.658480	-4.666977	-3.250458
C	9.137570	-3.576948	-2.546352
C	-0.628615	8.633770	-1.835474
C	0.778267	8.621372	-1.834952
C	1.487445	9.627279	-2.507015
C	0.792663	10.642042	-3.172633
C	-0.606169	10.654270	-3.173335
C	-1.319330	9.651977	-2.508338
C	-7.303194	-4.807392	-1.895544
C	-7.979120	-3.573513	-1.867702
C	-9.196740	-3.425573	-2.547413
C	-9.736665	-4.507402	-3.249851
C	-9.065937	-5.734770	-3.276239
C	-7.850478	-5.888325	-2.601825
H	3.778844	0.634284	2.427745
H	1.260271	-3.649491	2.297257
H	-0.011673	-1.254673	4.773650
H	-0.024875	-2.905090	4.177112
H	-2.430057	1.363714	4.287806
H	-0.991282	0.505303	4.813579

H	2.450981	1.323013	4.289772
H	0.997621	0.489117	4.815023
H	-2.462078	2.867980	2.403250
H	2.509386	2.827316	2.406644
H	-1.323547	-3.629468	2.299221
H	-3.768942	0.696531	2.425478
H	7.211963	-6.966890	-2.630664
H	9.377258	-6.731100	-3.828685
H	10.608499	-4.567431	-3.778770
H	9.681762	-2.630087	-2.525964
H	2.579729	9.622229	-2.509189
H	1.345550	11.427108	-3.691736
H	-1.144681	11.448898	-3.693050
H	-2.411537	9.666206	-2.511534
H	-9.724331	-2.469308	-2.528691
H	-10.684745	-4.392060	-3.778395
H	-9.491488	-6.576974	-3.825072
H	-7.330946	-6.848898	-2.626122
H	4.722550	-5.486672	-1.949596
H	5.753691	-6.155744	-0.192111
H	8.200136	-1.895319	-0.076587
H	7.134942	-1.265564	-1.829111
H	2.468922	6.699433	-1.883571
H	2.543803	7.915741	-0.118404
H	-2.354438	6.742817	-1.883016
H	-2.405893	7.960937	-0.118149
H	-8.230241	-1.754556	-0.081695
H	-7.152087	-1.149388	-1.835001
H	-5.860692	-6.059923	-0.187393
H	-4.816134	-5.412022	-1.944858

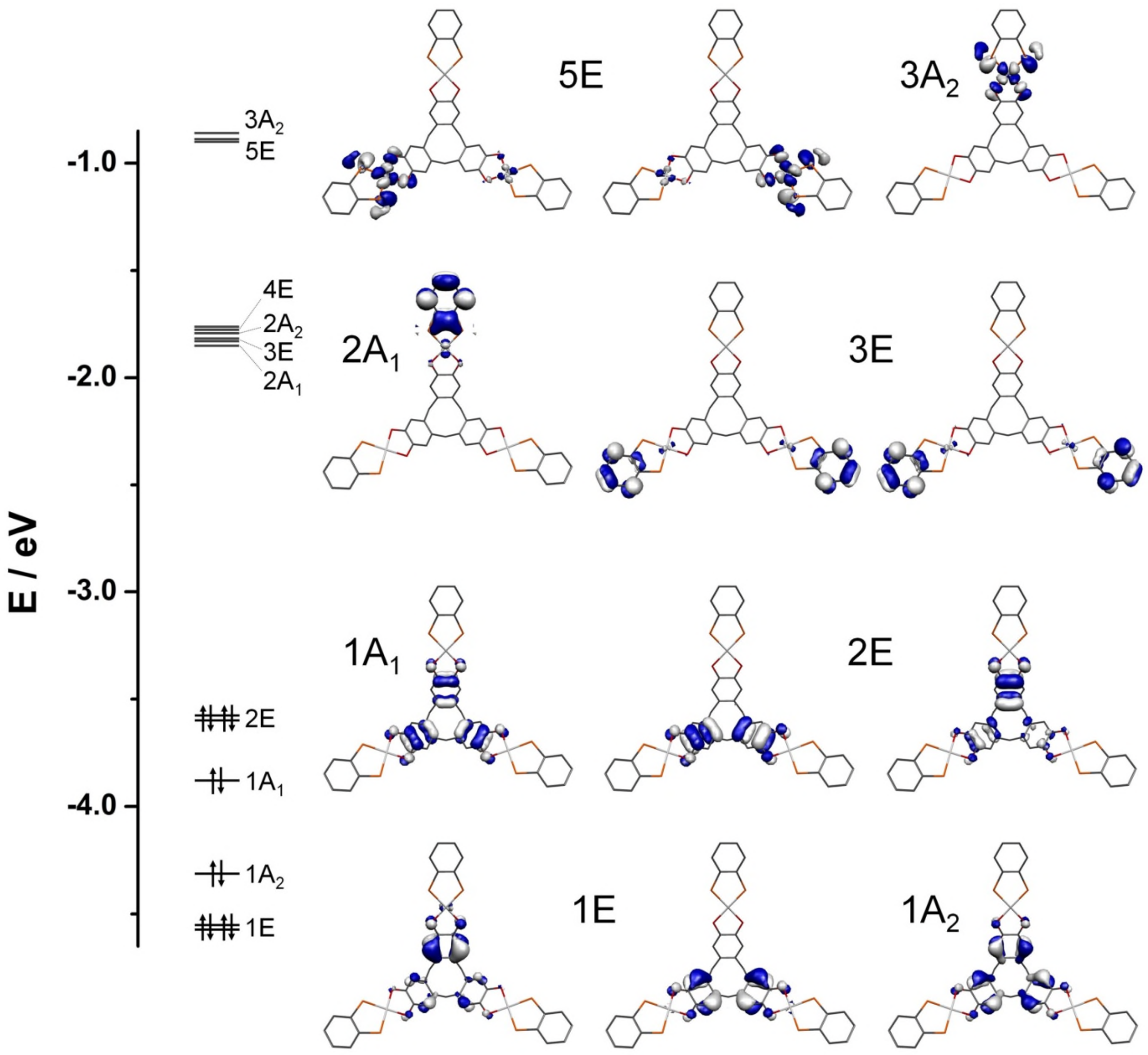

Figure S25. MO energy level scheme of frontier Kohn-Sham orbitals for **1** with C_{3v} symmetry labels.

Table S9. Geometry Optimised Coordinates for **[1•]**$^{+}$

Pt	5.264520	-3.007850	0.005420
Pt	0.050900	5.909480	-0.008050
Pt	-5.315440	-2.921200	0.004610
P	7.174470	-2.341510	-0.936830
P	5.666390	-4.967590	-0.983530
P	-1.450520	7.277610	-0.933340
P	1.576310	7.251530	-0.932210
P	-5.751320	-4.875530	-0.980580
P	-7.213130	-2.223330	-0.939580
O	4.854460	-1.232400	0.937140
O	3.501340	-3.566880	0.881830
O	-1.308350	4.659820	0.892460
O	1.387820	4.637670	0.894840
O	-3.562490	-3.509470	0.882310
O	-4.875310	-1.152000	0.934450
C	1.284410	-1.606070	3.082370
C	2.014250	-0.387810	3.139380
C	3.210650	-0.266030	2.411440
C	3.706360	-1.321300	1.636930
C	2.990670	-2.553040	1.603210
C	1.797890	-2.674260	2.330520
C	-0.016560	-1.834930	3.844470
C	-1.544160	0.840560	3.956980
C	1.555720	0.814480	3.958790
C	0.730830	1.796070	3.138910
C	-0.701940	1.807920	3.137800
C	-1.377100	2.769020	2.372700
C	-0.685690	3.714710	1.604200
C	0.748470	3.702920	1.605440
C	1.422970	2.745920	2.374950
C	-2.022330	-0.354080	3.137640
C	-1.313190	-1.584540	3.081720
C	-1.844390	-2.644460	2.330490
C	-3.034720	-2.503660	1.602690
C	-3.729250	-1.259860	1.634940
C	-3.216090	-0.212570	2.408930
C	7.944180	-3.702400	-1.882560
C	7.244210	-4.922030	-1.903400
C	7.763700	-6.017960	-2.606920
C	8.978970	-5.890690	-3.284050
C	9.676670	-4.675440	-3.262990
C	9.163140	-3.579940	-2.564770
C	-0.628030	8.635430	-1.835160
C	0.777990	8.623280	-1.834670
C	1.489670	9.631140	-2.500550
C	0.793600	10.645340	-3.162420
C	-0.607750	10.657460	-3.162910
C	-1.321720	9.655450	-2.501540
C	-7.327480	-4.803980	-1.901650
C	-8.005950	-3.572210	-1.883420
C	-9.222160	-3.429610	-2.566610
C	-9.754410	-4.517170	-3.263240
C	-9.078120	-5.744500	-3.281720
C	-7.865690	-5.891870	-2.603570
H	3.791880	0.657930	2.459490
H	1.272090	-3.631830	2.303440
H	-0.011880	-1.244030	4.770910
H	-0.025390	-2.889310	4.160610
H	-2.428880	1.383090	4.321700
H	-0.999360	0.506650	4.848590
H	2.449230	1.341790	4.324380

H	1.004540	0.489650	4.849850
H	-2.468190	2.813160	2.384250
H	2.514620	2.772020	2.388170
H	-1.334890	-3.610820	2.304470
H	-3.781700	0.721090	2.456080
H	7.223820	-6.966610	-2.626800
H	9.384370	-6.742990	-3.831000
H	10.625160	-4.581800	-3.793550
H	9.710900	-2.635700	-2.552190
H	2.581190	9.627340	-2.502810
H	1.346230	11.430840	-3.679520
H	-1.146350	11.452410	-3.680370
H	-2.413140	9.670550	-2.504530
H	-9.753250	-2.475880	-2.556050
H	-10.700780	-4.407850	-3.794560
H	-9.498060	-6.590520	-3.827450
H	-7.342550	-6.849880	-2.621420
H	4.723840	-5.448250	-1.929400
H	5.770340	-6.115510	-0.155990
H	8.207540	-1.877950	-0.081860
H	7.127640	-1.257970	-1.852300
H	2.477490	6.682390	-1.868200
H	2.501140	7.884190	-0.062740
H	-2.360560	6.724060	-1.870110
H	-2.365070	7.926020	-0.064600
H	-8.238410	-1.740390	-0.085950
H	-7.146990	-1.142350	-1.856870
H	-5.875930	-6.019790	-0.150810
H	-4.816860	-5.374640	-1.924900

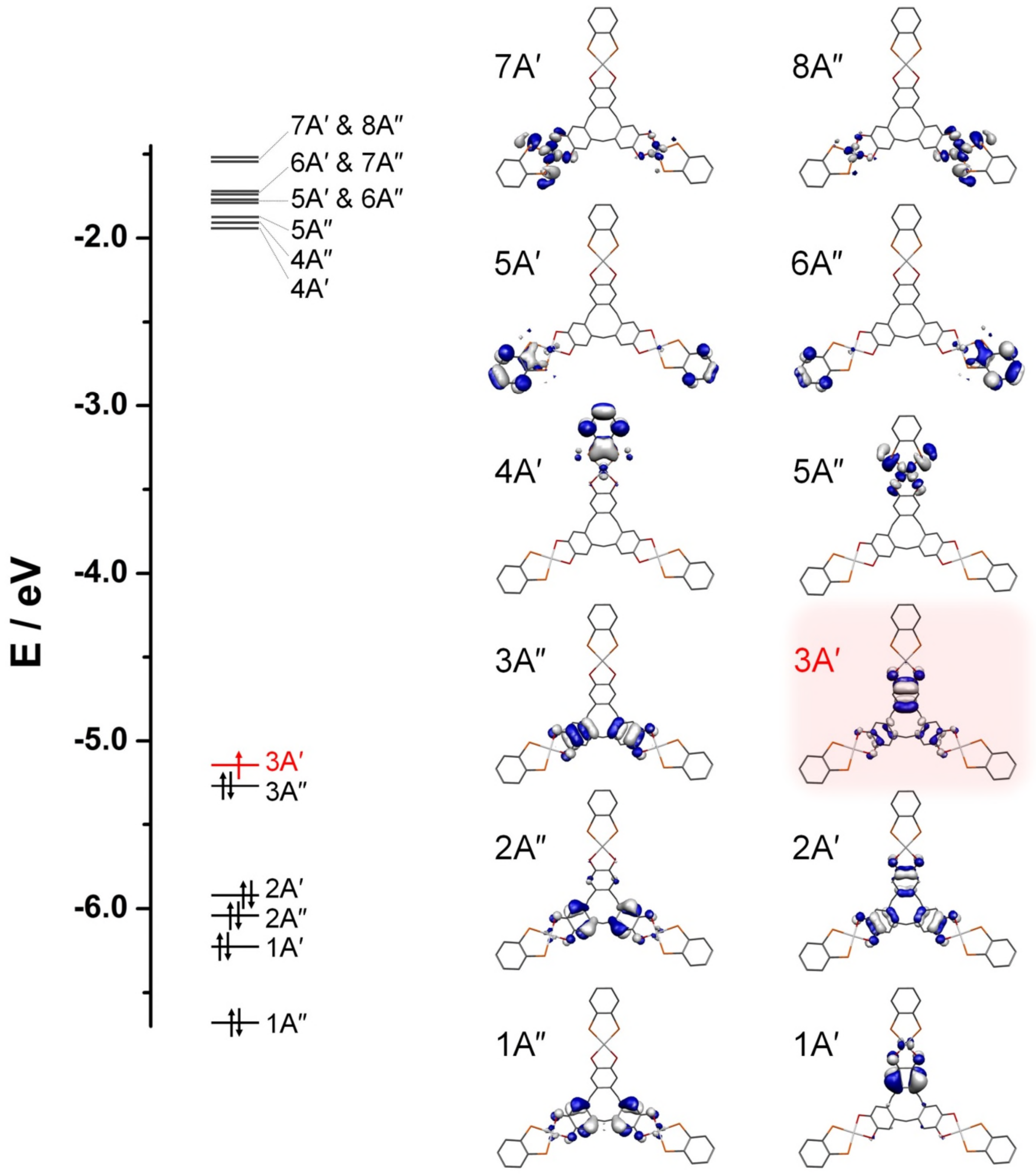

Figure S26. MO energy level scheme of frontier Kohn-Sham orbitals for **[1$^{\bullet}$]$^{+}$** with C_s symmetry labels.

Table S10. Geometry Optimised Coordinates for **[1••]**$^{2+}$

Pt	5.958885	-1.635666	-0.147830
Pt	-1.585525	5.934305	-0.103253
Pt	-4.391874	-4.307855	-0.090475
P	7.753457	-0.553786	-0.904603
P	6.963932	-3.466875	-0.925203
P	-3.424054	6.943557	-0.855199
P	-0.504106	7.706485	-0.913097
P	-4.365318	-6.381420	-0.906473
P	-6.483320	-4.231814	-0.852633
O	5.022643	0.015328	0.639128
O	4.309253	-2.579767	0.634215
O	-2.534592	4.304797	0.711089
O	0.068684	4.985687	0.663809
O	-2.488921	-4.340112	0.687563
O	-4.379707	-2.423419	0.727904
C	1.653278	-1.203733	2.746057
C	2.031471	0.183758	2.743358
C	3.151415	0.590347	2.017187
C	3.922313	-0.332548	1.293215
C	3.541871	-1.721341	1.292453
C	2.410756	-2.124170	2.020272
C	0.488099	-1.739287	3.566164
C	-1.681352	0.459489	3.606325
C	1.300523	1.241548	3.557331
C	0.240141	1.991256	2.764247
C	-1.150971	1.629372	2.790759
C	-2.076300	2.400954	2.086877
C	-1.672964	3.527698	1.353366
C	-0.279783	3.890688	1.326618
C	0.647191	3.108319	2.033534
C	-1.819607	-0.821625	2.796949
C	-0.809379	-1.845349	2.776992
C	-1.033316	-3.017887	2.053970
C	-2.229756	-3.218733	1.346942
C	-3.241017	-2.193739	1.368227
C	-3.009268	-1.015000	2.094018
C	8.998420	-1.719639	-1.548274
C	8.630055	-3.078741	-1.558285
C	9.528453	-4.046123	-2.029449
C	10.789047	-3.651839	-2.483662
C	11.155321	-2.300268	-2.473238
C	10.263961	-1.330669	-2.008613
C	-3.035716	8.591804	-1.531688
C	-1.673327	8.946727	-1.561521
C	-1.284009	10.197336	-2.061089
C	-2.256374	11.087013	-2.523380
C	-3.611253	10.734503	-2.492287
C	-4.006005	9.488543	-1.999798
C	-5.999193	-6.844013	-1.572348
C	-6.987451	-5.841028	-1.546157
C	-8.275887	-6.110682	-2.027862
C	-8.571477	-7.379861	-2.530134
C	-7.588839	-8.377269	-2.556859
C	-6.302036	-8.114636	-2.081288
H	3.481946	1.630429	2.038477
H	2.169002	-3.188374	2.045217
H	0.341900	-1.132345	4.468909
H	0.755584	-2.747174	3.915998
H	-2.677797	0.732137	3.983447
H	-1.056050	0.290696	4.492020
H	2.044155	1.973553	3.904617

H	0.864688	0.799251	4.462440
H	-3.142585	2.171191	2.128766
H	1.691464	3.425986	2.034540
H	-0.299830	-3.826219	2.061107
H	-3.806030	-0.269990	2.130667
H	9.252662	-5.102644	-2.038780
H	11.492257	-4.403544	-2.845411
H	12.143174	-2.000968	-2.826575
H	10.558654	-0.279129	-2.001075
H	-0.229954	10.481726	-2.086294
H	-1.956396	12.063098	-2.907587
H	-4.364984	11.436548	-2.851972
H	-5.064873	9.222615	-1.977437
H	-9.048816	-5.339444	-2.007839
H	-9.575225	-7.594104	-2.900257
H	-7.828440	-9.367224	-2.947905
H	-5.543198	-8.899502	-2.103636
H	6.322141	-4.176282	-1.975679
H	7.152742	-4.524289	0.002814
H	8.444448	0.249302	0.039951
H	7.561141	0.398969	-1.940722
H	0.427888	7.486151	-1.962470
H	0.321346	8.412602	0.000625
H	-4.163510	6.285957	-1.874650
H	-4.457106	7.160365	0.093925
H	-7.486905	-3.900605	0.094992
H	-6.779283	-3.279253	-1.864176
H	-4.026088	-7.414338	0.006110
H	-3.443238	-6.665039	-1.949390

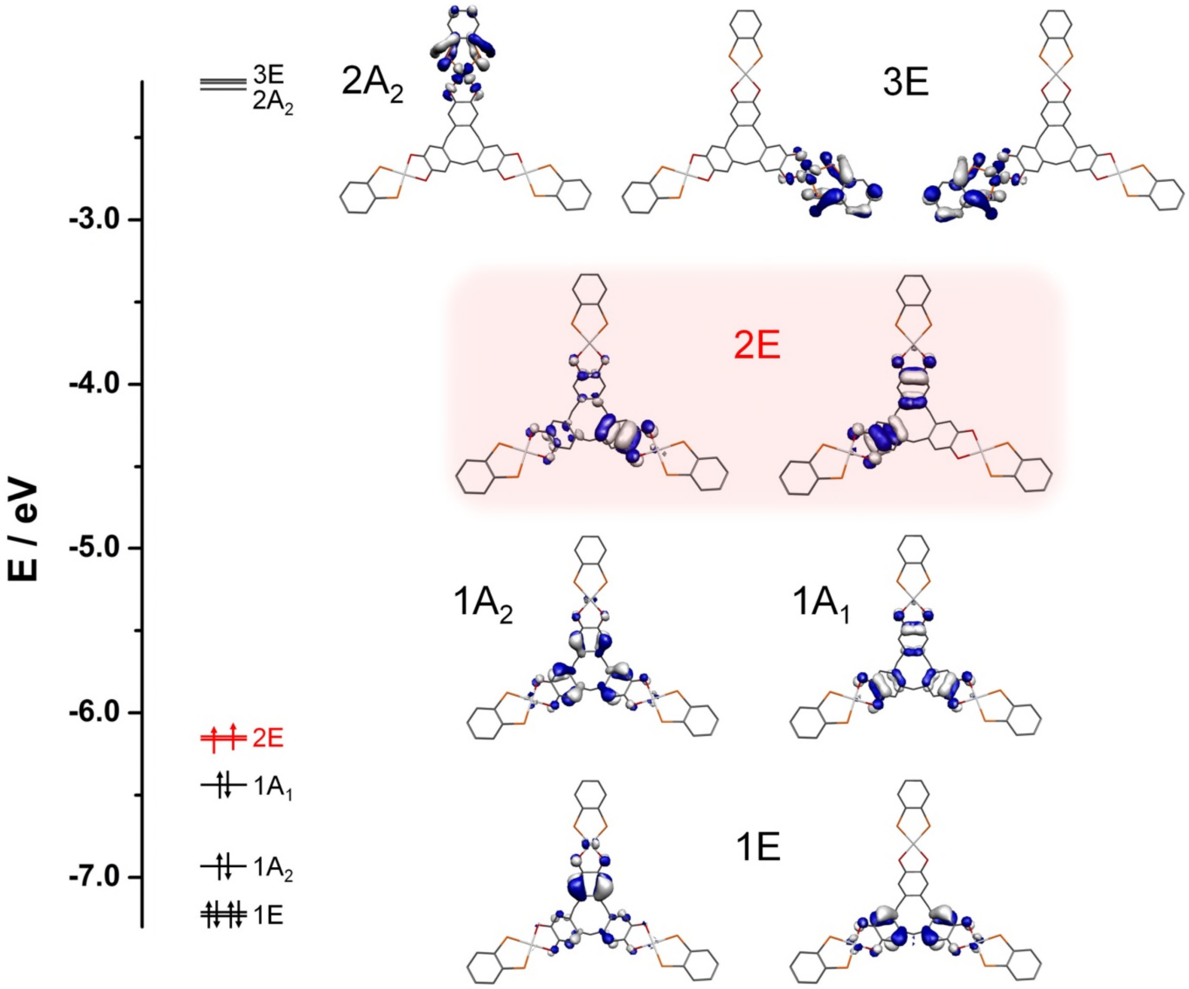

Figure S27. MO energy level scheme of frontier Kohn-Sham orbitals for **[1**$^{\bullet\bullet}$**]**$^{2+}$ with C_{3v} symmetry labels.

Table S11. Geometry Optimised Coordinates for $[\mathbf{1}^{\bullet\bullet\bullet}]^{3+}$

Pt	6.097377	-1.660597	-0.081560
Pt	-1.618070	6.027604	-0.069066
Pt	-4.491084	-4.379893	-0.058747
P	7.915881	-0.580983	-0.800000
P	7.125351	-3.490790	-0.845591
P	-3.458347	7.066103	-0.792528
P	-0.539165	7.820474	-0.850997
P	-4.492129	-6.473572	-0.837178
P	-6.608240	-4.327671	-0.765835
O	5.110913	-0.005120	0.700210
O	4.389916	-2.592814	0.655538
O	-2.553247	4.347092	0.720434
O	0.045476	5.026881	0.679735
O	-2.533863	-4.360535	0.659086
O	-4.425764	-2.454631	0.729802
C	1.670738	-1.225270	2.662364
C	2.053847	0.172806	2.674418
C	3.200016	0.580038	2.006000
C	4.003246	-0.346392	1.305349
C	3.610813	-1.747485	1.279770
C	2.440565	-2.145604	1.962389
C	0.486269	-1.754084	3.463299
C	-1.689508	0.448818	3.527254
C	1.292161	1.225021	3.469399
C	0.233049	1.992892	2.689082
C	-1.169814	1.630857	2.718404
C	-2.098797	2.408351	2.038639
C	-1.702331	3.563684	1.331761
C	-0.294721	3.933337	1.310728
C	0.639209	3.124216	1.994872
C	-1.836946	-0.827568	2.711132
C	-0.812426	-1.857428	2.673210
C	-1.041290	-3.029244	1.963457
C	-2.261654	-3.247957	1.287841
C	-3.288727	-2.214937	1.327439
C	-3.037153	-1.023801	2.040647
C	9.135891	-1.733185	-1.496435
C	8.767512	-3.092909	-1.515868
C	9.648658	-4.053905	-2.030164
C	10.892308	-3.648873	-2.521447
C	11.258110	-2.297012	-2.502741
C	10.384755	-1.333705	-1.991841
C	-3.063268	8.695482	-1.493841
C	-1.699168	9.046793	-1.523811
C	-1.301695	10.285015	-2.046759
C	-2.271062	11.164793	-2.534647
C	-3.627193	10.815913	-2.503888
C	-4.030336	9.582691	-1.985635
C	-6.110569	-6.918470	-1.533919
C	-7.099062	-5.914930	-1.501298
C	-8.378409	-6.168230	-2.014558
C	-8.661453	-7.423912	-2.557572
C	-7.679072	-8.421821	-2.589772
C	-6.401762	-8.176177	-2.079714
H	3.535287	1.617916	2.045709
H	2.195609	-3.209132	1.975411
H	0.337873	-1.152613	4.368865
H	0.744845	-2.764226	3.811297
H	-2.682368	0.716957	3.915102
H	-1.057912	0.274597	4.406801
H	2.027464	1.957542	3.831638

H	0.849509	0.774824	4.367368
H	-3.164679	2.177961	2.081844
H	1.683279	3.442155	1.998785
H	-0.307802	-3.837340	1.963123
H	-3.839647	-0.286755	2.098996
H	9.374364	-5.110644	-2.048027
H	11.582248	-4.393563	-2.921044
H	12.232107	-1.991342	-2.887592
H	10.680217	-0.282536	-1.979756
H	-0.247213	10.567545	-2.073323
H	-1.966730	12.130850	-2.940062
H	-4.376553	11.510938	-2.885491
H	-5.090309	9.321174	-1.964960
H	-9.151829	-5.397718	-1.992390
H	-9.656082	-7.626361	-2.957440
H	-7.910658	-9.399510	-3.014771
H	-5.644043	-8.961868	-2.108427
H	6.437996	-4.206112	-1.860416
H	7.318029	-4.515105	0.117000
H	8.592608	0.174315	0.192114
H	7.692509	0.415295	-1.785577
H	0.418240	7.569699	-1.868100
H	0.260244	8.501526	0.102821
H	-4.208471	6.373581	-1.778370
H	-4.453417	7.279919	0.195913
H	-7.567700	-4.029933	0.236039
H	-6.907202	-3.326813	-1.726325
H	-4.175650	-7.468845	0.123118
H	-3.528540	-6.755000	-1.840079

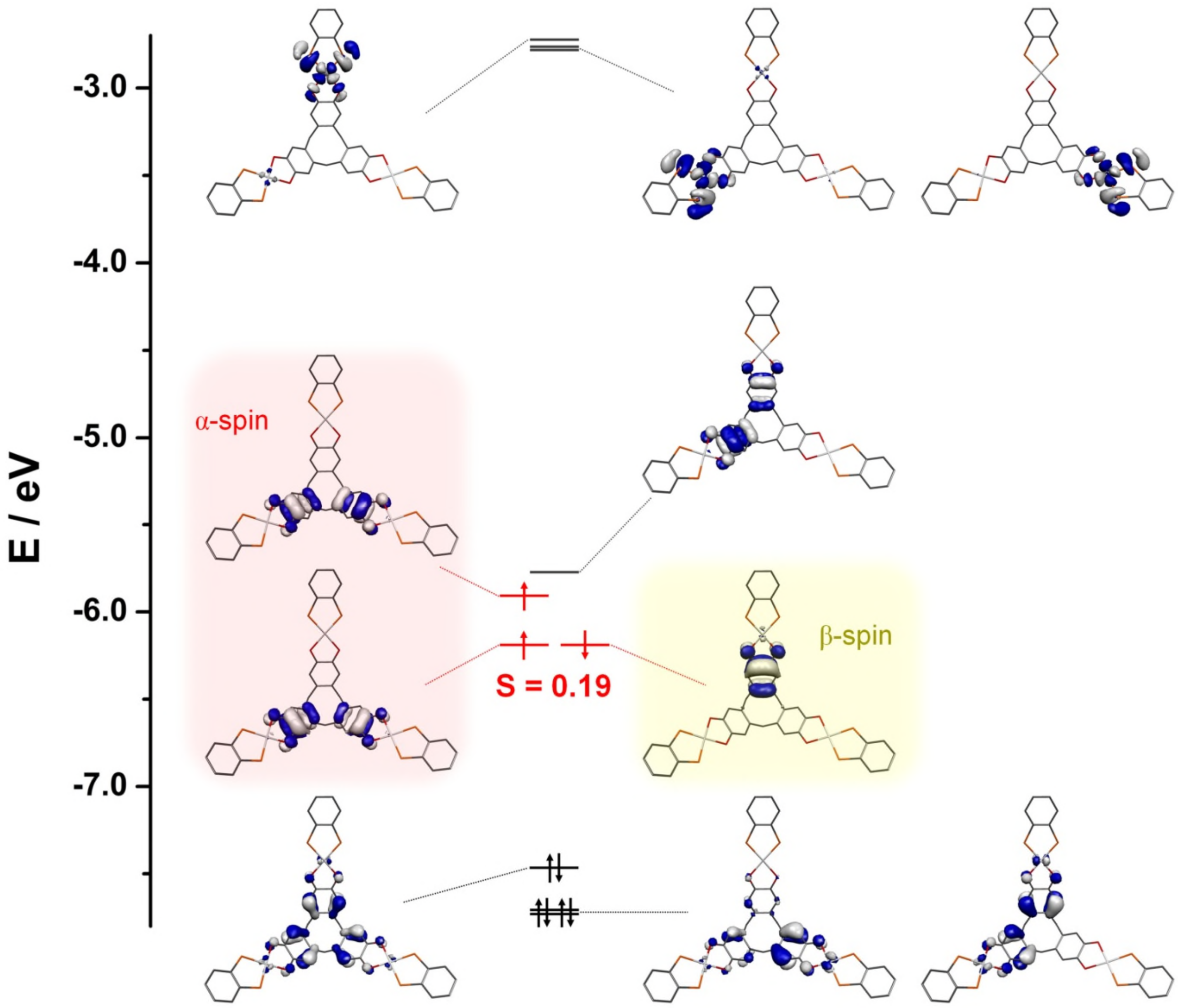

Figure S28. MO energy level scheme of frontier Kohn-Sham orbitals for **[1•••]**$^{3+}$. α-spin and β-spin magnetic orbitals (SOMOs) are highlighted red and yellow, respectively.

Table S12. Mean Bond Distances (Å) and Angles (°) in Geometry Optimised **[3•]**$^{+}$

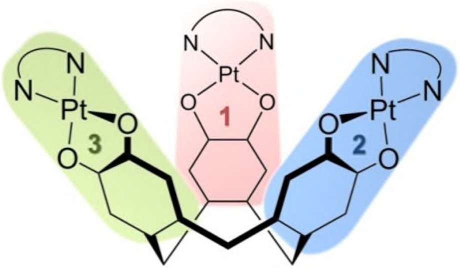

		$[\{Pt(bipy)\}_3(\mu_3\text{-}ctc)]^{+}$ $M_S = 2$
**1**	Pt–N	2.006
	Pt–O	1.997
	C–O	1.329
	$C–C_{arom}$	1.403
	τ^a	0.4°
	α^b	55.9°
	θ^c	2.4°
**2**	Pt–N	2.005
	Pt–O	1.994
	C–O	1.336
	C–C	1.400
	τ^a	0.4°
	α^b	57.8°
	θ^c	1.5°
**3**	Pt–N	2.005
	Pt–O	1.994
	C–O	1.336
	C–C	1.400
	τ^a	0.4°
	α^b	57.8°
	θ^c	1.5°
	Pt···Pt	10.241
		10.241
		10.544

^{a} Dihedral angle between the $\{PtO_2\}$ and $\{PtN_2\}$ mean planes. ^{b} Angle between the $\{PtO_2C_6\}$ plane and the molecular C_3 axis. ^{c} Dihedral angle between the $\{PtO_2\}$ and $\{O_2C_6\}$ mean planes.

Table S13. Geometry Optimised Coordinates for [**3**•]$^+$

Pt	5.271890	2.926670	-0.035900
Pt	0.000590	-5.853230	-0.044580
Pt	-5.272520	2.925720	-0.035800
O	1.321720	-4.636590	0.828260
O	-1.320780	-4.636820	0.828250
O	-4.870890	1.203570	0.884800
O	-3.540520	3.488480	0.773420
O	4.870610	1.204450	0.884770
O	3.539720	3.489000	0.773280
C	2.016860	0.372180	3.033060
C	1.296620	1.588430	2.958220
C	1.814780	2.638340	2.203940
C	3.007200	2.503510	1.498860
C	3.718070	1.279790	1.558750
C	3.213970	0.238970	2.327870
H	1.299210	3.589730	2.159960
H	3.789860	-0.675950	2.391960
C	-0.000180	1.835180	3.712530
H	-0.000130	1.264530	4.640080
H	-0.000250	2.884630	4.013570
C	-1.296970	1.588240	2.958240
C	-1.815370	2.638090	2.204030
C	-2.016930	0.371850	3.033050
C	-3.007770	2.503040	1.498970
H	-1.299980	3.589580	2.160090
C	-3.214030	0.238430	2.327880
C	-3.718350	1.279180	1.558800
H	-3.789740	-0.676610	2.391930
C	1.546370	-0.816560	3.856740
H	1.003650	-0.485180	4.738500
H	2.427030	-1.345210	4.225160
C	0.714300	-1.793770	3.046970
C	-0.713920	-1.793870	3.046920
C	1.394750	-2.747020	2.294820
C	-1.394180	-2.747230	2.294760
C	0.712790	-3.692940	1.538100
H	2.476460	-2.782040	2.303540
C	-0.712030	-3.693060	1.538080
H	-2.475890	-2.782410	2.303410
C	-1.546180	-0.816810	3.856680
H	-1.003520	-0.485330	4.738430
H	-2.426720	-1.345660	4.225110
C	-6.901590	4.643430	-1.664630
C	-4.958510	5.737980	-0.947740
C	-7.312070	5.785340	-2.347100
C	-7.637900	3.383020	-1.601640
C	-5.323730	6.894500	-1.608070
H	-4.044940	5.650150	-0.375550
C	-6.520190	6.920020	-2.320720
H	-8.244640	5.783940	-2.892370
C	-8.862110	3.131520	-2.215130
H	-4.678390	7.760140	-1.561050
C	-7.603320	1.212490	-0.720340
C	-9.458190	1.891160	-2.069350
H	-9.343060	3.902130	-2.799750
C	-8.817100	0.916390	-1.308470
H	-7.052570	0.504100	-0.116420
H	-9.251150	-0.063450	-1.168690
N	-5.727650	4.637510	-0.974980
N	-7.025970	2.416550	-0.863140
C	-0.730800	-8.124440	-1.644160

C	-2.623110	-6.996420	-0.849430
C	-1.536820	-9.054750	-2.292270
C	0.732440	-8.124220	-1.644260
C	-3.465410	-7.895570	-1.475230
H	-2.985410	-6.162470	-0.263740
C	-2.914500	-8.941910	-2.208670
H	-1.089560	-9.860440	-2.855860
C	1.538660	-9.054170	-2.292630
H	-4.535390	-7.773520	-1.385050
H	-3.550670	-9.660300	-2.707650
C	2.624510	-6.995680	-0.849670
C	2.916320	-8.940900	-2.209220
H	1.091580	-9.859910	-2.856290
C	3.467010	-7.894500	-1.475710
H	2.986630	-6.161690	-0.263940
H	4.536960	-7.772130	-1.385660
N	-1.288280	-7.108100	-0.931800
N	1.289710	-7.107770	-0.931880
C	7.637390	3.384560	-1.601380
C	7.603280	1.214060	-0.719960
C	8.861890	3.133470	-2.214450
C	6.900650	4.644700	-1.664690
C	8.817350	0.918360	-1.307700
H	7.052610	0.505500	-0.116190
C	9.458360	1.893320	-2.068390
H	9.342770	3.904220	-2.798930
C	7.310770	5.786580	-2.347420
H	9.251700	-0.061320	-1.167740
C	4.957100	5.738720	-0.948280
C	6.518470	6.920980	-2.321430
H	8.243390	5.785370	-2.892620
C	5.321950	6.895200	-1.608890
H	4.043490	5.650730	-0.376160
H	4.676290	7.760610	-1.562160
N	7.025580	2.417930	-0.862990
N	5.726630	4.638520	-0.975170
H	3.552640	-9.659000	-2.708410
H	-10.409970	1.685400	-2.540060
H	-6.830190	7.813120	-2.846100
H	6.828180	7.814040	-2.847050
H	10.410380	1.687880	-2.538770

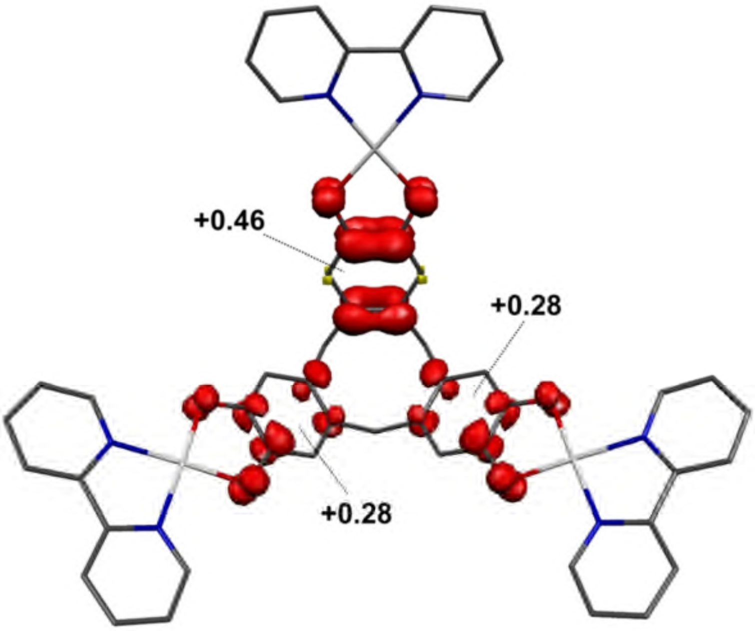

Figure S29. Mulliken spin density map for [**3**]$^+$ (red: α-spin; yellow: β-spin).

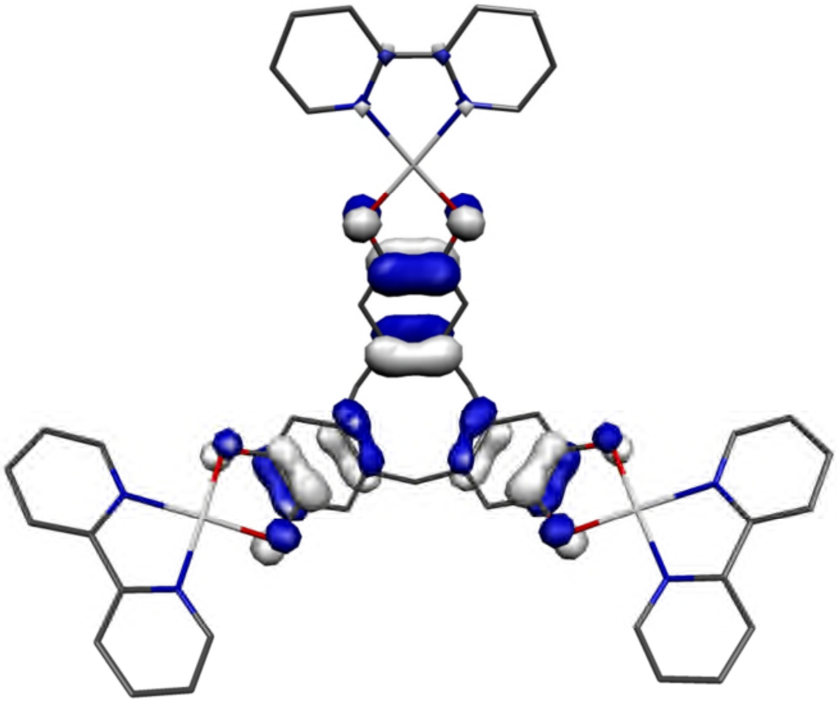

Figure 30. Isosurface plot of the ground state molecular orbital of [**3$^\bullet$**]$^+$.

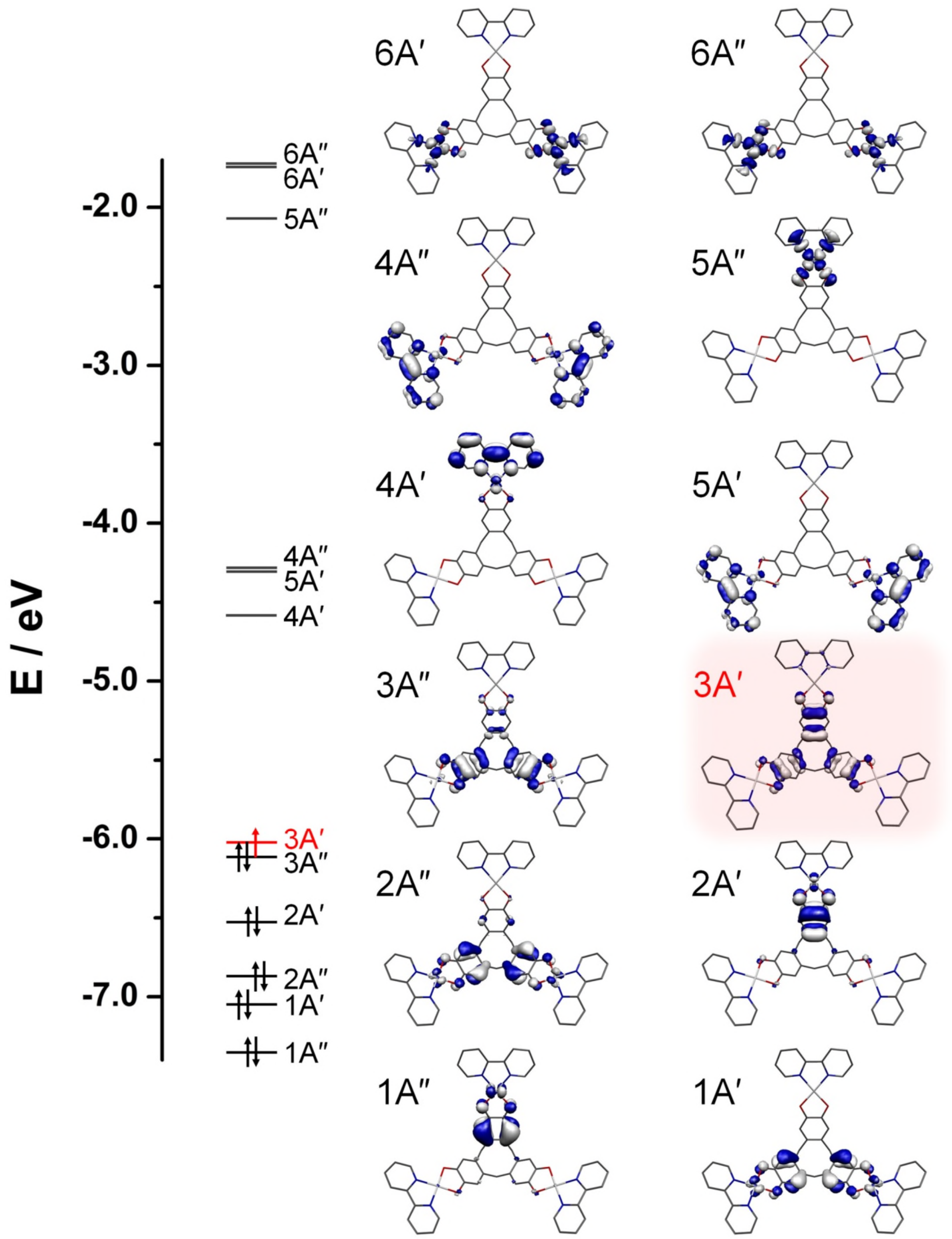

Figure S31. MO energy level scheme of frontier Kohn-Sham orbitals for [**3**$^{\bullet}$]$^{+}$ with C_s symmetry labels.

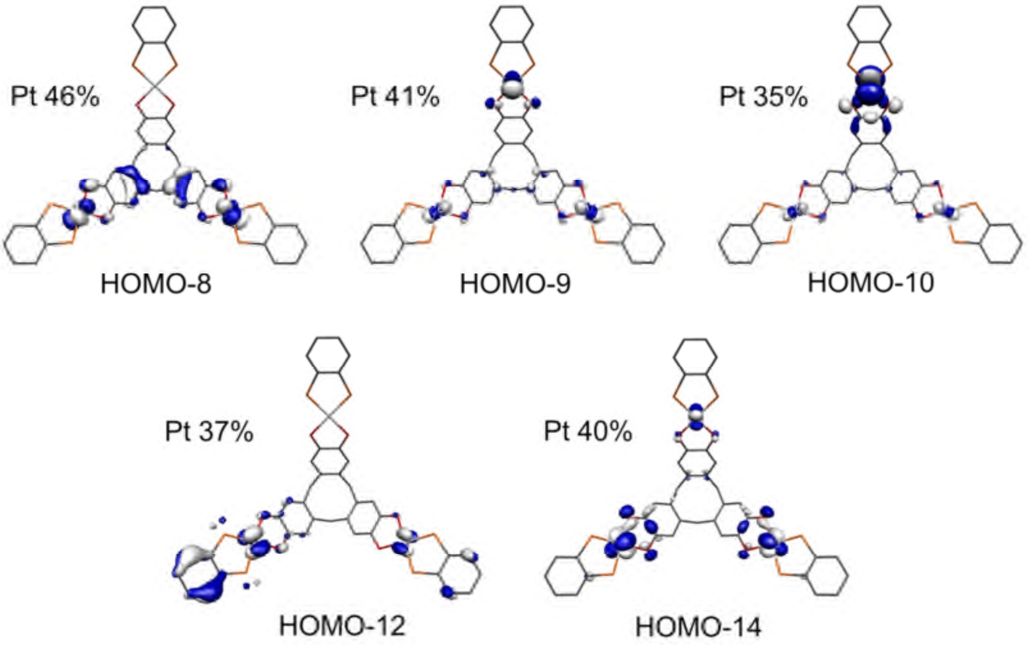

Figure S32. Orbitals involved in electronic transitions on the β manifold of $[\mathbf{1}^{\bullet}]^{+}$.

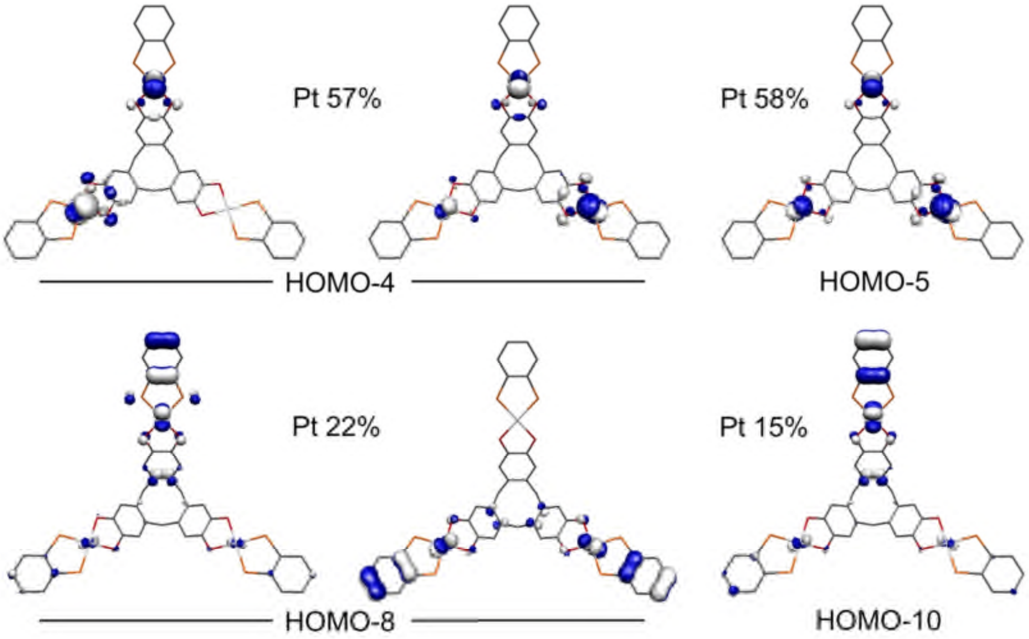

Figure S33. Orbitals involved in electronic transitions on the β manifold of $[\mathbf{1}^{\bullet\bullet}]^{2+}$.

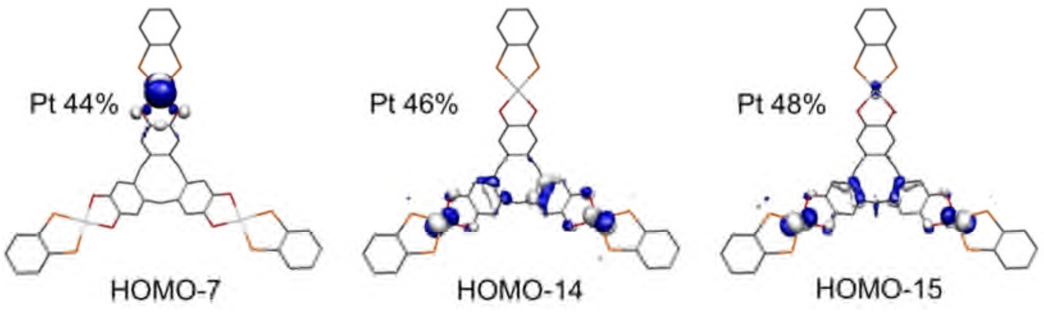

Figure S34. Orbitals involved in electronic transitions on the β manifold of [**1**$^{\bullet\bullet\bullet}$]$^{3+}$.
